# Human population history revealed by a supertree approach

**DOI:** 10.1038/srep29890

**Published:** 2016-07-19

**Authors:** Pavel Duda, Jan Zrzavý

**Affiliations:** 1 Department of Zoology, Faculty of Science, University of South Bohemia, České Budějovice, Czech Republic; 2 Center for Theoretical Study, Charles University and Academy of Sciences of the Czech Republic, Prague, Czech Republic

## Abstract

Over the past two decades numerous new trees of modern human populations have been published extensively but little attention has been paid to formal phylogenetic synthesis. We utilized the “matrix representation with parsimony” (MRP) method to infer a composite phylogeny (supertree) of modern human populations, based on 257 genetic/genomic, as well as linguistic, phylogenetic trees and 44 admixture plots from 200 published studies (1990–2014). The resulting supertree topology includes the most basal position of S African Khoisan followed by C African Pygmies, and the paraphyletic section of all other sub-Saharan peoples. The sub-Saharan African section is basal to the monophyletic clade consisting of the N African–W Eurasian assemblage and the consistently monophyletic Eastern superclade (Sahul–Oceanian, E Asian, and Beringian–American peoples). This topology, dominated by genetic data, is well-resolved and robust to parameter set changes, with a few unstable areas (e.g., West Eurasia, Sahul–Melanesia) reflecting the existing phylogenetic controversies. A few populations were identified as highly unstable “wildcard taxa” (e.g. Andamanese, Malagasy). The linguistic classification fits rather poorly on the supertree topology, supporting a view that direct coevolution between genes and languages is far from universal.

Evolutionary history of modern human populations is an extensively studied topic of great complexity. Human population history is certainly not purely phylogenetic, or tree-like^1^, as genetic admixture, mediated by processes such as migrations, expansions, intermarriage, trade, or slavery, have played an important role in shaping human history^2^. There is, however, a strong hierarchical signal that can be hypothesized as phylogeny in both genetic^3^,^4^ and cultural (especially linguistic) data^5^,^6^. It is worth noting that even using such terms as “genetic admixture” and “horizontal gene flow” implies an assumption of an underlying tree-like model^7^. Recently developed phylogenetic methods applied to both genetic^8^,^9^ and linguistic data^10^ allow us to visualize evolutionary history of populations using a bifurcating tree with horizontal links (“admixture edges”), accounting for both population splits and mixtures.

Today, no unified picture of modern human evolution based on genetic data is available, as studies that infer human population history have used different types of genetic markers, from “classical polymorphisms” (such as AB0 blood groups and protein allomorphisms) and uniparental markers (the mitochondrial DNA and the non-recombining portion of the Y chromosome) to genome-wide allele frequency data and data based on whole-genome sequencing^11^. Moreover, individual studies only partially overlap taxonomically. Even the largest published tree (267 populations) based on genome-wide data^12^ lacks several population groups important for a comprehensive description of human population history on a global scale (e.g., populations of N Africa, Anatolia, Balkans, E Europe, Indonesia, N Asia, Beringia, and N America). A recent meta-analysis of human genomic diversity projects^13^ has also pointed to the lack of several key population groups (e.g., Hadza, Sandawe, Fulani, Chadic speakers, Australian Aboriginals, populations of Indonesia, Polynesia, and Northern America).

The language phylogenies published to date include up to 542 language varieties^14^ but usually cover just one language family each (mostly Bantu, Indo-European, or Austronesian). Formal attempts to reconstruct genealogical relationships between languages beyond the level of the families have been rare so far^15^,^16^, and nearly all of the proposed linguistic macrofamilies such as Eurasiatic/Nostratic^17^,^18^,^19^, Indo-Pacific^20^, and Amerind^21^ are considered controversial^16^.

Although a large body of comparative data currently exists for a phylogenetic synthesis, integration of all kinds of raw data using a “supermatrix approach” (or “total evidence approach”^22^) remains unfeasible for the human population, particularly due to the distance-based (instead of character-based) nature of some source data and lack of widely overlapping datasets. In light of these problems, a possible strategy is to focus on published (“source”) trees, adopting the “supertree approach” (or “taxonomic congruence approach”^23^). The primary application of supertrees is to summarize existing phylogenetic hypotheses in a form of a synthetic consensus which can be used to identify and evaluate topological conflicts caused by incongruent or missing data^24^. In the “matrix representation with parsimony” (MRP) method^25^,^26^, each source tree is converted into a matrix of additive binary characters; the individual matrices are eventually merged into a single character matrix which is then analyzed by the maximum parsimony (MP) method to obtain a composite phylogeny. The resulting supertree is analogous to a consensus tree when the source trees have different sets of taxa^24^.

The aims of this study are:

1. to provide a well corroborated phylogenetic hypothesis on human population group-level relationships, based on both genetic and linguistic data;
2. to assess for the first time the utility of admixture plots, produced by STRUCTURE, FRAPPE, and ADMIXTURE software, as sources of hierarchical information during the supertree construction;
3. to assess the stability of the inferred supertree topology and to identify populations whose phylogenetic position is particularly unstable;
4. to compare the topologies based on genetic and linguistic data, and evaluate their relative influence on the supertree topology; and
5. to test for congruence between proposed linguistic groupings (language families and macrofamilies) and supertree topology and to infer the relationships between language families by constraining the supertree topology with linguistic classification.

## Results and Discussion

### Supertree construction

Altogether 257 source trees (obtained by using both distance-based and character-based methods) and 44 admixture plots from 200 published studies (1990–2014) contributed to the resulting supertree dataset. They included trees based on genomic data, including both genome-wide allele frequency data and whole-genome sequences (51 trees from 33 studies), genetic trees based on autosomal data (26, 19), Y-chromosomal data (9, 9), mtDNA (25, 20), human leukocyte antigen (HLA) system (75, 57), “classical polymorphisms” (27, 8), language trees based on lexical or structural data (44, 33), admixture plots based on genomic data (43, 36), and one admixture plot based on linguistic structural data.

The resulting supertree dataset (unpublished) included 973 populations and 5 great apes or archaic hominins that featured in the source trees (see Supplementary methods). Two datasets were then created based on restricted samples of this dataset. The first dataset consisted of 186 populations and included all world regions and major linguistic groups that are reasonably well represented throughout the source trees (“representative dataset” hereafter) (Supplementary Table S2). The second dataset consisted of 52 populations from the Human Genome Diversity Project (HGDP) panel^3^ that are best represented throughout the source trees, plus three additional populations to represent Australia, Micronesia and Polynesia (“HGDP dataset” hereinafter).

To investigate robustness of the inferred supertree topology, we used a method inspired by the “sensitivity analysis” of Wheeler^27^. The analysis was carried out by successively reweighting and rerooting the data partitions, adjusting an effect of different data partitions on the resulting supertree topology. In this study, a sensitivity analysis has been used for the first time for supertree inference. We used 16 sets of parameters for both representative and HGDP samples, based on combinations of four weighting and four rooting schemes (see Methods).

Sixteen sets of the most parsimonious (MP) trees, recovered in a sensitivity analysis of the representative dataset, were analyzed using the *Iter*PCR method^28^,^29^ in order to identify unstable (“wildcard”^30^) taxa which cause large polytomies in the supertree, hampering the interpretation of phylogenetic results (see Methods). Four wildcards that decreased resolution of the supertree by five or more nodes (see below) were excluded from the dataset, and the pruned version of the representative dataset (182 populations) was used for subsequent analyses.

### Supertree topologies and topological stability

**Figure 1:**
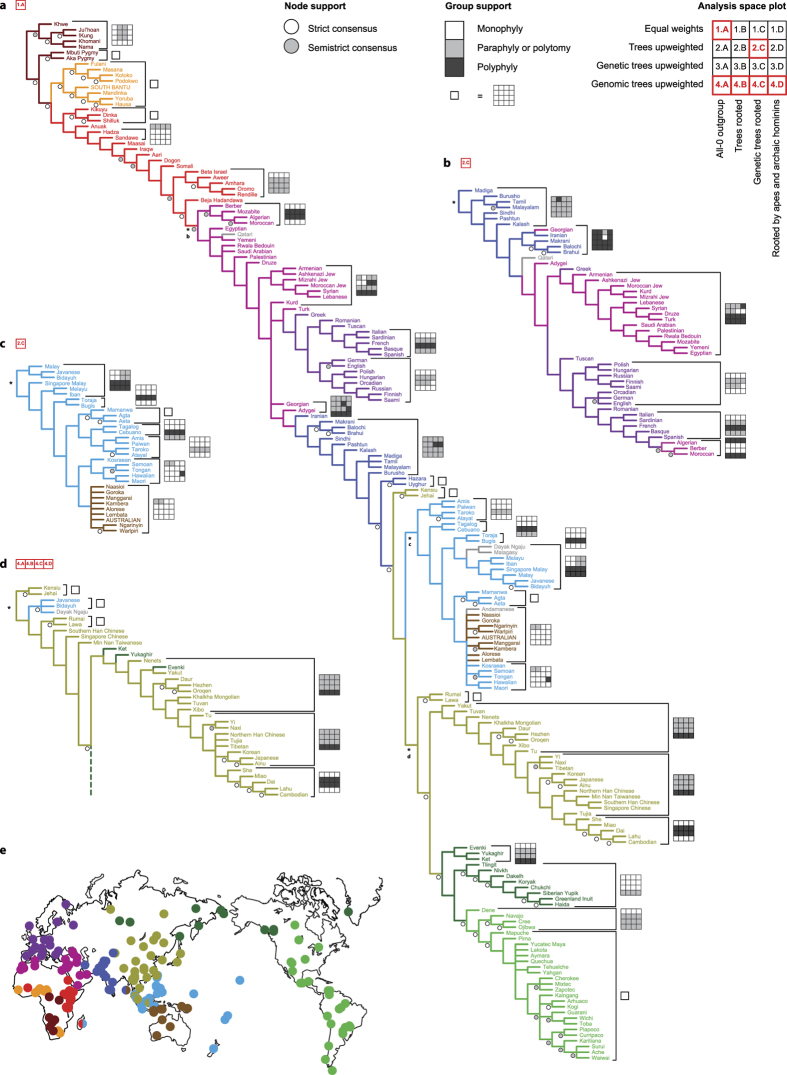
(**a**) Semistrict consensus supertree of 186 human populations (outgroups not shown) based on the representative dataset and parameter set 1.A of the sensitivity analysis (all data partitions were weighted equally and all sources were considered rooted). SOUTH BANTU = Ndebele + Swati + Xhosa + Zulu (often occurred as a composite population in the source trees); AUSTRALIAN consists of Australian Aboriginal populations of unspecified ethnic origin. The color code corresponds to the recovered monophyletic or paraphyletic groups of populations. The wildcard taxa (Qatari, Andamanese, Malagasy, Dayak Ngaju) are displayed (in gray) in the most basal of all positions they acquired when included into the dataset, but were not taken into account when assessing node and group support. The circles indicate presence of the nodes in the strict (white) and semistrict (gray) consensus of 16 supertrees derived from the sensitivity analysis (a circle is absent if the respective node is absent even in the semistrict consensus). The analysis space plots (square grids) describe presence of the selected clades/groups in the supertree under individual parameter sets as either: a monophyletic clade (white); a paraphyletic group or an unresolved section compatible with monophyly or paraphyly (gray); a polyphyletic assemblage (black). Completely white grids (=the group present under all parameter sets) are substituted by small white squares. (**b, c**) Alternative topology for the N African–W Eurasian assemblage and the Sahul–Oceanian clade as recovered in parameter set 2.C. (**d**) Alternative topology for the E Asia clade as recovered in parameter sets 4.A–4.D. The nodes where the alternative topologies (**b, c, d**) begin in the supertree 1.A (**a**) are denoted by asterisks. (**e**) Geographic locations of 186 human populations plotted on the world map using QGIS v.2.8 (the color code corresponds to the trees).

Given the expected conflict across different types of data, the resulting supertree topologies based on the representative dataset (Fig. 1a–d and Supplementary Figs S1–S16) were surprisingly well-resolved (Supplementary Table S3).The parameter set 1.A maximizes congruence between data partitions, providing the shortest supertree with the highest CI and RI values (Supplementary Table S3). The resulting supertree topologies are, overall, robust to parameter set changes. Similarity of the resulting supertrees measured by subtree prune and regraft (SPR) distances is 99–74% (Supplementary Table S4a,b). The contribution of admixture plots to the resulting supertree topology was relatively small. The topology of the combined supertree based on parameter set 1.A, where the effect of admixture plots was maximized, was 85% similar to the parameter set 2.B where the effect of admixture plots was minimized while all other data partitions played an equal role (Supplementary Table S4a,b; Supplementary Figs S1 and S6). The admixture plots alone provided a more symmetrical topology with three superclades: the basal African, followed by the N African–W Eurasian, and the Eastern superclade (Supplementary Fig. S19). However, the hierarchical clustering of populations in admixture plots is not always comparable to the order of branching events in human population history. The early divergence of some populations (e.g., Hadza, Dogon, Basque, and Tibetan) implied by the admixture plots could reflect isolation and random genetic drift rather than early divergence. Some relationships probably reflect relatively recent admixture (e.g., Bantu populations of S Africa^4^).

In all 16 topologies provided by the representative dataset (Fig. 1 and Supplementary Figs S1–S16) sub-Saharan Africa is located nearest to the root of the tree, followed by N Africa, the Near East, Europe, S and C Asia, Oceania, E Asia and America. The general branching order is largely consistent with the previously published global human population-level phylogenies, despite major differences in sampling and phylogenetic inference methods used^3^,^4^,^12^,^31^. All 16 topologies based on representative dataset agreed upon the most basal position of S African Khoisan followed by C African Pygmies, and the clade consisting of Fulani Afro-Asiatic (Cushitic) speaking populations as a sister group to Niger-Congo speaking populations (including Bantu). The next paraphyletic section of the supertree included Niger-Congo (Bantu), Nilo-Saharan, and Afro-Asiatic (Cushitic, Omotic, and Semitic) peoples and the click-speaking Hadza and Sandawe hunter-gatherers of E Africa, The clustering of Chadic speaking populations of C Africa with Niger-Congo speaking populations of this region rather than with Afro-Asiatic speaking populations of E Africa was consistent with the previously published genomic trees^3^,^4^. So was the position of Hadza and Sandawe within the ethno-linguistically heterogeneous E African section of the supertree^4^,^12^. This section was basal to the monophyletic clade including N African and Eurasian peoples. The latter consisted of the largely unresolved N African–W Eurasian assemblage (N African, Near Eastern, European, and S Asian peoples) and the consistently monophyletic Eastern superclade (Sahul–Oceanian, E Asian, and Beringian–American peoples). The most remarkable differences between individual topologies derived from different parameter sets concerned W Eurasia, Mainland and Island SE Asia and Oceania, and E Asia. In the N African–W Eurasian assemblage, there are highly unstable relationships among its constituent sections (N Africa, Near East, Europe, and S Asia), most of which tend to be para- or even polyphyletic (Fig. 1a,b). In Mainland and Island SE Asia and Oceania, different parameter sets imply a different source of the expansion into the area (either from Taiwan or from Malay Peninsula) and a varying degree of admixture of Austronesians with Sahul–Melanesian peoples (Fig. 1a,c). In E Asia, there is an unstable relationship between populations of E and SE Asia and a highly unstable position of some Siberian peoples (Evenki, Ket, Yukaghir) who were either recovered at the basal position within the E Asian clade or within the Beringian–American clade (Fig. 1d).

**Figure 2:**
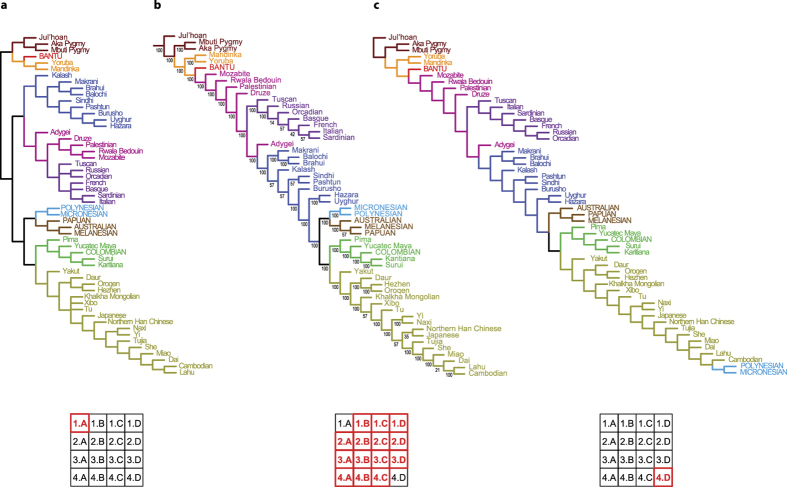
(**a**) Semistrict consensus supertree of 55 human populations based on HGDP dataset and parameter set 1.A of the sensitivity analysis. Populations were renamed to correspond to those used in the HGDP panel. BANTU = Kikuyu; POLYNESIAN = Samoan + Maori; MICRONESIAN = Kosraean; MELANESIAN = Naasioi; PAPUAN = Goroka; COLOMBIAN = Piapoco + Curripaco. The color code corresponds to Fig. 1. (**b**) Frequency-differences consensus of 14 supertrees based on parameter sets 1.B–4.C of the sensitivity analysis. (**c**) Semistrict consensus supertree based on parameter set 4.D of the sensitivity analysis. The geographic color code corresponds to Fig. 1.

Sensitivity analysis of the HGDP dataset produced three distinct topologies (Fig. 2). They were, for the most part, congruent with the supertrees based on the representative dataset, although they included a few clades that were not recovered in the representative-dataset supertrees. The topology recovered under parameter set 1.A (Fig. 2a), was the most symmetrical and included monophyletic superclades as follows: sub-Saharan African (with Khoisan–Pygmy and Bantu–E African subclades), N African–W Eurasian (with S Asian, N African–Near Eastern and European subclades), and Eastern (with Sahul–Oceanian, American, and E Asian subclades). The topologies recovered under parameter sets 2.A–4.C (Fig. 2b) were fully compatible with the representative-dataset supertrees, and in agreement with other studies using similar population samples^3^,^31^, regardless of the tree-building techniques used. In the topology recovered under parameter set 4.D (Fig. 2c), the “Oceania” clade situated in the base of the Eastern superclade in most supertrees, was recovered as polyphyletic. The Sahul–Melanesian subclade remained basal to the rest of the Eastern superclade, while the Micronesian–Polynesian (“Remote Oceanian”) subclade was deeply nested within the E Asian populations.

The most important point of conflict among the alternative supertree topologies thus concerned the position of Sahul–Melanesian and Micronesian–Polynesian peoples. Phylogenetic affinities of Sahul–Melanesian peoples varied greatly between the source trees. While in multiple studies, Sahul–Melanesia was placed basally, often as a sister-group to E Eurasia as a whole^3^,^4^,^12^, in others they were nested deeply within SE Asia^31^,^32^,^33^. These topological conflicts reflect the complex population history of Island SE Asia, from early “out-of-Africa” migration via the “southern route”^34^ through later interactions with Mainland SE Asia^9^,^35^ up to the putative “express-train” migration of the Austronesian speakers from Taiwan via the Philippines, Greater and Lesser Sunda Islands, and Melanesia to Micronesia and Polynesia^36^,^37^,^38^. The phylogenetic placement of Sahul–Melanesia is further complicated by possible gene flow from India to Australia around the mid-Holocene^39^.

The supertree topology is notably pectinante in agreement with the previously published global human population-level phylogenies^3^,^12^,^31^. There were just a few apparent major radiations, namely, Bantu and related sub-Saharan populations (Fig. 1a), European or W Eurasian (Fig. 1a,b), SE Asian–Oceanian (with or without the Sahul–Melanesian peoples) (Fig. 1a,c), E Asian (Fig. 1a,d), and Beringian–American. Individual small clades or even individual terminal taxa tended to branch off from the major migration route in E Africa, Near East, and S Asia. This topology is consistent with a serial founder effect model, which suggested that human populations have remained in the locations they first colonized after the out-of-Africa expansion, exchanging migrants only at a low rate with their immediate neighbors, until the long-range migrations began to happen.

### Wildcard taxa

Twenty-four populations, either terminal taxa or small clades, were identified as wildcards in topologies recovered under one or more parameter sets of the sensitivity analysis (Supplementary Table S5). The populations responsible for the greatest loss of resolution (5 nodes or more) throughout the sensitivity analyses were Andamanese (a wildcard taxon in 14 parameter sets, decreasing resolution by 1–21 nodes; see below), Malagasy (12: 3–23; see below), Dayak Ngaju (2: 1 and 10; identified as either Island or Mainland SE Asians), and Qatari (2: 21; highly unstable position within N African–W Eurasian section of the supertree).

The unstable position of some populations provides clues about conflicts within the dataset, which reflects either the paucity of data or complex population history of the peoples in question. For example, the unstable position of Malagasy reflects a relatively recent (ca. 1,200 ya) migration of Austronesian-speaking people across the Indian Ocean, followed by admixture with E Africans. While linguistic evidence places Malagasy language within Barito group of W Malayo-Polynesian (Austronesian) languages^36^, Malagasy population exhibit genetic affinities to both SE Asian and E African populations^40^.

The case of Andaman Islanders is much more complicated. They were recovered either as Sahul–Melanesians or S Asians, or at the base of E Asia under different parameter sets (Supplementary Figs S17 and S18). Position of Andamanese within the Sahul–Melanesian clade is based on the analysis of structural features of language using a Bayesian clustering algorithm^38^. Initial genetic studies suggested that Andamanese are descendants of an early “out-of-Africa” migration^41^, while later studies proposed a more recent S or E Indian origin^42^. Recent studies agree that Andamanese represent an isolated, relatively basal lineage, with possible genetic affinities to both Sahul–Melanesia and S Asia^33^,^39^. The relatedness of Andamanese to Sahul–Melanesians, particularly the Papuans, has recently been substantiated also by genomic data^43^,^44^.

### Assessment of gene–language coevolution

The question of coevolution of genes and languages is considered fundamental but rarely studied by formal phylogenetic methods. Although the genetic and linguistic evolution may often be correlated, the assumption of direct coevolution between genes and languages is evidently misleading^45^. Evolutionary processes shaping genetic diversity are not directly analogous to those shaping linguistic diversity^46^ and, consequently, genetic and linguistic data often imply different historical scenarios^47^.

The supertree dataset included 45 linguistic source trees and one linguistic admixture plot from 34 studies (compared to 213 genetic/genomic source trees and 43 genomic admixture plots from 170 studies). The 535 (~9%) parsimoniously informative characters based on these sources contributed only marginally to the resulting supertree topology (Supplementary Fig. 21). In fact, only a few language families have so far been analyzed phylogenetically, and hence numerous areas of the supertree included no linguistic data at all. Only a few small clades were supported by linguistic characters. The supertree topology was, in general, dominated by the genetic/genomic data.

In order to test for monophyly of the proposed linguistic macrofamilies we created two datasets based on formal linguistic classifications (Supplementary Table S6) to be both optimized on, and to constrain, the topology of the supertree based on the representative dataset. The first dataset was based on linguistic classification in *Ethnologue*^48^ on the level of language families (“*Ethnologue*” hereinafter). The second dataset (“Greenberg–Ruhlen” hereinafter) included additional characters based on linguistic classification by Ruhlen^49^ on the level of linguistic macrofamilies, and by Greenberg & Ruhlen^50^ on the level of linguistic stocks within the Amerind macrofamily. The hunter–gatherer populations, who speak the languages of neighboring agriculturalist or pastoralist groups as a result of a relatively recent language shift (C African Pygmies, “Negritos” of Malaysia and Philippines) were not scored for linguistic characters (see Methods).

Optimization of the datasets based on linguistic classification on the representative-dataset supertree showed rather poor fit of the classification on the supertree topology (*Ethnologue* and Greenberg–Ruhlen datasets’ CI’s were 0.27 and 0.25, respectively, for the purely genetic, and 0.31 and 0.28, respectively, for the combined supertree). Within *Ethnologue* dataset, the best fitting language families were Austronesian, S African Khoisan, Afro-Asiatic (especially Semitic languages), and Indo-European (Supplementary Table S7a). Within Greenberg–Ruhlen dataset, the macrofamily which is by far the most consistent with the supertree topology is Amerind, followed by Austric (and its constituent language families Austronesian and Austroasiatic), Afro-Asiatic, Khoisan, and Indo-Hittite. We do not consider the good fit of Amerind on the supertree topology as support for the Amerind hypothesis^21^, but rather a consequence of geographic and genetic coherence of the presumably Amerind-speaking populations. The linguistic stocks within the Amerind macrofamily are not consistent with the supertree topology (Supplementary Table S7b). The other controversial linguistic macrofamilies, such as Eurasiatic/Nostratic, Macro-Altaic, Dene–Yeniseian, and Dene–Caucasian, fitted poorly on the supertree topology. The poor fit of Macro-Altaic and the families that constitutes it (especially the Turkic) is in agreement with the fact that there is only a weak unifying genetic signal for the Turkic-speaking populations across Eurasia^51^. The expansion of Turkic languages has probably been largely mediated by language replacements rather than demic expansion.

**Figure 3:**
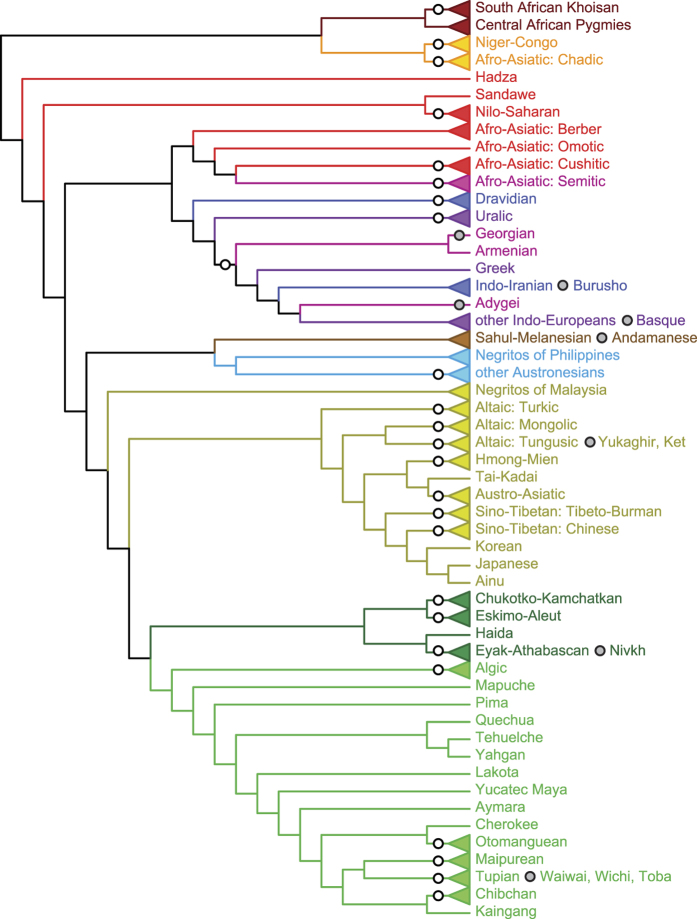
The supertree constrained by *Ethnologue* classification. White circles indicate topological constraints. Grey circles indicate an unconstrained taxon or clade (usually a language isolate) recovered within a constrained one. The geographic color code corresponds to Fig. 1.

The supertrees constrained by *Ethnologue* (Fig. 3) and Greenberg–Ruhlen datasets (Supplementary Fig. S22) summarized relationships between groups of populations speaking related languages based on genetic data (congruence between the purely genetic supertree and the supertree constrained by the Greenberg–Ruhlen dataset is illustrated by a tanglegram and by “anticonsensus” trees; Supplementary Figs S22–S24). The *Ethnologue* dataset included only those language families that are relatively non-controversial, and the supertree constrained by *Ethnologue* classification (Fig. 3) is in many respects similar to the combined supertree, although it is more symmetrical. Importantly, it includes several monophyletic clades that are not based on the linguistic topological constraint used. In sub-Saharan Africa, there is a clade including peoples speaking S African Khoisan, Niger-Congo, and Chadic languages. There is also a large clade including peoples speaking Afro-Asiatic (excl. Chadic), Dravidian, Uralic, and Indo-European languages, a group roughly coextensive with the hypothesized Eurasiatic/Nostratic macrofamilies^17^,^18^,^19^; however, the Altaic, Chukotko-Kamchatkan and Eskimo-Aleut-speaking peoples (that have been hypothesized to belong to the Eurasiatic/Nostratic macrofamily as well) are not closely related genetically. The relationships between individual language families of the hypothesized Eurasiatic macrofamily^17^,^18^ (i.e., Dravidian, Kartvelian, Indo-European, Uralic, Altaic, Chukchi-Kamchatkan and Eskimo-Aleut) are largely consistent with those inferred in by Pagel *et al*.^15^. There is, however, no support for monophyly of the hypothesized Eurasiatic languages as a whole (Supplementary Table S7c and Supplementary Fig. S22a). Another large clade includes Altaic, Austric (excl. Austronesian), and Sino-Tibetan languages, together with Korean–Japanese and Ainu. Whereas Ainu was considered related either to Eurasiatic^17^,^18^,^49^ or Austric^16^ macrofamily, Korean and Japanese were seen as distant relatives of Altaic languages^49^. On the contrary, the close relationships between Korean–Japanese–Ainu and Sino-Tibetan peoples have no linguistic basis. The clade which includes Australian, Papuan, Melanesian, and Andamanese populations is somewhat reminiscent of the controversial Indo-Pacific macrofamily^20^. The Na-Dene, Eskimo–Aleut, and Chukotko-Kamchatkan populations are closely related to Amerind, a connection that has no linguistic basis. Within America, there is a conspicuous basal placement of populations of the Southern Cone (Andean languages according to Greenbeg–Ruhlen; Supplementary Fig. S22b), which could be indicative of an early western route used during the initial colonization of Americas.

The hunter-gatherer groups that were deliberately not scored for linguistic characters were recovered outside of the clades they belong to based on their linguistic affiliation. C African Pygmies were recovered as a sister group to S African Khoisan (or within the Khoisan family in the supertree constrained by the Greenberg–Ruhlen classification; Supplementary Fig. S22a), providing evidence for shared ancestry among these geographically diverse groups of hunter-gatherers^4^. E African Hadza (but not Sandawe) were recovered at a basal position within Africa just above the S African Khoisans and C African Pygmies. “Negritos” of Malaysia were placed as a sister group to the whole Eastern superclade, and “Negritos” of Philippines were recovered as a sister group to Austronesian language family (or within the clade including languages of Australia and Indo-Pacific languages in the supertree constrained by the Greenberg–Ruhlen classification; Supplementary Fig. S22a), providing evidence for the hypothesis that the “Negrito” populations represent the descendants of the early migration into the area^34^, with lasting genetic affinities to Sahul–Melanesia^39^,^43^,^44^.

Interestingly, when the supertree topology was constrained to include linguistic-compatible clades, as if the language families were indeed monophyletic, it tended to form more inclusive clades, which are more or less compatible with the proposed linguistic macrofamilies (Eurasiatic/Nostratic, Indo-Pacific, and especially Amerind). It is possible that the “macrofamilies” are consistent genetically and geographically rather than linguistically; however, the possibility that historical linguistics is able to reconstruct the most basal relationships between modern human populations should be re-assessed critically^6^.

The supertree can provide a robust framework for studies concerning evolution of culture^52^. Such a framework is needed because most cross-cultural comparative studies published to date used language phylogenies^5^,^7^. Although language phylogenies provide an excellent proxy for population histories in some regions (e.g., Remote Oceania), this is not universally the case^45^,^47^. Linguistic data seems to be unable to provide a global tree of human populations due to a limited timescale over which linguistic inference is possible^6^. On the other hand, genetic phylogenies, although global, could be unsuitable for studies of cultural evolution, as the population history they inform of can be older than the cultural traits under investigation. A time-calibrated supertree, incorporating all time “strata” of human evolution (and informed by ancient DNA), is needed to elevate the studies of cultural evolution to a global level.

## Methods

### Data

Source trees published in peer-reviewed journals, edited volumes and monographs between 1990 and 2013 (most of them post 2007) were collected (including papers “in press” by the end of 2013). Altogether 257 source trees (obtained by using both distance-based and character-based methods) and 44 admixture plots from 200 published studies contributed to the present dataset. Only trees that were inferred by formal phylogenetic methods and based on original analyses were utilized. The protocol for inclusion and rejection of source trees was guided by the issues of sufficient taxonomic coverage and data quality (see Supplementary Methods). In order to ameliorate the problem of data non-independence and duplication, we used a protocol for source-trees retention and exclusion proposed by Bronzati *et al*.^53^ (see Supplementary Methods). In addition to the trees, we also utilized admixture plots, produced by software like STRUCTURE, FRAPPE, and ADMIXTURE, as a source of hierarchical information for supertree construction (see the section “Matrix representation with parsimony” and Supplementary Methods).

### Taxonomic nomenclature and taxonomic level

To synthesize published phylogenies from different sources, the names of terminal taxa from the source trees were standardized using ISO 639-3 codes from *Ethnologue*^48^, a standard, widely recognized taxonomic reference. Information on geographic range of a population in question, sampling location(s) of genotyped individual(s), linguistic affiliation and ethnonyms were utilized in order to standardize the taxonomy among individual sources. Where higher-level taxa (e.g., population or linguistic groupings above the level of ethno-linguistic groups listed in *Ethnologue*) were used in the source studies, they were either replaced by a single population based on information from the original study, or, when this information was insufficient or unavailable, by “type” population(s) (Supplementary Table S1). Lower-level taxa (e.g., local populations or language dialects) took on the names of the corresponding ethno-linguistic groups listed in *Ethnologue* (see Supplementary Methods). Populations of well-known recent mixed ancestry (e.g., “African American”, “US Hispanic”, “Cape Mixed Ancestry”), colonial populations (e.g., Boer), creole languages (e.g., Haitian), and loosely specified higher level taxa (e.g., “African”, “Native North American”) were not included. The only exceptions were Australian Aboriginals of unspecified ethnic population origin that were merged together and analyzed as a single terminal taxon, named “AUSTRALIAN” (see Supplementary methods).

### Population samples

Two datasets were created. The first dataset of 186 populations included all world regions and major linguistic groups that were reasonably well represented throughout the source trees (“representative dataset”) (Supplementary Table S2). The representative dataset included 5,987 parsimoniously informative characters. The second dataset consisted of 51 populations from the Human Genome Diversity Project (HGDP) panel^3^, plus three additional populations to represent Australia, Micronesia and Polynesia (“HGDP dataset”). The HGDP dataset included 3,070 parsimoniously informative characters (see Supplementary Methods).

### Matrix representation with parsimony

The matrix representation with parsimony (MRP) method^24^,^25^ is based on creating, merging and reanalyzing matrix representations of the source trees: each source tree was converted into a partial matrix of additive binary characters. Taxa descended from a given node were coded as “1” (=present); those that did not were coded as “0” (=absent); all taxa that were not present in the given source trees were coded as “–” (=inapplicable). Each admixture plot was converted into a matrix representation such that each population was coded as “1” (=present) or as “0” (=absent) based on the proportions of individual genotypes attributable to each cluster. Limited attribution to a given cluster (less than 10%) was neglected, and ambiguous sections of a plot (borderline proportions or different proportions in individuals within a single population) were coded as “?” (=unknown). The resulting matrix of additive binary characters was analyzed by the MP method to obtain a tree which corresponds to clustering implied by the admixture plot. The trees based on admixture plots typically contain unresolved sections due to membership of some populations in several clusters, but they still preserve enough valuable branching information. The merged character matrix consisting of matrix representations of trees and admixture plots was analyzed by the MP method to obtain a supertree presented in the form of a strict or semistrict consensus tree.

### Phylogenetic analysis

Phylogenetic analyses were performed in TNT ver. 1.1^54^ under “new technology search” with search level 10 using sectorial, ratchet, and tree fusing searches, obtaining trees from a 10,000-replicate random addition sequence, followed by additional branch swapping using the tree-bisection and reconnection method (see Supplementary Methods). The datasets were analyzed without any topological constraints (i.e., without any assumptions on geographic regions or language families).

### Sensitivity analysis and wildcard taxa identification

To investigate robustness of the inferred supertree topology, we used a method inspired by Wheeler’s “sensitivity analysis”^27^ (see Supplementary Methods). We used 16 parameter sets (for each population sample in parallel), based on combinations of four weighting and four rooting schemes as follows: either (1) all data partitions were weighted equally, or (2) all trees were upweighted by the factor of 1,000 relative to admixture plots, or (3) genetic/genomic trees were upweighted by the factor of 1,000 relative to language trees and all admixture plots, or (4) genomic trees were upweighted by the factor of 1,000 relative to all remaining data partitions; and either (A) all rooted source trees and admixture plots were treated as rooted (by inserting a hypothetical “all-0” outgroup), or (B) only rooted source trees were treated as rooted, or (C) only rooted genetic/genomic trees were treated as rooted, or (D) only source trees/admixture plots featuring great ape and/or archaic hominin outgroups (*Gorilla gorilla*, *Pan paniscus*, *P. troglodytes*, Denisova hominin, *Homo neanderthalensis*) were treated as rooted. When performing sensitivity analysis on the HGDP dataset, the data partitions were either downweighted as above (1:1,000) or completely deactivated to test whether the weighting scheme was sufficient to minimize the effect of a data partition on the resulting topology. Sixteen sets of the most parsimonious trees, recovered in the sensitivity analysis of the representative dataset, were analyzed using *Iter*PCR script^28^ as implemented in TNT^29^, to improve resolution of the consensus tree by identifying taxa of unstable positions (“wildcard taxa”^30^). Alternative positions of the identified wildcards were investigated using pruned strict consensus (*nelsen//*) in TNT. Four wildcards that decreased resolution of the supertree by five or more nodes were excluded from the dataset, and the pruned version of the representative dataset (182 populations) was used for subsequent analyses (Supplementary Methods).

### Linguistic constraints

In order to infer the relationships of language families and macrofamilies, we created two datasets based on linguistic classification. The first dataset included 37 parsimoniously informative characters based on *Ethnologue* classification^48^ on the level of language families. The second dataset included additional 26 parsimoniously informative characters based on on classification by Ruhlen^49^ and Greenberg & Ruhlen^50^ on the level of linguistic macrofamilies and linguistic stocks within the Amerind macrofamily; Supplementary Table S6; Supplementary Methods). Characters based on *Ethnologue*^48^, Ruhlen^49^ and Greenberg & Ruhlen^50^ were fully congruent, with no hard conflict between them. Hunter–gatherer populations, speaking languages of neighboring groups, were scored as “unknown” (“?”) in both datasets. These included C African Pygmies (Mbuti Pygmy, Aka Pygmy) who speak Niger-Congo or Nilo-Saharan languages^55^, the “Negritos” of Malaysia (Jehai and Kensiu) who speak Austro-Asiatic languages^56^,^57^, and the “Negritos” of Philippines (Agta, Aeta, and Mamanwa) who speak Malayo-Polynesian (Austronesian) languages^58^. Similarly, Ashkenazi Jews who used to speak Indo-European (Germanic) Yiddish were not scored for linguistic characters. (Supplementary Methods). Language-constrained supertrees (Fig. 3; Supplementary Fig. S22a,b) were inferred by analyzing all data partitions rooted by “all-0” outgroup together with the *Ethnologue* and Greenberg–Ruhlen datasets; the data partitions based on linguistic sources and the datasets based on linguistic classification were upweighted by the factor of 1,000 relative to genetic data partitions.

### Supertrees comparison and phylogenetic signal

The resulting supertree topologies were compared using the SPR distance measure (*sprdiff*) and the “anticonsensus” measure (*tcomp*) in TNT software. Topology of the supertree constrained by the Greenberg–Ruhlen classification was compared with the purely genetic supertree using a tanglegram computed in Dendroscope ver. 3.2.10^59^. In order to assess the support for proposed linguistic groupings (macrofamilies, stocks, and families), consistency index (CI) and retention index (RI) values were calculated in Mesquite ver. 3.02^60^ for each character in the linguistic classification datasets optimized onto the purely genetic and combined supertree topologies (based on parameter set 1.A, see the section “Sensitivity analysis and wildcard taxa identification”). The resulting CI values were compared to the minimum possible CI values (for a binary character, CI_min_ = 1/N, where N taxa were scored positively for presence of a character), which made the values directly comparable for language families represented by different number of populations (Supplementary Methods).

### Plotting

Plotting of sampling locations on the world map was performed using an open source software QGIS v.2.8 (http://qgis.org/en/site/) with open-source map.

## Additional Information

**How to cite this article**: Duda, P. *et al*. Human population history revealed by a supertree approach. *Sci. Rep.* **6**, 29890; doi: 10.1038/srep29890 (2016).

## Supplementary Material

Supplementary Information

Supplementary methods
