# Supplementary material for: Human population history revealed by a supertree approach
Source: Sci Rep. 2016 Jul 19;6:29890. doi: 10.1038/srep29890 (PMC4949479; doi:10.1038/srep29890)
Supplement: Supplementary Information [file srep29890-s1.pdf]

# Human population history revealed by a supertree approach

**Pavel Duda & Jan Zrzavý**

Department of Zoology, Faculty of Science, University of South Bohemia, České Budějovice, Czech Republic

Correspondence and request for material should be addressed to P. D. (email: dudapa01@.gmail.com)

# SUPPLEMENTARY INFORMATION

Includes Supplementary Tables S1-S8, Supplementary Figures S1-S24 and Supplementary Methods.

## SUPPLEMENTARY TABLES

**Supplementary Table S1.** Type populations used to replace populations groups, subpopulations, and populations of unspecified ethnic origin in the source trees.

| Taxon name(s) (source trees) | References | Taxon name (supertree) | ISO 639-3 | Comment |
|---|---|---|---|---|
| !Kung; Bushmen | 1-7 | !Kung | vaj, knw | |
| African | 8 | Yoruba | yri | Individuals from Nigeria |
| Africans; South African; South African Blacks | 9-11 | Xhosa | xho | Individuals from Johanesburg, South Africa |
| | | Zulu | zul | |
| Algonquian | 12 | Ojibwa | ojc, ojg, ojb, ojs | |
| | | Cree | cre | |
| Andamanese; Andaman Islanders | 1,13 | Andamanese | oon, anq, gac | |
| Ashkenazi Jews; Polish Jews and Russian Jews | 14-19 | Ashkenazi Jew | ydd, yih | |
| Australian aboriginals | various studies | AUSTRALIAN | | |
| Central Australian Aboriginals | 20 | Warlpiri | wbp | |
| CEU; European(s); European (Northwestern); European-American; U.S.; American White; US Caucasian; N.American Caucasoids; N.A. Caucasoids; Caiucasoids; Canada; Canadians | various genome-wide SNP- and STR-based studies | English | eng | The CEU sample[21] shows ancestry most similar to British in England and Scotland[22] |
| Ethiopian | 23 | Amhara<br>Oromo | amh<br>gax | |
| Great Andamanese | 24,25 | Andamanese | oon, anq, gac | |
| Guarani | various HLA-based studies | Guarani | grn | |
| Han Chinese from Beijing, Shanghai, Shandong, and Wuxi | various studies | Northern Han Chinese | cmn | |
| Han Chinese from Hongkong, Fujian, Chaosan, Huizhou, Hainan, Guangdong, Guangxi, Yunnan, Guizhou, Huangzhou (Han Cantonese), and Xiaamen | | Southern Han Chinese | cmn, yue | |
| Han Chinese from Singapore | various studies | Singapore Chinese | cmn | |
| HGDP Papuan; PNG highland; PNG Highlanders; New Guinea Highlands | various studies | Goroka | for, gim | |
| Chinese from Taiwan | various studies | Min Nan Taiwanese | nan | |
| Chinese; Taiwan(ese); Taiwan Han; China Taiwan | 2,3,26-33 | Min Nan Taiwanese | nan | |

| | | | | |
|---|---|---|---|---|
| Jarawa | 24 | Andamanese | oon, anq, gac | |
| Lapp(s); Laps; Eur. Saam(i); Saami (Lapps); Swedish Lapp; Norwegian Lapp; Finnish Lapp; Skolt Saami; North Saami; Ume Saami; Sweden South Sami; Sweden North Sami; Saami Russia | 1,11,20,23,27,34-45 | Saami | sia, smn, sjd, sms, sjt | |
| Madagascar | 46,47 | Malagasy | plt | |
| Micronesia(n) | 48; various genome-wide SNP- and STR-based studies | Kosraean | kos | |
| NAN Melanesian; HGDP Melanesian; Melanesian; Southeast Bougainville | 23; various genome-wide SNP- and STR-based studies | Naasioi | nas | |
| Nigeria | 4,49 | Yoruba | yor | |
| Non-Ashkenazi Jews from Israel; Syria; Iraq; Iran; Turkey; Georgia and Azerbaijan; Hebrew | 16,17; various HLA-based studies | Mizrahi Jew | | |
| North Dravidian | 23 | Brahui | brh | |
| North European | 50,51 | German | deu | |
| Onge | 25,52,53 | Andamanese | oon, anq, gac | |
| Polynesia(n) | 48; various genome-wide SNP- and STR-based studies | Samoan | smo | |
| Romania; Romanian(s); Rumanian; Romanian List; Vlach | 17,30,54-70 | Romanian | ron, rup | |
| South Dravidian | 25,71 | Tamil | tam | |
| South Indian | 17,23,25,36,72 | Malayalam | mal | |
| Taiwan; Taiwan Aborigine(s); Taiwanese; Indigenous Taiwanese | 36,49,73-76 | Atayal | tay | |
| Uralic Siberian | 23 | Nenets | yrk | |

## References


1 Sellen, D. W. & Mace, R. Fertility and Mode of Subsistence: A Phylogenetic Analysis. *Current Anthropology* **38**, 878-889, doi:10.1086/204677 (1997).

2 Comas, D. *et al.* Trading genes along the silk road: mtDNA sequences and the origin of central Asian populations. *American Journal of Human Genetics* **63**, 1824-1838, doi:10.1086/302133 (1998).

3 Chu, C. C. *et al.* Diversity of HLA among Taiwan's indigenous tribes and the Ivatans in the Philippines. *Tissue Antigens* **58**, 9-18, doi:10.1034/j.1399-0039.2001.580102.x (2001).

4 Nasidze, I. *et al.* Alu insertion polymorphisms and the genetic structure of human populations from the Caucasus. *European Journal of Human Genetics* **9**, 267-272, doi:10.1038/sj.ejhg.5200615 (2001).

5 Xing, J. C. *et al.* Fine-scaled human genetic structure revealed by SNP microarrays. *Genome Research* **19**, 815-825, doi:10.1101/gr.085589.108 (2009).

6 Alkorta-Aranburu, G. *et al.* The Genetic Architecture of Adaptations to High Altitude in Ethiopia. *Plos Genetics* **8**, doi:10.1371/journal.pgen.1003110 (2012).

7 Wangkumhang, P. *et al.* Insight into the Peopling of Mainland Southeast Asia from Thai Population Genetic Structure. *PloS one* **8**, e79522 (2013).

8 Yuasa, I. *et al.* A hypervariable STR polymorphism in the CFI gene: Southern origin of East Asian-specific group H alleles. *Legal Medicine* **15**, 239-243, doi:10.1016/j.legalmed.2013.04.001 (2013).

9 Bannai, M. *et al.* Analysis of HLA genes and haplotypes in Ainu (from Hokkaido, northern Japan) supports the premise that they descent from Upper Paleolithic populations of East Asia. *Tissue Antigens* **55**, 128-139, doi:10.1034/j.1399-0039.2000.550204.x (2000).

10 Arnaiz-Villena, A. *et al.* HLA alleles and haplotypes in the Turkish population: relatedness to Kurds, Armenians and other Mediterraneans. *Tissue Antigens* **57**, 308-317, doi:10.1034/j.1399-0039.2001.057004308.x (2001).

11 Martinez-Laso, J. *et al.* HLA in Jaidukama: an Amerindian secluded Colombian population with new haplotypes and Asian and Pacific-shared alleles. *Molecular Biology Reports* **38**, 3689-3701, doi:10.1007/s11033-010-0483-6 (2011).

12 Rubicz, R. *et al.* Genetic Structure of Native Circumpolar Populations Based on Autosomal, Mitochondrial, and Y Chromosome DNA Markers. *American Journal of Physical Anthropology* **143**, 62-74, doi:10.1002/ajpa.21290 (2010).

13 Walker, R. S., Hill, K. R., Flinn, M. V. & Ellsworth, R. M. Evolutionary History of Hunter-Gatherer Marriage Practices. *Plos One* **6**, doi:10.1371/journal.pone.0019066 (2011).

14 Kopelman, N. M. *et al.* Genomic microsatellites identify shared Jewish ancestry intermediate between Middle Eastern and European populations. *Bmc Genetics* **10**, doi:10.1186/1471-2156-10-80 (2009).

15 Li, H., Cho, K., Kidd, J. R. & Kidd, K. K. Genetic Landscape of Eurasia and "Admixture" in Uyghurs. *American Journal of Human Genetics* **85**, 934-937, doi:10.1016/j.ajhg.2009.10.024 (2009).

16 Atzmon, G. *et al.* Abraham's Children in the Genome Era: Major Jewish Diaspora Populations Comprise Distinct Genetic Clusters with Shared Middle Eastern Ancestry. *American Journal of Human Genetics* **86**, 850-859, doi:10.1016/j.ajhg.2010.04.015 (2010).

17 Behar, D. M. *et al.* The genome-wide structure of the Jewish people. *Nature* **466**, 238-U112, doi:10.1038/nature09103 (2010).

18 Haber, M. *et al.* Genome-Wide Diversity in the Levant Reveals Recent Structuring by Culture. *Plos Genetics* **9**, 8, doi:10.1371/journal.pgen.1003316 (2013).

19 Pemberton, T. J., DeGiorgio, M. & Rosenberg, N. A. Population Structure in a Comprehensive Genomic Data Set on Human Microsatellite Variation. *G3-Genes Genomes Genetics* **3**, 891-907, doi:10.1534/g3.113.005728 (2013).

20 Nei, M. & Roychoudhury, A. K. Evolutionary relationships of human populations on a global scale. *Molecular Biology and Evolution* **10**, 927-943 (1993).

21 Gibbs, R. A. *et al.* The International HapMap Project. *Nature* **426**, 789-796, doi:10.1038/nature02168 (2003).

22 Shriner, D., Tekola-Ayele, F., Adeyemo, A. & Rotimi, C. N. Genome-wide genotype and sequence-based reconstruction of the 140,000 year history of modern human ancestry. *Scientific Reports* **4**, doi:10.1038/srep06055 (2014).

23 Cavalli-Sforza, L. L., Menozzi, P. & Piazza, A. *The history and geography of human genes*. (Princeton university press, 1994).

24 Abbi, A. Is Great Andamanese genealogically and typologically distinct from Onge and Jarawa? *Language Sciences* **31**, 791-812, doi:10.1016/j.langsci.2008.02.002 (2009).

25 Pugach, I., Delfin, F., Gunnarsdottir, E., Kayser, M. & Stoneking, M. Genome-wide data substantiate Holocene gene flow from India to Australia. *Proceedings of the National Academy of Sciences of the United States of America* **110**, 1803-1808, doi:10.1073/pnas.1211927110 (2013).

26 Horai, S. *et al.* mtDNA polymorphism in East Asian populations, with special reference to the peopling of Japan. *American Journal of Human Genetics* **59**, 579-590 (1996).

27 Wells, R. S. *et al.* The Eurasian Heartland: A continental perspective on Y-chromosome diversity. *Proceedings of the National Academy of Sciences of the United States of America* **98**, 10244-10249, doi:10.1073/pnas.171305098 (2001).

28 Tajima, A. *et al.* Mitochondrial DNA polymorphisms in nine aboriginal groups of Taiwan: implications for the population history of aboriginal Taiwanese. *Human Genetics* **113**, 24-33, doi:10.1007/s00439-003-0945-1 (2003).

29 Tajima, A. *et al.* Genetic origins of the Ainu inferred from combined DNA analyses of maternal and paternal lineages. *Journal of Human Genetics* **49**, 187-193, doi:10.1007/s10038-004-0131-x (2004).

30 Nowak, J. *et al.* Allele and extended haplotype polymorphism of HLA-A, -C, -B, -DRB1 and -DQB1 loci in Polish population and genetic affinities to other populations. *Tissue Antigens* **71**, 193-205, doi:10.1111/j.1399-0039.2007.00991.x (2008).

31 Alfonso-Sanchez, M. A., Perez-Miranda, A. M. & Herrera, R. J. Autosomal microsatellite variability of the Arrernte people of Australia. *American Journal of Human Biology* **20**, 91-99, doi:10.1002/ajhb.20685 (2008).

32 Zhu, B. F. *et al.* Distributions of HLA-A and -B alleles and haplotypes in the Yi ethnic minority of Yunnan, China: relationship to other populations. *Journal of Zhejiang University-Science B* **11**, 127-135, doi:10.1631/jzus.B0900232 (2010).

33 Yuliwulandari, R. *et al.* Polymorphisms of HLA genes in Western Javanese (Indonesia): close affinities to Southeast Asian populations. *Tissue Antigens* **73**, 46-53, doi:10.1111/j.1399-0039.2008.01178.x (2009).

34 Holden, C. & Mace, R. Phylogenetic analysis of the evolution of lactose digestion in adults. *Human Biology* **69**, 605-628 (1997).
35 Ingman, M., Kaessmann, H., Paabo, S. & Gyllensten, U. Mitochondrial genome variation and the origin of modern humans. *Nature* **408**, 708-713 (2000).
36 Ingman, M. & Gyllensten, U. Mitochondrial genome variation and evolutionary history of Australian and New Guinean aborigines. *Genome Research* **13**, 1600-1606, doi:10.1101/gr.686603 (2003).
37 Nasidze, I., Sarkisian, T., Kerimov, A. & Stoneking, M. Testing hypotheses of language replacement in the Caucasus: evidence from the Y-chromosome. *Human Genetics* **112**, 255-261, doi:10.1007/s00439-002-0874-4 (2003).
38 Agrawal, S., Srivastava, S. K., Borkar, M. & Chaudhuri, T. K. Genetic affinities of north and northeastern populations of India: inference from HLA-based study. *Tissue Antigens* **72**, 120-130, doi:10.1111/j.1399-0039.2008.01083.x (2008).
39 Moscoso, J. *et al.* HLA genes of Aleutian Islanders living between Alaska (USA) and Kamchatka (Russia) suggest a possible southern Siberia origin. *Molecular Immunology* **45**, 1018-1026, doi:10.1016/j.molimm.2007.07.024 (2008).
40 Biro, A. Z., Zalan, A., Volgyi, A. & Pamjav, H. A Y-Chromosomal Comparison of the Madjars (Kazakhstan) and the Magyars (Hungary). *American Journal of Physical Anthropology* **139**, 305-310, doi:10.1002/ajpa.20984 (2009).
41 Arnaiz-Villena, A. *et al.* The Origin of Amerindians and the Peopling of the Americas According to HLA Genes: Admixture with Asian and Pacific People. *Current Genomics* **11**, 103-114, doi:10.2174/138920210790886862 (2010).
42 Krause, J. *et al.* The complete mitochondrial DNA genome of an unknown hominin from southern Siberia. *Nature* **464**, 894-897, doi:10.1038/nature08976 (2010).
43 Huyghe, J. R. *et al.* A genome-wide analysis of population structure in the Finnish Saami with implications for genetic association studies. *European Journal of Human Genetics* **19**, 347-352, doi:10.1038/ejhg.2010.179 (2011).
44 Suslova, T. A. *et al.* HLA gene and haplotype frequencies in Russians, Bashkirs and Tatars, living in the Chelyabinsk Region (Russian South Urals). *International Journal of Immunogenetics* **39**, 394-408, doi:10.1111/j.1744-313X.2012.01117.x (2012).
45 Honkola, T. *et al.* Cultural and climatic changes shape the evolutionary history of the Uralic languages. *Journal of Evolutionary Biology* **26**, 1244-1253, doi:10.1111/jeb.12107 (2013).
46 Regueiro, M. *et al.* Austronesian genetic signature in East African Madagascar and Polynesia. *Journal of Human Genetics* **53**, 106-120, doi:10.1007/s10038-007-0224-4 (2008).
47 Mirabal, S., Cadenas, A. M., Garcia-Bertrand, R. & Herrera, R. J. Ascertaining the Role of Taiwan as a Source for the Austronesian Expansion. *American Journal of Physical Anthropology* **150**, 551-564, doi:10.1002/ajpa.22226 (2013).
48 Omoto, K. & Saitou, N. Genetic origins of the Japanese: A partial support for the dual structure hypothesis. *American Journal of Physical Anthropology* **102**, 437-446 (1997).
49 Edinur, H. A. *et al.* HLA polymorphism in six Malay subethnic groups in Malaysia. *Human Immunology* **70**, 518-526, doi:10.1016/j.humimm.2009.04.003 (2009).

50 Mesa, N. R. *et al.* Autosomal, mtDNA, and Y-chromosome diversity in Amerinds: Pre- and post-Columbian patterns of gene flow in South America. *American Journal of Human Genetics* **67**, 1277-1286 (2000).
51 Ayub, Q. *et al.* Reconstruction of human evolutionary tree using polymorphic autosomal microsatellites. *American Journal of Physical Anthropology* **122**, 259-268, doi:10.1002/ajpa.10234 (2003).
52 Reesink, G., Singer, R. & Dunn, M. Explaining the Linguistic Diversity of Sahul Using Population Models. *Plos Biology* **7**, doi:10.1371/journal.pbio.1000241 (2009).
53 Chaubey, G. & Endicott, P. The Andaman Islanders in a Regional Genetic Context: Reexamining the Evidence for an Early Peopling of the Archipelago from South Asia. *Human Biology* **85**, 153-171 (2013).
54 Munkhbat, B. *et al.* Molecular analysis of HLA polymorphism in Khoton-Mongolians. *Tissue Antigens* **50**, 124-134, doi:10.1111/j.1399-0039.1997.tb02851.x (1997).
55 Sanchez-Velasco, P., Karadsheh, N. S., Garcia-Martin, A., de Alegria, C. R. & Leyva-Cobian, F. Molecular analysis of HLA allelic frequencies and haplotypes in Jordanians and comparison with other related populations. *Human Immunology* **62**, 901-909, doi:10.1016/s0198-8859(01)00289-0 (2001).
56 Sanchez-Velasco, P. & Leyva-Cobian, F. The HLA class I and class II allele frequencies studied at the DNA level in the Svanetian population (Upper Caucasus) and their relationships to Western European populations. *Tissue Antigens* **58**, 223-233, doi:10.1034/j.1399-0039.2001.580402.x (2001).
57 Ivanova, M. *et al.* HLA polymorphism in Bulgarians defined by high-resolution typing methods in comparison with other populations. *Tissue Antigens* **60**, 496-504, doi:10.1034/j.1399-0039.2002.600605.x (2002).
58 Arnaiz-Villena, A. *et al.* HLA genes in the chuvashian population from European Russia: Admixture of central European and Mediterranean populations. *Human Biology* **75**, 375-392, doi:10.1353/hub.2003.0040 (2003).
59 Gray, R. D. & Atkinson, Q. D. Language-tree divergence times support the Anatolian theory of Indo-European origin. *Nature* **426**, 435-439, doi:10.1038/nature02029 (2003).
60 Pagel, M. & Meade, A. in *The evolution of cultural diversity: a phylogenetic approach* (eds Ruth Mace, Clare J Holden, & Stephen Shennan) Ch. 13, 235-256 (UCL Press, 2005).
61 Fortunato, L., Holden, C. & Mace, R. From bridewealth to dowry? *Human Nature* **17**, 355-376, doi:10.1007/s12110-006-1000-4 (2006).
62 Pagel, M., Atkinson, Q. D. & Meade, A. Frequency of word-use predicts rates of lexical evolution throughout Indo-European history. *Nature* **449**, 717-U717, doi:10.1038/nature06176 (2007).
63 Serva, M. & Petroni, F. Indo-European languages tree by Levenshtein distance. *EPL (Europhysics Letters)* **81**, doi:10.1209/0295-5075/81/68005 (2008).
64 Geisler, H. & List, J.-M. in *Arbeitstagung der Indogermanischen Gesellschaft 2009: Die Ausbreitung des Indogermanischen Thesen aus Sprachwissenschaft, Archäologie und Genetik* (Würzburg, Germany, 2009).

65 Sulcebe, G. *et al.* HLA allele and haplotype frequencies in the Albanian population and their relationship with the other European populations. *International Journal of Immunogenetics* **36**, 337-343, doi:10.1111/j.1744-313X.2009.00868.x (2009).
66 Greenhill, S. J., Atkinson, Q. D., Meade, A. & Gray, R. D. The shape and tempo of language evolution. *Proceedings of the Royal Society B-Biological Sciences* **277**, 2443-2450, doi:10.1098/rspb.2010.0051 (2010).
67 Rasmussen, M. *et al.* An Aboriginal Australian Genome Reveals Separate Human Dispersals into Asia. *Science* **334**, 94-98, doi:10.1126/science.1211177 (2011).
68 Bouckaert, R. *et al.* Mapping the Origins and Expansion of the Indo-European Language Family. *Science* **337**, 957-960, doi:10.1126/science.1219669 (2012).
69 Delmestri, A. & Cristianini, N. Linguistic Phylogenetic Inference by PAM-like Matrices. *Journal of Quantitative Linguistics* **19**, 95-120, doi:10.1080/09296174.2012.659001 (2012).
70 Mendizabal, I. *et al.* Reconstructing the Population History of European Romani from Genome-wide Data. *Current Biology* **22**, 2342-2349, doi:10.1016/j.cub.2012.10.039 (2012).
71 Melton, T. *et al.* Polynesian genetic affinities with Southeast Asian populations as identified by mtDNA analysis. *American Journal of Human Genetics* **57**, 403-414 (1995).
72 Chhaya, S., Desai, S. & Saranath, D. HLA polymorphisms in Sindhi community in Mumbai, India. *International Journal of Immunogenetics* **37**, 373-377, doi:10.1111/j.1744-313X.2010.00936.x (2010).
73 Redd, A. J. & Stoneking, M. Peopling of Sahul: mtDNA variation in Aboriginal Australian and Papua New Guinean populations. *American Journal of Human Genetics* **65**, 808-828, doi:10.1086/302533 (1999).
74 Shaw, C. K., Chen, L. L., Lee, A. & Lee, T. D. Distribution of HLA gene and haplotype frequencies in Taiwan: a comparative study among Min-nan, Hakka, Aborigines and Mainland Chinese. *Tissue Antigens* **53**, 51-64, doi:10.1034/j.1399-0039.1999.530106.x (1999).
75 Nasidze, I. & Stoneking, M. Mitochondrial DNA variation and language replacements in the Caucasus. *Proceedings of the Royal Society B-Biological Sciences* **268**, 1197-1206 (2001).
76 Fu, Q. M. *et al.* DNA analysis of an early modern human from Tianyuan Cave, China. *Proceedings of the National Academy of Sciences of the United States of America* **110**, 2223-2227, doi:10.1073/pnas.1221359110 (2013).

**Supplementary Table S2.** The representative dataset. 186 populations included in the study, ISO 639-3 code, principal source study, country of origin and geographic coordinates (based on principal source study), presence of the population in the genomic source trees, and linguistic affiliation accepted from *Ethnologue*, Ruhlen, and Greenberg & Ruhlen.

| Taxon name (supertree) | ISO 639-3 | Principal source(s) | Location | Latitude (Degrees North) | Longitude (Degrees East) | Genomic source trees | Linguistic classification[1] | Linguistic classification[2,3] |
|---|---|---|---|---|---|---|---|---|
| Khwe | xuu | Schlebusch et al. (2012) | Angola | -17.363921 | 22.950439 | Y | Khoisan: Southern Africa | Khoisan |
| Ju'\|hoan | ktz | Rosenberg et al. (2002, 2005); Ramachandran et al. (2005); Schlebusch et al. (2012) | Namibia | -19.597399 | 20.494995 | Y | Khoisan: Southern Africa | Khoisan |
| !Kung | vaj, knw | Schlebusch et al. (2012) | Angola | -14.628943 | 17.666016 | Y | Khoisan: Southern Africa | Khoisan |
| ‡Khomani | ngh | Schlebusch et al. (2012) | South Africa | -26.974138 | 20.794373 | Y | Khoisan: Southern Africa | Khoisan |
| Nama | naq | Schlebusch et al. (2012) | Namibia | -22.558559 | 17.072754 | Y | Khoisan: Southern Africa | Khoisan |
| Mbuti Pygmy | efe | Rosenberg et al. (2002, 2005); Ramachandran et al. (2005) | Congo | 1 | 29 | Y | Nilo-Saharan | Nilo-Saharan |
| Aka Pygmy | axk | Rosenberg et al. (2002, 2005); Ramachandran et al. (2005) | Central African Republic | 4 | 17 | Y | Niger-Congo: Bantoid | Niger-Kordofanian |
| Fulani | fuv | Tishkoff et al. (2009) | Nigeria | 11 | 11 | Y | Niger-Congo: Atlantic | Niger-Kordofanian |
| Masana | mcn | Tishkoff et al. (2009) | Cameroon | 10.3 | 15.3 | Y | Afro-Asiatic: Chadic | Afro-Asiatic |
| Kotoko | kot | Tishkoff et al. (2009) | Cameroon | 11.8 | 14.8 | Y | Afro-Asiatic: Chadic | Afro-Asiatic |
| Podokwo | pbi | Tishkoff et al. (2009) | Cameroon | 11 | 12.1 | Y | Afro-Asiatic: Chadic | Afro-Asiatic |

| | | | | | | | | |
|---|---|---|---|---|---|---|---|---|
| Ndebele | nde | Rosenberg et al. (2002, 2005); Ramachandran et al. (2005) | South Africa | -25.5692643 | 24.25 | Y | Niger-Congo: Bantoid | Niger-Kordofanian |
| Swati | ssw | Rosenberg et al. (2002, 2005); Ramachandran et al. (2005) | South Africa | -25.5692643 | 24.25 | Y | Niger-Congo: Bantoid | Niger-Kordofanian |
| Xhosa | xho | Tishkoff et al. (2009) | South Africa | -32 | 28 | Y | Niger-Congo: Bantoid | Niger-Kordofanian |
| Zulu | zul | Rosenberg et al. (2002, 2005); Ramachandran et al. (2005) | South Africa | -25.5692643 | 24.25 | Y | Niger-Congo: Bantoid | Niger-Kordofanian |
| Mandinka | mnk | Rosenberg et al. (2002, 2005); Ramachandran et al. (2005) | Senegal | 12 | -12 | Y | Niger-Congo: Mande | Niger-Kordofanian |
| Yoruba | yor | HapMap | Nigeria | 7.4 | 3.9 | Y | Niger-Congo: Defoid | Niger-Kordofanian |
| Hausa | hau | Tishkoff et al. (2009) | Nigeria | 10 | 7 | Y | Afro-Asiatic: Chadic | Afro-Asiatic |
| Kikuyu | kik | Tishkoff et al. (2009) | Kenya | -1 | 37 | Y | Niger-Congo: Bantoid | Niger-Kordofanian |
| Dinka | dip | Tishkoff et al. (2009) | Sudan | 8 | 30 | Y | Nilo-Saharan | Nilo-Saharan |
| Shilluk | shk | Tishkoff et al. (2009) | Sudan | 10 | 32 | Y | Nilo-Saharan | Nilo-Saharan |
| Anuak | anu | Pagani et al. (2012) | Ethiopia | 8 | 34 | Y | Nilo-Saharan | Nilo-Saharan |
| Hadza | hts | Tishkoff et al. (2009) | Tanzania | -3.8 | 35.3 | Y | Khoisan: Hatsa | Khoisan |
| Sandawe | sad | Tishkoff et al. (2009) | Tanzania | -5.5 | 35.5 | Y | Khoisan: Sandawe | Khoisan |
| Maasai | mas | Tishkoff et al. (2009) | Tanzania | -4 | 37 | Y | Nilo-Saharan | Nilo-Saharan |
| Iraqw | irk | Tishkoff et al. (2009) | Tanzania | -4 | 35.5 | Y | Afro-Asiatic: Cushtic | Afro-Asiatic |
| Aari | aiw | Pagani et al. (2012) | Ethiopia | 6 | 37 | Y | Afro-Asiatic: Omotic | Afro-Asiatic |
| Dogon | dds | Tishkoff et al. (2009) | Mali | 14 | -3 | Y | Niger-Congo: | Niger-Kordofani |

| | | | | | | | | |
|---|---|---|---|---|---|---|---|---|
| | | | | | | | Dogon | an |
| Somali | som | Pagani et al. (2012) | Ethiopia | 9 | 42 | Y | Afro-Asiatic: Cushtic | Afro-Asiatic |
| Beta Israel | amh | Tishkoff et al. (2009) | Ethiopia | 12 | 38 | Y | Afro-Asiatic: Cushtic | Afro-Asiatic |
| Aweer | bob | Tishkoff et al. (2009) | Kenya | 3.5 | 37 | Y | Afro-Asiatic: Cushtic | Afro-Asiatic |
| Amhara | amh | Behar et al. (2010) | Ethiopia | 10 | 39 | Y | Afro-Asiatic: Semitic | Afro-Asiatic |
| Oromo | gax | Tishkoff et al. (2009) | Kenya | 3 | 37.5 | Y | Afro-Asiatic: Cushtic | Afro-Asiatic |
| Rendille | rel | Tishkoff et al. (2009) | Kenya | 2.3 | 37.5 | Y | Afro-Asiatic: Cushtic | Afro-Asiatic |
| Beja Hadandawa | bej | Tishkoff et al. (2009) | Sudan | 21 | 36 | Y | Afro-Asiatic: Cushtic | Afro-Asiatic |
| Berber | shi | Izaabel et al. (1998) | Morocco | 30.93 | -7.22 | Y | Afro-Asiatic: Berber | Afro-Asiatic |
| Mozabite | mzb | Rosenberg et al. (2002, 2005); Ramachandran et al. (2005) | Algeria (Mzab) | 32 | 3 | Y | Afro-Asiatic: Berber | Afro-Asiatic |
| Algerian | ara (arq) | Arnaiz-Villena et al. (1995) | Algeria | 36.75 | 3.2 | Y | Afro-Asiatic: Semitic | Afro-Asiatic |
| Moroccan | ara (ary) | Gomez-Casado et al. (2000) | Morocco | 33.2 | -8.5 | Y | Afro-Asiatic: Semitic | Afro-Asiatic |
| Egyptian | ara (arz) | Behar et al. (2010) | Egypt | 29.31 | 30.84 | Y | Afro-Asiatic: Semitic | Afro-Asiatic |
| Qatari | ara (arb) | Hunter-Zinck et al. (2010) | Qatar | 25.4 | 51.2 | Y | Afro-Asiatic: Semitic | Afro-Asiatic |
| Yemeni | ara (arb) | Behar et al. (2010) | Yemen | 15.37 | 44.19 | Y | Afro-Asiatic: Semitic | Afro-Asiatic |
| Rwala Bedouin | ayl | Rosenberg et al. (2002, 2005); Ramachandran et al. (2005) | Israel (Negev) | 31 | 35 | Y | Afro-Asiatic: Semitic | Afro-Asiatic |
| Saudi Arabian | ara (arb) | Behar et al. (2010) | Saudi Arabia | 24.72 | 46.70 | Y | Afro-Asiatic: Semitic | Afro-Asiatic |

| | | | | | | | | |
|---|---|---|---|---|---|---|---|---|
| Palestinian | ara (ajp) | Rosenberg et al. (2002, 2005); Ramachandran et al. (2005) | Israel (Central) | 32 | 35 | Y | Afro-Asiatic: Semitic | Afro-Asiatic |
| Druze | apc | Rosenberg et al. (2002, 2005); Ramachandran et al. (2005) | Israel (Carmel) | 32 | 35 | Y | Afro-Asiatic: Semitic | Afro-Asiatic |
| Armenian | hye | Behar et al. (2010) | Armenia | 40.18 | 44.51 | Y | Indo-European | Indo-Hittite |
| Ashkenazi Jew | ydd, yih | Kopelman et al. (2009) | Israel (Ashkelon) | 31.666667 | 34.566667 | Y | Indo-European | Indo-Hittite |
| Mizrahi Jew | heb | Kopelman et al. (2009) | Israel (Ashkelon) | 31.666667 | 34.566667 | Y | Afro-Asiatic: Semitic | Afro-Asiatic |
| Moroccan Jew | heb, aju | Kopelman et al. (2009) | Israel (Ashkelon) | 31.666667 | 34.566667 | Y | Afro-Asiatic: Semitic | Afro-Asiatic |
| Syrian | ara (apc) | Behar et al. (2010) | Syria | 33.51 | 36.28 | Y | Afro-Asiatic: Semitic | Afro-Asiatic |
| Lebanese | ara (apc) | Clayton et al. (1997) | Lebanon | 33.89 | 35.50 | Y | Afro-Asiatic: Semitic | Afro-Asiatic |
| Kurd | ckb | Xing et al. (2010) | Iraq | 36.74 | 43.89 | Y | Indo-European | Indo-Hittite |
| Turk | tur | Behar et al. (2010) | Turkey | 36.95 | 32.84 | Y | Altaic: Turkic | Macro-Altaic |
| Greek | ell | Mendizabal et al. (2012) | Greece | 40.6422 | 22.9456 | Y | Indo-European | Indo-Hittite |
| Romanian | ron, rup | Behar et al. (2010) | Romania | 44.43 | 26.10 | Y | Indo-European | Indo-Hittite |
| Tuscan | ita | Rosenberg et al. (2002, 2005); Ramachandran et al. (2005) | Italy | 43 | 11 | Y | Indo-European | Indo-Hittite |
| Italian | ita | Rosenberg et al. (2002, 2005); Ramachandran et al. (2005) | Italy (Bergamo) | 46 | 10 | Y | Indo-European | Indo-Hittite |
| Sardinian | src | Rosenberg et al. (2002, 2005); Ramachandran et al. (2005) | Italy | 40 | 9 | Y | Indo-European | Indo-Hittite |

| | | | | | | | | |
|---|---|---|---|---|---|---|---|---|
| French | fra | Rosenberg et al. (2002, 2005); Ramachandran et al. (2005) | France | 46 | 2 | Y | Indo-European | Indo-Hittite |
| Basque | eus | Rosenberg et al. (2002, 2005); Ramachandran et al. (2005) | France | 43 | 0 | Y | Language isolate | Dene-Caucasian |
| Spanish | spa | Martinez-Laso et al. 1995 | Spain | 40.42 | -3.70 | Y | Indo-European | Indo-Hittite |
| German | deu | Imanishi et al. (1992) | Germany | 51.34 | 12.37 | N | Indo-European | Indo-Hittite |
| English | eng | ??? | UK | 51.5 | -0.13 | Y | Indo-European | Indo-Hittite |
| Polish | pol | Nowak et al. (2008) | Poland | 51.11 | 17.03 | Y | Indo-European | Indo-Hittite |
| Hungarian | hun | Mendizabal et al., 2012 | Hungary | 47.45 | 19.04 | Y | Uralic | Uralic |
| Orcadian | sco | Rosenberg et al. (2002, 2005); Ramachandran et al. (2005) | Orkney Islands | 59 | -3 | Y | Indo-European | Indo-Hittite |
| Russian | rus | Rosenberg et al. (2002, 2005); Ramachandran et al. (2005) | Russia | 61 | 40 | Y | Indo-European | Indo-Hittite |
| Finnish | fin | Clayton et al. (1997) | Finland | 60.22 | 24.91 | N | Uralic | Uralic |
| Saami | sia, smn, sjd, sms, sjt | Evseeva et al. (2002) | Russia (Kola peninsula) | 68.00 | 35.02 | Y | Uralic | Uralic |
| Georgian | kat | Behar et al. (2010) | Georgia | 42.49 | 41.83 | Y | Kartvelian | Kartvelian |
| Adygei | ady | Rosenberg et al. (2002, 2005); Ramachandran et al. (2005) | Russia (Caucasus) | 44 | 39 | Y | North Caucasian | Dene-Caucasian: Caucasian |
| Iranian | pes | Behar et al. (2010) | Iran | 32.65 | 51.66 | Y | Indo-European | Indo-Hittite |
| Makrani | bcc | Rosenberg et al. (2002, 2005); Ramachand | Pakistan | 26 | 64 | Y | Indo-European | Indo-Hittite |

| | | | | | | | | |
|---|---|---|---|---|---|---|---|---|
| | | ran et al. (2005) | | | | | | |
| Brahui | brh | Rosenberg et al. (2002, 2005); Ramachandran et al. (2005) | Pakistan | 30.49871492 | 66.5 | Y | Dravidian | Dravidian |
| Balochi | bgp | Rosenberg et al. (2002, 2005); Ramachandran et al. (2005) | Pakistan | 30.49871492 | 66.5 | Y | Indo-European | Indo-Hittite |
| Sindhi | snd | Rosenberg et al. (2002, 2005); Ramachandran et al. (2005) | Pakistan | 25.49063551 | 69 | Y | Indo-European | Indo-Hittite |
| Pashtun | pst | Rosenberg et al. (2002, 2005); Ramachandran et al. (2005) | Pakistan | 33.48700562 | 70.5 | Y | Indo-European | Indo-Hittite |
| Kalash | kls | Rosenberg et al. (2002, 2005); Ramachandran et al. (2005) | Pakistan | 35.99366014 | 71.5 | Y | Indo-European | Indo-Hittite |
| Madiga | tel | Reich et al. (2009) | India (Andhra Pradesh) | 17.58 | 79.35 | Y | Dravidian | Dravidian |
| Tamil | tam | Rosenberg et al. (2006) | India (Tamil Nadu) | 11.10167224 | 77.94827586 | Y | Dravidian | Dravidian |
| Malayalam | mal | Rosenberg et al. (2006) | India (Kerala) | 10 | 76.25 | Y | Dravidian | Dravidian |
| Burusho | bsk | Rosenberg et al. (2002, 2005); Ramachandran et al. (2005) | Pakistan | 36.49838568 | 74 | Y | Language isolate | Dene-Caucasian |
| Hazara | haz | Rosenberg et al. (2002, 2005); Ramachandran et al. (2005) | Pakistan | 33.49855601 | 70 | Y | Indo-European | Indo-Hittite |
| Uyghur | uig | Rosenberg et al. (2002, 2005); | China | 44 | 81 | Y | Altaic: Turkic | Macro-Altaic |

| | | | | | | | | |
|---|---|---|---|---|---|---|---|---|
| | | Ramachandran et al. (2005) | | | | | | |
| Kensiu | kns | The HUGO Pan-Asian SNP Consortium | Malaysia | 5.7 | 100.9 | Y | Austro-Asiatic: Mon-Khmer | Austric: Austroasiatic |
| Jehai | jhi | The HUGO Pan-Asian SNP Consortium | Malaysia | 5.4 | 101.1 | Y | Austro-Asiatic: Mon-Khmer | Austric: Austroasiatic |
| Amis | ami | Friedlaender et al. (2008) | Taiwan | 23.3 | 121 | Y | Austronesian | Austric: Austronesian |
| Paiwan | pwn | Gray et al. (2009) | Taiwan | 22.6 | 120.8 | N | Austronesian | Austric: Austronesian |
| Taroko | trv | Friedlaender et al. (2008) | Taiwan | 23.3 | 121 | Y | Austronesian | Austric: Austronesian |
| Atayal | tay | The HUGO Pan-Asian SNP Consortium | Taiwan | 24.6 | 121.4 | N | Austronesian | Austric: Austronesian |
| Tagalog | tgl | The HUGO Pan-Asian SNP Consortium | Philiippines | 14.6 | 121.0 | Y | Austronesian: Malayo-Polynesian | Austric: Austronesian |
| Cebuano | vis | The HUGO Pan-Asian SNP Consortium | Philippines | 6.9 | 122.1 | Y | Austronesian: Malayo-Polynesian | Austric: Austronesian |
| Toraja | sda, rob | The HUGO Pan-Asian SNP Consortium | Indonesia | -4.7 | 119.7 | Y | Austronesian: Malayo-Polynesian | Austric: Austronesian |
| Bugis | bug | Hatin et al. (2011) | Malaysia | 2.04 | 103.31 | N | Austronesian: Malayo-Polynesian | Austric: Austronesian |
| Dayak Ngaju | nij | Gray et al. (2009) | Indonesia (Northwest Kalimantan) | -1.9 | 114.5 | N | Austronesian: Malayo-Polynesian | Austric: Austronesian |
| Malagasy | plt | Gray et al. (2009) | Madagascar | -18.9 | 47.5 | N | Austronesian: Malayo-Polynesian | Austric: Austronesian |
| Melayu | zsm | The HUGO Pan-Asian SNP Consortium | Indonesia | -3.0 | 104.7 | Y | Austronesian: Malayo-Polynesian | Austric: Austronesian |
| Iban | | Xing et al. (2009) | Malaysia (Sarawak) | 3 | 113 | N | Austronesian: Malayo- | Austric: Austronesian |

| | | | | | | | | |
|---|---|---|---|---|---|---|---|---|
| | | | | | | | Polynesian | |
| Singapore Malay | zlm | The HUGO Pan-Asian SNP Consortium | Singapore | 1.4 | 103.8 | Y | Austronesian: Malayo-Polynesian | Austric: Austronesian |
| Malay | zlm | The HUGO Pan-Asian SNP Consortium | Malaysia | 5.3 | 102.0 | Y | Austronesian: Malayo-Polynesian | Austric: Austronesian |
| Javanese | jav | The HUGO Pan-Asian SNP Consortium | Indonesia | -6.2 | 106.7 | Y | Austronesian: Malayo-Polynesian | Austric: Austronesian |
| Bidayuh | sne | The HUGO Pan-Asian SNP Consortium | Malaysia | 1.4 | 110.2 | Y | Austronesian: Malayo-Polynesian | Austric: Austronesian |
| Mamanwa | mmn | The HUGO Pan-Asian SNP Consortium | Philiippines | 9.7 | 125.6 | Y | Austronesian: Malayo-Polynesian | Austric: Austronesian |
| Agta | agt | The HUGO Pan-Asian SNP Consortium | Philiippines | 13.7 | 123.3 | Y | Austronesian: Malayo-Polynesian | Austric: Austronesian |
| Aeta | sbl | The HUGO Pan-Asian SNP Consortium | Philiippines | 14.9 | 120.2 | Y | Austronesian: Malayo-Polynesian | Austric: Austronesian |
| Andamanese | oon, anq, gac | Rosenberg et al. (2006) | India (Andaman Islands) | 10.30 | 92.30 | Y | Andamanese | Indo-Pacific |
| Naasioi | nas | Rosenberg et al. (2002, 2005); Friedlaender et al. (2008) | Bougainville | -6.483 | 155.833 | Y | South Bougainville | Indo-Pacific: East Papuan |
| Goroka | for, gim | Rosenberg et al. (2002, 2005); Friedlaender et al. (2008) | New Guinea | -6.083 | 145.4 | Y | Trans-New Guinea | Indo-Pacific |
| Ngarinyin | ung | Reesink et al. (2009) | Australia | -16.4 | 126.4 | N | Australian | Australian |
| Warlpiri | wbp | Reesink et al. (2009) | Australia | -23.7 | 133.9 | N | Australian: Pama-Nyungan | Australian |
| AUSTRALIAN | | Rasmussen et al. (2011) | Australia | ? | ? | Y | Australian | Australian |
| Manggarai | mqy | The HUGO Pan-Asian SNP Consortium | Indonesia | -8.6 | 120.1 | Y | Austronesian: Malayo-Polynesian | Austric: Austronesian |

| | | | | | | | | |
|---|---|---|---|---|---|---|---|---|
| Kambera | xbr | The HUGO Pan-Asian SNP Consortium | Indonesia | -9.8 | 120.0 | Y | Austronesian: Malayo-Polynesian | Austric: Austronesian |
| Alorese | aol | The HUGO Pan-Asian SNP Consortium | Indonesia | -8.3 | 124.7 | Y | Austronesian: Malayo-Polynesian | Austric: Austronesian |
| Lembata | lmf, lmj | The HUGO Pan-Asian SNP Consortium | Indonesia | -8.3 | 124.7 | Y | Austronesian: Malayo-Polynesian | Austric: Austronesian |
| Kosraean | kos | Friedlaender et al. (2008) | Micronesia | 5.31 | 163 | Y | Austronesian: Malayo-Polynesian | Austric: Austronesian |
| Samoan | smo | Friedlaender et al. (2008) | Polynesia | -13.35 | -172.2 | Y | Austronesian: Malayo-Polynesian | Austric: Austronesian |
| Tongan | ton | Gray et al. (2009) | Polynesia | -21.18 | -175.20 | Y | Austronesian: Malayo-Polynesian | Austric: Austronesian |
| Hawaiian | haw | Gray et al. (2009) | Polynesia | 21.30 | -157.85 | N | Austronesian: Malayo-Polynesian | Austric: Austronesian |
| Maori | mri | Friedlaender et al. (2008) | Polynesia | -41 | 174 | Y | Austronesian: Malayo-Polynesian | Austric: Austronesian |
| Rumai | rbb | The HUGO Pan-Asian SNP Consortium | Thailand | 19.9 | 99.2 | Y | Austro-Asiatic: Mon-Khmer | Austric: Austroasiatic |
| Lawa | lwl, lcp | The HUGO Pan-Asian SNP Consortium | Thailand | 18.4 | 98.1 | Y | Austro-Asiatic: Mon-Khmer | Austric: Austroasiatic |
| Yakut | sah | Rosenberg et al. (2002, 2005); Ramachandran et al. (2005) | Siberia | 62.98287845 | 129.5 | Y | Altaic: Turkic | Macro-Altaic |
| Tuvan | tyv | Martinez-Laso et al. (2001) | Russia (Republic of Tuva) | 51.71 | 94.45 | Y | Altaic: Turkic | Macro-Altaic |
| Nenets | yrk | Wang et al. (2008) | Siberia | 66.08 | 76.5 | Y | Uralic | Uralic |
| Khalkha Mongol | khk, mvn | Rosenberg et al. (2002, 2005); Ramachandran et al. | Mongolia | 48 | 107 | Y | Altaic: Mongolic | Macro-Altaic |

| | | | | | | | | |
|---|---|---|---|---|---|---|---|---|
| | | (2005); Reich et al. (2012) | | | | | | |
| Daur | tdd | Rosenberg et al. (2002, 2005); Ramachandran et al. (2005) | China | 48.49753416 | 124 | Y | Altaic: Mongolic | Macro-Altaic |
| Hezhen | gld | Rosenberg et al. (2002, 2005); Ramachandran et al. (2005) | China | 47.4976192 | 133.5 | Y | Altaic: Tungusic | Macro-Altaic |
| Oroqen | orh | Rosenberg et al. (2002, 2005); Ramachandran et al. (2005) | China | 50.43389257 | 126.5 | Y | Altaic: Tungusic | Macro-Altaic |
| Xibo | sjo | Rosenberg et al. (2002, 2005); Ramachandran et al. (2005) | China | 43.49792973 | 81.5 | Y | Altaic: Tungusic | Macro-Altaic |
| Tu | mjg | Rosenberg et al. (2002, 2005); Ramachandran et al. (2005) | China | 36 | 101 | Y | Altaic: Mongolic | Macro-Altaic |
| Yi | yif | Rosenberg et al. (2002, 2005); Ramachandran et al. (2005) | China | 28 | 103 | Y | Sino-Tibetan: Tibeto-Burman | Sino-Tibetan |
| Naxi | nbf | Rosenberg et al. (2002, 2005); Ramachandran et al. (2005) | China | 26 | 100 | Y | Sino-Tibetan: Tibeto-Burman | Sino-Tibetan |
| Tibetan | bod | Kang et al. (2010) | China | 29.65 | 91.17 | Y | Sino-Tibetan: Tibeto-Burman | Sino-Tibetan |
| Korean | kor | The HUGO Pan-Asian SNP Consortium | Korea | 36.9 | 127.5 | Y | Koreanic | Macro-Altaic |
| Japanese | jpn | HapMap | Japan (Honshu) | 35.7 | 139.8 | Y | Japonic | Macro-Altaic |

| | | | | | | | | |
|---|---|---|---|---|---|---|---|---|
| Ainu | ain | Bannai et al. (200) | Japan (Hokkaido) | 42.35 | 142.39 | Y | Language isolate | Eurasiatic |
| Northern Han Chinese | cmn | Rosenberg et al. (2002, 2005); Ramachandran et al. (2005) | China | 32.26566812 | 114 | Y | Sino-Tibetan: Chinese | Sino-Tibetan |
| Min nan Taiwanese' | nan | The HUGO Pan-Asian SNP Consortium | Taiwan | 25.0 | 121.5 | Y | Sino-Tibetan: Chinese | Sino-Tibetan |
| Southern Han Chinese | cmn, yue | The HUGO Pan-Asian SNP Consortium | China | 23.3 | 113.5 | Y | Sino-Tibetan: Chinese | Sino-Tibetan |
| Singapore Chinese | cmn | The HUGO Pan-Asian SNP Consortium | Singapore | 1.4 | 103.8 | Y | Sino-Tibetan: Chinese | Sino-Tibetan |
| Tujia | tji | Rosenberg et al. (2002, 2005); Ramachandran et al. (2005) | China | 29 | 109 | Y | Sino-Tibetan: Tibeto-Burman | Sino-Tibetan |
| She | shx | Rosenberg et al. (2002, 2005); Ramachandran et al. (2005) | China | 27 | 119 | Y | Hmong-Mien | Austric: Miao-Yao |
| Miao | hmy | Rosenberg et al. (2002, 2005); Ramachandran et al. (2005) | China | 28 | 109 | Y | Hmong-Mien | Austric: Miao-Yao |
| Dai | tdd | Rosenberg et al. (2002, 2005); Ramachandran et al. (2005) | China | 21 | 100 | Y | Tai-Kadai | Austric: Daic |
| Lahu | lhu | Rosenberg et al. (2002, 2005); Ramachandran et al. (2005) | China | 22 | 100 | Y | Sino-Tibetan: Tibeto-Burman | Sino-Tibetan |
| Cambodian | khm | Rosenberg et al. (2002, 2005); Ramachandran et al. | Cambodia | 12 | 105 | Y | Austro-Asiatic: Mon-Khmer | Austric |

| | | | | | | | | |
|---|---|---|---|---|---|---|---|---|
| | | (2005) | | | | | | |
| Evenki | evn | Grahovac et al. (1998); Reich et al. (2012) | Siberia (Evenkiysky District) | 64.1 | 95.4 | Y | Altaic: Tungusic | Macro-Altaic |
| Yukaghir | ykg | Reich et al. (2012) | Siberia (Srednekolymsky District) | 68 | 150 | Y | Yukaghir | Uralic |
| Ket | ket | Grahovac et al. (1998); Reich et al. (2012) | Siberia (Evenkiysky District) | 63.8 | 87.4 | Y | Yeniseian | Dene-Caucasian: Caucasian |
| Tlingit | tli | Imanishi et al. (1992) | Canada (British Colombia) | 51 | -123 | N | Eyak-Athabascan | Na-Dene |
| Nivkh | niv | Grahovac et al. (1998) | Siberia (Sakhalin Island) | 52.7 | 142.8 | N | Language isolate | Eurasiatic |
| Dakelh | crx, bcr | Monsalve et al. (1998) | Canada (British Colombia) | 53 | -123 | N | Eyak-Athabascan: Athabascan | Na-Dene |
| Koryak | kpy | Grahovac et al. (1998); Reich et al. (2012) | Siberia | 59.4 | 163 | Y | Chukotko-Kamchatkan | Chukchi-Kamchatkan |
| Chukchi | ckt | Grahovac et al. (1998); Reich et al. (2012) | Siberia (Providensky District) | 65.1 | -173.5 | Y | Chukotko-Kamchatkan | Chukchi-Kamchatkan |
| Siberian Yupik | ess, ynk, ysr | Grahovac et al. (1998) | Siberia (Chukotsky District) | 66 | -175.2 | Y | Eskimo-Aleut | Eskimo-Aleut |
| Greenland Inuit | kal | Reich et al. (2012) | Greenland | 65.3 | -52 | Y | Eskimo-Aleut | Eskimo-Aleut |
| Haida | hai (hdn, hax) | Ward et al. (1993) | Canada | 53.25 | -132 | N | Haida | Dene-Caucasian: Na-Dene |
| Dene | chp | Wang et al. (2008) | Canada | 59.55 | -107.3 | Y | Eyak-Athabascan: Athabascan | Dene-Caucasian: Na-Dene |
| Navajo | nav | Budowle et al. (2002) | USA (South Dakota) | 35.66 | -109.07 | N | Eyak-Athabascan: Athabascan | Dene-Caucasian: Na-Dene: |
| Cree | cre | Wang et al. (2008) | Canada | 50.33 | -102.5 | Y | Algic | Amerind: Almosan |

| | | | | | | | | |
|---|---|---|---|---|---|---|---|---|
| Ojibwa | ojc, ojg, ojb, ojs | Wang et al. (2008) | Canada | 46.5 | -81 | Y | Algic | Amerind: Almosan |
| Mapuche | huh, arn | Wang et al. (2008) | Chile | -41 | -73 | Y | Mapudungu | Amerind: Andean |
| Pima | pia | Rosenberg et al. (2002, 2005); Ramachandran et al. (2005) | Mexico | 29 | -108 | Y | Uto-Aztecan | Amerind: Central |
| Yucatec Maya | yua | Rosenberg et al. (2002, 2005); Ramachandran et al. (2005) | Mexico | 19 | -91 | Y | Mayan | Amerind: Penutian |
| Lakota | lkt | Leffell et al. (2004) | USA (South Dakota) | 35.66 | -109.07 | N | Siouan-Catawban | Amerind: Keresiouan |
| Aymara | aym (ayr, ayc) | Wang et al. (2008) | Chile | -22 | -70 | Y | Aymaran | Amerind: Andean |
| Quechua | que | Wang et al. (2008) | Peru | -14 | -74 | Y | Quechuan | Amerind: Andean |
| Tehuelche | teh | Reich et al. (2012) | Chile | -45 | -74 | Y | Chon | Amerind: Andean |
| Yahgan | yag | Reich et al. (2012) | Chile | -55 | -68 | Y | Language isolate | Amerind: Andean |
| Cherokee | chr | Malhi et al. (2001) | USA (Oklahoma) | 35.81 | -94.63 | N | Iroquoian | Amerind: Keresiouan |
| Mixtec | mix | Wang et al. (2008) | Mexico | 17 | -97 | Y | Otomanguean | Amerind: Central |
| Zapotec | several dialects | Wang et al. (2008) | Mexico | 16 | -97 | Y | Otomanguean | Amerind: Central |
| Kaingang | xok, zkp | Wang et al. (2008) | Brazil | -24 | -52.5 | Y | Jean | Amerind: Macro-Ge |
| Arhuaco | arc | Wang et al. (2008) | Colombia | 11 | -73.8 | Y | Chibchan | Amerind: Chibchan |
| Kogi | kog | Wang et al. (2008) | Colombia | 11 | -74 | Y | Chibchan | Amerind: Chibchan |
| Guarani | grn | Wang et al. (2008) | Brazil | -23 | -54 | Y | Tupian | Amerind: Equatorial |
| Wichi | mzh, mpt, wlv | Cerna et al. (1993) | Argentina | -31 | -59 | Y | Matacoan | Amerind: Macro-Panoan |
| Toba | tob | Cerna et al. (1993) | Argentina | -28 | -59 | Y | Guaykuruan | Amerind: Macro-Panoan |
| Piapoco | pio | Rosenberg et al. (2002, 2005); | Colombia | 3 | -68 | Y | Maipurean | Amerind: Equatorial |

| | | | | | | | | |
|---|---|---|---|---|---|---|---|---|
| | | Ramachandran et al. (2005) | | | | | | |
| Curripaco | kpc | Rosenberg et al. (2002, 2005); Ramachandran et al. (2005) | Colombia | 3 | -68 | Y | Maipurean | Amerind: Equatorial |
| Karitiana | ktn | Rosenberg et al. (2002, 2005); Ramachandran et al. (2005) | Brazil | -10 | -63 | Y | Tupian | Amerind: Equatorial |
| Surui | sru | Rosenberg et al. (2002, 2005); Ramachandran et al. (2005) | Brazil | -11 | -62 | Y | Tupian | Amerind: Equatorial |
| Ache | guq | Wang et al. (2008) | Paraguay | -24 | -56 | Y | Tupian | Amerind: Equatorial |
| Waiwai | waw | Hutz et al. (2002) | Brazil | 0.4 | -57.55 | N | Cariban | Amerind: Macro-Carib |

## References


1 Lewis, M., Simons, G. & Fennig, C. *Ethnologue: Languages of the World, Seventeenth Edition*. (SIL international Dallas, TX, 2013).

2 Ruhlen, M. *A guide to the world's languages: classification*. Vol. 1 (Stanford University Press, 1991).

3 Greenberg, J. H. & Ruhlen, M. *An Amerind Etymological Dictionary*. (Stanford University, Department of Anthropological Sciences, Stanford, 2007).

**Supplementary Table S3.** Resolution (number of nodes), tree length, consistency index (CI), and retention index (RI) for 16 semistrict consensus supertrees based on the sensitivity analysis. (**a**) Representative dataset. (**b**) Representative dataset excluding four wildcard taxa. (**c**) HGDP dataset.

**a**

| | **Number of nodes (max 185)** | **Tree length** | **CI** | **RI** |
|---|---|---|---|---|
| **1.A** | 164 | 11764 | 0.538 | 0.838 |
| **1.B** | 164 | 11820 | 0.536 | 0.837 |
| **1.C** | 120 | 15954 | .0397 | 0.714 |
| **1.D** | 111 | 18620 | 0.340 | 0.635 |
| **2.A** | 149 | 12520 | 0.506 | 0.816 |
| **2.B** | 148 | 12539 | 0.505 | 0.815 |
| **2.C** | 146 | 12978 | 0.488 | 0.802 |
| **2.D** | 146 | 12872 | 0.492 | 0.805 |
| **3.A** | 159 | 12200 | 0.519 | 0.825 |
| **3.B** | 148 | 13399 | 0.472 | 0.790 |
| **3.C** | 148 | 13399 | 0.472 | 0.790 |
| **3.D** | 162 | 12191 | 0.519 | 0.826 |
| **4.A** | 148 | 12863 | 0.492 | 0.806 |
| **4.B** | 148 | 12863 | 0.492 | 0.806 |
| **4.C** | 160 | 12863 | 0.507 | 0.817 |
| **4.D** | 155 | 12483 | 0.503 | 0.814 |

**b**

| | **Number of nodes (max 181)** | **Tree length** | **CI** | **RI** |
|---|---|---|---|---|
| **1.A** | 160 | 11716 | 0.537 | 0.838 |
| **1.B** | 160 | 11726 | 0.537 | 0.838 |
| **1.C** | 152 | 11921 | 0.528 | 0.832 |
| **1.D** | 155 | 11803 | 0.533 | 0.836 |
| **2.A** | 165 | 11730 | 0.537 | 0.838 |
| **2.B** | 164 | 11749 | 0.536 | 0.837 |
| **2.C** | 163 | 11982 | 0.525 | 0.830 |
| **2.D** | 162 | 11957 | 0.526 | 0.831 |
| **3.A** | 165 | 11940 | 0.527 | 0.832 |
| **3.B** | 163 | 11966 | 0.526 | 0.831 |
| **3.C** | 163 | 11966 | 0.526 | 0.831 |
| **3.D** | 164 | 12006 | 0.524 | 0.830 |
| **4.A** | 161 | 12165 | 0.517 | 0.825 |
| **4.B** | 161 | 12165 | 0.517 | 0.825 |
| **4.C** | 161 | 12156 | 0.517 | 0.825 |
| **4.D** | 162 | 12105 | 0.520 | 0.827 |

**c**

| | **Number of nodes (max 54)** | **Tree length** | **CI** | **RI** |
|---|---|---|---|---|
| **1.A** | 50 | 5953 | 0.605 | 0.857 |
| **1.B** | 54 | 5912 | 0.609 | 0.860 |
| **1.C** | 54 | 5912 | 0.609 | 0.860 |
| **1.D** | 54 | 5887 | 0.612 | 0.861 |
| **2.A** | 53 | 5956 | 0.605 | 0.857 |
| **2.B** | 53 | 5956 | 0.605 | 0.857 |
| **2.C** | 53 | 5956 | 0.605 | 0.857 |
| **2.D** | 52 | 5953 | 0.605 | 0.857 |
| **3.A** | 53 | 5956 | 0.605 | 0.857 |
| **3.B** | 53 | 5956 | 0.605 | 0.857 |
| **3.C** | 53 | 5956 | 0.605 | 0.857 |
| **3.D** | 52 | 5953 | 0.605 | 0.857 |
| **4.A** | 53 | 6035 | 0.597 | 0.852 |
| **4.B** | 53 | 6035 | 0.597 | 0.852 |
| **4.C** | 53 | 6035 | 0.597 | 0.852 |
| **4.D** | 53 | 6052 | 0.595 | 0.851 |

**Supplementary Table S4.** Similarity of the resulting supertrees based on sensitivity analyses measured by SPR distances. Number of SPR moves and percentage similarity between the two supertrees (in parentheses). (**a**) Representative dataset. (**b**) Representative dataset excluding four wildcard taxa. (**c**) HGDP dataset.

**a**

| | **1.A** | **1.B** | **1.C** | **1.D** | **2.A** | **2.B** | **2.C** | **2.D** | **3.A** | **3.B** | **3.C** | **3.D** | **4.A** | **4.B** | **4.C** | **4.D** |
|---|---|---|---|---|---|---|---|---|---|---|---|---|---|---|---|---|
| **1.A** | - | 9 (0.9519) | 8 (0.9572) | 10 (0.9465) | 28 (0.8503) | 28 (0.8503) | 37 (0.8021) | 37 (0.8021) | 38 (0.7968) | 38 (0.7968) | 38 (0.7968) | 49 (0.7380) | 38 (0.7968) | 40 (0.7861) | 42 (0.7754) | 40 (0.7861) |
| **1.B** | 9 (0.9519) | - | 6 (0.9679) | 8 (0.9572) | 26 (0.8610) | 26 (0.8610) | 34 (0.8182) | 32 (0.8289) | 36 (0.8075) | 33 (0.8235) | 34 (0.8182) | 45 (0.7594) | 39 (0.7914) | 39 (0.7914) | 43 (0.7701) | 41 (0.7807) |
| **1.C** | 8 (0.9572) | 6 (0.9679) | - | 3 (0.9840) | 14 (0.9251) | 15 (0.9198) | 20 (0.8930) | 24 (0.8717) | 24 (0.8717) | 24 (0.8717) | 23 (0.8770) | 29 (0.8449) | 26 (0.8610) | 27 (0.8556) | 28 (0.8503) | 28 (0.8503) |
| **1.D** | 10 (0.9465) | 8 (0.9572) | 3 (0.9840) | - | 14 (0.9251) | 15 (0.9198) | 18 (0.9037) | 17 (0.9091) | 22 (0.8824) | 20 (0.8930) | 20 (0.8930) | 23 (0.8770) | 24 (0.8717) | 24 (0.8717) | 27 (0.8556) | 23 (0.8770) |
| **2.A** | 28 (0.8503) | 26 (0.8610) | 14 (0.9251) | 14 (0.9251) | - | 1 (0.9947) | 13 (0.9305) | 16 (0.9144) | 18 (0.9037) | 18 (0.9037) | 18 (0.9037) | 25 (0.8663) | 35 (0.8128) | 36 (0.8075) | 37 (0.8021) | 40 (0.7861) |
| **2.B** | 28 (0.8503) | 26 (0.8610) | 15 (0.9198) | 15 (0.9198) | 1 (0.9947) | - | 13 (0.9305) | 16 (0.9144) | 18 (0.9037) | 17 (0.9091) | 18 (0.9037) | 25 (0.8663) | 35 (0.8128) | 35 (0.8128) | 37 (0.8021) | 40 (0.7861) |
| **2.C** | 37 (0.8021) | 34 (0.8182) | 20 (0.8930) | 18 (0.9037) | 13 (0.9305) | 13 (0.9305) | - | 9 (0.9519) | 21 (0.8877) | 20 (0.8930) | 19 (0.8984) | 34 (0.8182) | 36 (0.8075) | 37 (0.8021) | 39 (0.7914) | 43 (0.7701) |
| **2.D** | 37 (0.8021) | 32 (0.8289) | 24 (0.8717) | 17 (0.9091) | 16 (0.9144) | 16 (0.9144) | 9 (0.9519) | - | 20 (0.8930) | 20 (0.8930) | 20 (0.8930) | 30 (0.8396) | 38 (0.7968) | 38 (0.7968) | 38 (0.7968) | 39 (0.7914) |
| **3.A** | 38 (0.7968) | 36 (0.8075) | 24 (0.8717) | 22 (0.8824) | 18 (0.9037) | 18 (0.9037) | 21 (0.8877) | 20 (0.8930) | - | 2 (0.9893) | 2 (0.9893) | 13 (0.9305) | 40 (0.7861) | 41 (0.7807) | 43 (0.7701) | 41 (0.7807) |
| **3.B** | 38 (0.7968) | 33 (0.8235) | 24 (0.8717) | 20 (0.8930) | 18 (0.9037) | 17 (0.9091) | 20 (0.8930) | 20 (0.8930) | 2 (0.9893) | - | 1 (0.9947) | 12 (0.9358) | 38 (0.7968) | 38 (0.7968) | 41 (0.7807) | 39 (0.7914) |
| **3.C** | 38 (0.7968) | 34 (0.8182) | 23 (0.8770) | 20 (0.8930) | 18 (0.9037) | 18 (0.9037) | 19 (0.8984) | 20 (0.8930) | 2 (0.9893) | 1 (0.9947) | - | 12 (0.9358) | 38 (0.7968) | 39 (0.7914) | 40 (0.7861) | 39 (0.7914) |
| **3.D** | 49 (0.7380) | 45 (0.7594) | 29 (0.8449) | 23 (0.8770) | 25 (0.8663) | 25 (0.8663) | 34 (0.8182) | 30 (0.8396) | 13 (0.9305) | 12 (0.9358) | 12 (0.9358) | - | 45 (0.7594) | 46 (0.7540) | 49 (0.7380) | 42 (0.7754) |
| **4.A** | 38 (0.7968) | 39 (0.7914) | 26 (0.8610) | 24 (0.8717) | 35 (0.8128) | 35 (0.8128) | 36 (0.8075) | 38 (0.7968) | 40 (0.7861) | 38 (0.7968) | 38 (0.7968) | 45 (0.7594) | - | 1 (0.9947) | 2 (0.9893) | 6 (0.9679) |
| **4.B** | 40 (0.7861) | 39 (0.7914) | 27 (0.8556) | 24 (0.8717) | 36 (0.8075) | 35 (0.8128) | 37 (0.8021) | 38 (0.7968) | 41 (0.7807) | 38 (0.7968) | 39 (0.7914) | 46 (0.7540) | 1 (0.9947) | - | 2 (0.9893) | 6 (0.9679) |
| **4.C** | 42 (0.7754) | 43 (0.7701) | 28 (0.8503) | 27 (0.8556) | 37 (0.8021) | 37 (0.8021) | 39 (0.7914) | 38 (0.7968) | 43 (0.7701) | 41 (0.7807) | 40 (0.7861) | 49 (0.7380) | 2 (0.9893) | 2 (0.9893) | - | 7 (0.9626) |
| **4.D** | 40 (0.7861) | 41 (0.7807) | 28 (0.8503) | 23 (0.8770) | 40 (0.7861) | 40 (0.7861) | 43 (0.7701) | 39 (0.7914) | 41 (0.7807) | 39 (0.7914) | 39 (0.7914) | 42 (0.7754) | 6 (0.9679) | 6 (0.9679) | 7 (0.9626) | - |

**b**

| | 1.A | 1.B | 1.C | 1.D | 2.A | 2.B | 2.C | 2.D | 3.A | 3.B | 3.C | 3.D | 4.A | 4.B | 4.C | 4.D |
|---|---|---|---|---|---|---|---|---|---|---|---|---|---|---|---|---|
| **1.A** | - | 8 (0.9563) | 11 (0.9399) | 14 (0.9235) | 27 (0.8525) | 28 (0.8470) | 44 (0.7596) | 46 (0.7486) | 35 (0.8087) | 38 (0.7923) | 38 (0.7923) | 45 (0.7541) | 40 (0.7814) | 41 (0.7760) | 41 (0.7760) | 41 (0.7760) |
| **1.B** | 8 (0.9563) | - | 12 (0.9344) | 15 (0.9180) | 25 (0.8634) | 25 (0.8634) | 41 (0.7760) | 39 (0.7869) | 35 (0.8087) | 34 (0.8142) | 35 (0.8087) | 43 (0.7650) | 41 (0.7760) | 41 (0.7760) | 42 (0.7705) | 42 (0.7705) |
| **1.C** | 11 (0.9399) | 12 (0.9344) | - | 3 (0.9836) | 30 (0.8361) | 31 (0.8306) | 36 (0.8033) | 38 (0.7923) | 30 (0.8361) | 30 (0.8361) | 29 (0.8415) | 36 (0.8033) | 36 (0.8033) | 37 (0.7978) | 36 (0.8033) | 36 (0.8033) |
| **1.D** | 14 (0.9235) | 15 (0.9180) | 3 (0.9836) | - | 30 (0.8361) | 31 (0.8306) | 38 (0.7923) | 34 (0.8142) | 33 (0.8197) | 31 (0.8306) | 31 (0.8306) | 34 (0.8142) | 39 (0.7869) | 40 (0.7814) | 40 (0.7814) | 36 (0.8033) |
| **2.A** | 27 (0.8525) | 25 (0.8634) | 30 (0.8361) | 30 (0.8361) | - | 1 (0.9945) | 25 (0.8634) | 24 (0.8689) | 17 (0.9071) | 17 (0.9071) | 17 (0.9071) | 22 (0.8798) | 40 (0.7814) | 41 (0.7760) | 41 (0.7760) | 43 (0.7650) |
| **2.B** | 28 (0.8470) | 25 (0.8634) | 31 (0.8306) | 31 (0.8306) | 1 (0.9945) | - | 25 (0.8634) | 24 (0.8689) | 17 (0.9071) | 16 (0.9126) | 17 (0.9071) | 22 (0.8798) | 40 (0.7814) | 40 (0.7814) | 41 (0.7760) | 43 (0.7650) |
| **2.C** | 44 (0.7596) | 41 (0.7760) | 36 (0.8033) | 38 (0.7923) | 25 (0.8634) | 25 (0.8634) | - | 5 (0.9727) | 22 (0.8798) | 21 (0.8852) | 20 (0.8907) | 33 (0.8197) | 43 (0.7650) | 44 (0.7596) | 43 (0.7650) | 44 (0.7596) |
| **2.D** | 46 (0.7486) | 39 (0.7869) | 38 (0.7923) | 34 (0.8142) | 24 (0.8689) | 24 (0.8689) | 5 (0.9727) | - | 25 (0.8634) | 24 (0.8689) | 24 (0.8689) | 33 (0.8197) | 49 (0.7322) | 50 (0.7268) | 50 (0.7268) | 47 (0.7432) |
| **3.A** | 35 (0.8087) | 35 (0.8087) | 30 (0.8361) | 33 (0.8197) | 17 (0.9071) | 17 (0.9071) | 22 (0.8798) | 25 (0.8634) | - | 2 (0.9891) | 2 (0.9891) | 11 (0.9399) | 40 (0.7814) | 41 (0.7760) | 41 (0.7760) | 40 (0.7814) |
| **3.B** | 38 (0.7923) | 34 (0.8142) | 30 (0.8361) | 31 (0.8306) | 17 (0.9071) | 16 (0.9126) | 21 (0.8852) | 24 (0.8689) | 2 (0.9891) | - | 1 (0.9945) | 10 (0.9454) | 40 (0.7814) | 40 (0.7814) | 41 (0.7760) | 40 (0.7814) |
| **3.C** | 38 (0.7923) | 35 (0.8087) | 29 (0.8415) | 31 (0.8306) | 17 (0.9071) | 17 (0.9071) | 20 (0.8907) | 24 (0.8689) | 2 (0.9891) | 1 (0.9945) | - | 10 (0.9454) | 40 (0.7814) | 41 (0.7760) | 40 (0.7814) | 40 (0.7814) |
| **3.D** | 45 (0.7541) | 43 (0.7650) | 36 (0.8033) | 34 (0.8142) | 22 (0.8798) | 22 (0.8798) | 33 (0.8197) | 33 (0.8197) | 11 (0.9399) | 10 (0.9454) | 10 (0.9454) | - | 44 (0.7596) | 45 (0.7541) | 45 (0.7541) | 42 (0.7705) |
| **4.A** | 40 (0.7814) | 41 (0.7760) | 36 (0.8033) | 39 (0.7869) | 40 (0.7814) | 40 (0.7814) | 43 (0.7650) | 49 (0.7322) | 40 (0.7814) | 40 (0.7814) | 40 (0.7814) | 44 (0.7596) | - | 1 (0.9945) | 1 (0.9945) | 4 (0.9781) |
| **4.B** | 41 (0.7760) | 41 (0.7760) | 37 (0.7978) | 40 (0.7814) | 41 (0.7760) | 40 (0.7814) | 44 (0.7596) | 50 (0.7268) | 41 (0.7760) | 40 (0.7814) | 41 (0.7760) | 45 (0.7541) | 1 (0.9945) | - | 1 (0.9945) | 4 (0.9781) |
| **4.C** | 41 (0.7760) | 42 (0.7705) | 36 (0.8033) | 40 (0.7814) | 41 (0.7760) | 41 (0.7760) | 43 (0.7650) | 50 (0.7268) | 41 (0.7760) | 41 (0.7760) | 40 (0.7814) | 45 (0.7541) | 1 (0.9945) | 1 (0.9945) | - | 4 (0.9781) |
| **4.D** | 41 (0.7760) | 42 (0.7705) | 36 (0.8033) | 36 (0.8033) | 43 (0.7650) | 43 (0.7650) | 44 (0.7596) | 47 (0.7432) | 40 (0.7814) | 40 (0.7814) | 40 (0.7814) | 42 (0.7705) | 4 (0.9781) | 4 (0.9781) | 4 (0.9781) | - |

**C**

| | 1.A | 1.B | 1.C | 1.D | 2.A | 2.B | 2.C | 2.D | 3.A | 3.B | 3.C | 3.D | 4.A | 4.B | 4.C | 4.D |
|---|---|---|---|---|---|---|---|---|---|---|---|---|---|---|---|---|
| **1.A** | - | 13 (0.7636) | 13 (0.7636) | 13 (0.7636) | 14 (0.7455) | 15 (0.7273) | 15 (0.7273) | 15 (0.7273) | 14 (0.7455) | 15 (0.7273) | 15 (0.7273) | 15 (0.7273) | 15 (0.7273) | 16 (0.7091) | 16 (0.7091) | 17 (0.6909) |
| **1.B** | 13 (0.7636) | - | 1 (0.9818) | 1 (0.9818) | 7 (0.8727) | 7 (0.8727) | 8 (0.8545) | 8 (0.8545) | 7 (0.8727) | 7 (0.8727) | 8 (0.8545) | 8 (0.8545) | 9 (0.8364) | 9 (0.8364) | 10 (0.8182) | 11 (0.8000) |
| **1.C** | 13 (0.7636) | 1 (0.9818) | - | 1 (0.9818) | 7 (0.8727) | 8 (0.8545) | 7 (0.8727) | 8 (0.8545) | 7 (0.8727) | 8 (0.8545) | 7 (0.8727) | 8 (0.8545) | 9 (0.8364) | 10 (0.8182) | 9 (0.8364) | 11 (0.8000) |
| **1.D** | 13 (0.7636) | 1 (0.9818) | 1 (0.9818) | - | 7 (0.8727) | 8 (0.8545) | 8 (0.8545) | 7 (0.8727) | 7 (0.8727) | 8 (0.8545) | 8 (0.8545) | 7 (0.8727) | 9 (0.8364) | 10 (0.8182) | 10 (0.8182) | 10 (0.8182) |
| **2.A** | 14 (0.7455) | 7 (0.8727) | 7 (0.8727) | 7 (0.8727) | - | 1 (0.9818) | 1 (0.9818) | 1 (0.9818) | 0 (1.0000) | 1 (0.9818) | 1 (0.9818) | 1 (0.9818) | 5 (0.9091) | 6 (0.8909) | 6 (0.8909) | 7 (0.8727) |
| **2.B** | 15 (0.7273) | 7 (0.8727) | 8 (0.8545) | 8 (0.8545) | 1 (0.9818) | - | 1 (0.9818) | 1 (0.9818) | 0 (1.0000) | 0 (1.0000) | 1 (0.9818) | 1 (0.9818) | 5 (0.9091) | 5 (0.9091) | 6 (0.8909) | 7 (0.8727) |
| **2.C** | 15 (0.7273) | 8 (0.8545) | 7 (0.8727) | 8 (0.8545) | 1 (0.9818) | 1 (0.9818) | - | 1 (0.9818) | 0 (1.0000) | 1 (0.9818) | 0 (1.0000) | 1 (0.9818) | 5 (0.9091) | 6 (0.8909) | 5 (0.9091) | 7 (0.8727) |
| **2.D** | 15 (0.7273) | 8 (0.8545) | 8 (0.8545) | 7 (0.8727) | 1 (0.9818) | 1 (0.9818) | 1 (0.9818) | - | 0 (1.0000) | 1 (0.9818) | 1 (0.9818) | 0 (1.0000) | 5 (0.9091) | 6 (0.8909) | 6 (0.8909) | 6 (0.8909) |
| **3.A** | 14 (0.7455) | 7 (0.8727) | 7 (0.8727) | 7 (0.8727) | 0 (1.0000) | 0 (1.0000) | 0 (1.0000) | 0 (1.0000) | - | 1 (0.9818) | 1 (0.9818) | 1 (0.9818) | 5 (0.9091) | 6 (0.8909) | 6 (0.8909) | 7 (0.8727) |
| **3.B** | 15 (0.7273) | 7 (0.8727) | 8 (0.8545) | 8 (0.8545) | 1 (0.9818) | 0 (1.0000) | 1 (0.9818) | 1 (0.9818) | 1 (0.9818) | - | 1 (0.9818) | 1 (0.9818) | 5 (0.9091) | 5 (0.9091) | 6 (0.8909) | 7 (0.8727) |
| **3.C** | 15 (0.7273) | 8 (0.8545) | 7 (0.8727) | 8 (0.8545) | 1 (0.9818) | 1 (0.9818) | 0 (1.0000) | 1 (0.9818) | 1 (0.9818) | 1 (0.9818) | - | 1 (0.9818) | 5 (0.9091) | 6 (0.8909) | 5 (0.9091) | 7 (0.8727) |
| **3.D** | 15 (0.7273) | 8 (0.8545) | 8 (0.8545) | 7 (0.8727) | 1 (0.9818) | 1 (0.9818) | 1 (0.9818) | 0 (1.0000) | 1 (0.9818) | 1 (0.9818) | 1 (0.9818) | - | 5 (0.9091) | 6 (0.8909) | 6 (0.8909) | 6 (0.8909) |
| **4.A** | 15 (0.7273) | 9 (0.8364) | 9 (0.8364) | 9 (0.8364) | 5 (0.9091) | 5 (0.9091) | 5 (0.9091) | 5 (0.9091) | 5 (0.9091) | 5 (0.9091) | 5 (0.9091) | 5 (0.9091) | - | 1 (0.9818) | 1 (0.9818) | 2 (0.9636) |
| **4.B** | 16 (0.7091) | 9 (0.8364) | 10 (0.8182) | 10 (0.8182) | 6 (0.8909) | 5 (0.9091) | 6 (0.8909) | 6 (0.8909) | 6 (0.8909) | 5 (0.9091) | 6 (0.8909) | 6 (0.8909) | 1 (0.9818) | - | 1 (0.9818) | 2 (0.9636) |
| **4.C** | 16 (0.7091) | 10 (0.8182) | 9 (0.8364) | 10 (0.8182) | 6 (0.8909) | 6 (0.8909) | 5 (0.9091) | 6 (0.8909) | 6 (0.8909) | 6 (0.8909) | 5 (0.9091) | 6 (0.8909) | 1 (0.9818) | 1 (0.9818) | - | 2 (0.9636) |
| **4.D** | 17 (0.6909) | 11 (0.8000) | 11 (0.8000) | 10 (0.8182) | 7 (0.8727) | 7 (0.8727) | 7 (0.8727) | 6 (0.8909) | 7 (0.8727) | 7 (0.8727) | 7 (0.8727) | 6 (0.8909) | 2 (0.9636) | 2 (0.9636) | 2 (0.9636) | - |

**Supplementary Table S5.** (**a**) Wildcard taxa identified in each parameter set of the sensitivity analysis of the representative dataset, with the number of additional nodes of the consensus gained by their exclusion (in parenthesis). Wildcard taxa decreasing the resolution of the consensus by 5 nodes or more are in bold. (**b**) Sum of wildcard taxa identified in each parameter set and the sum of additional nodes of the consensus gained by their exclusion. (**c**) Wildcard taxa identified in all parameters of the sensitivity analysis, the sum of parameter sets in which these taxa acted as wildcard taxa and the sum of additional nodes of the consensus gained by their exclusion.

**a**

| | A | B | C | D |
|---|---|---|---|---|
| **1** | Amhara (1)<br>Aari (1)<br>Alorese (1)<br>Dogon (1)<br>Egyptian (1)<br>Georgian (2)<br>Guarani, Toba, Wichi (1)<br>Mozabite (1) | Aari (1)<br>Amhara (1)<br>Andamanese (2)<br>Burusho (1)<br>Dogon (1)<br>Egyptian (1)<br>Guarani, Toba, Wichi (1)<br>Mozabite (1)<br>Yahgan, Tehuelche (3) | Ache (1)<br>**Andamanese (21)**<br>Armenian (1)<br>Egyptian (1)<br>Georgian (2)<br>**Malagasy (7)**<br>Singapore Malay (1)<br>Waiwai (1)<br>Yahgan, Tehuelche (3) | Ache (1)<br>**Andamanese (5)**<br>Armenian (1)<br>**Dayak Ngaju (10)**<br>Egyptian (1)<br>**Malagasy (20)**<br>Mapuche, Yahgan, Tehuelche (4)<br>Singapore Malay (1)<br>Waiwai (1) |
| **2** | Amhara (1)<br>Andamanese (3)<br>Dogon (2)<br>Guarani, Toba, Wichi (1)<br>Cherokee (1)<br>**Malagasy (23)** | Amhara (1)<br>Andamanese (2)<br>Dogon (2)<br>Guarani, Toba, Wichi (1)<br>Cherokee (1)<br>**Malagasy (23)** | Ache (1)<br>Cherokee (1)<br>Malagasy (3)<br>Nenets (1)<br>**Qatari (21)**<br>Waiwai (2) | Ache (1)<br>Amhara (1)<br>Andamanese (1)<br>Dogon (2)<br>Nenets (1)<br>**Qatari (21)**<br>Waiwai (2) |
| **3** | **Andamanese (7)**<br>Cherokee (1)<br>Guarani, Toba, Wichi (1)<br>**Malagasy (5)**<br>Nenets (1)<br>Orcadian (2) | **Andamanese (14)**<br>Cherokee (1)<br>Guarani, Toba, Wichi (1)<br>Malagasy (3)<br>Nenets (1)<br>Orcadian (2) | **Andamanese (14)**<br>Cherokee (1)<br>Guarani, Toba, Wichi (1)<br>Malagasy (3)<br>Nenets (1)<br>Orcadian (2) | Guarani, Toba, Wichi (1)<br>Andamanese (1)<br>**Malagasy (5)**<br>Nenets (1) |
| **4** | **Andamanese (7)**<br>Cherokee (1)<br>**Malagasy (9)**<br>Tibetan (2)<br>Toba, Wichi (1)<br>Yahgan (1) | **Andamanese (7)**<br>Cherokee (1)<br>**Malagasy (9)**<br>Tibetan (2)<br>Toba, Wichi (1)<br>Yahgan (1) | Amhara (1)<br>**Andamanese (7)**<br>Bidayuh (1)<br>Dogon (2)<br>Cherokee (1)<br>Mozabite (1)<br>Tibetan (2)<br>Toba, Wichi (1)<br>Yahgan (1) | Andamanese (1)<br>Cherokee (1)<br>Dayak Ngaju (1)<br>**Malagasy (9)**<br>Tibetan (2)<br>Toba, Wichi (1)<br>Yahgan (1) |

**b**

| | A | B | C | D | Sum |
|---|---|---|---|---|---|
| **1** | 8/9 | 9/12 | 9/38 | 9/44 | 35/103 |
| **2** | 6/31 | 6/30 | 6/29 | 7/29 | 25/119 |
| **3** | 6/17 | 6/22 | 6/22 | 4/9 | 22/70 |
| **4** | 6/21 | 6/21 | 9/17 | 7/16 | 28/75 |
| **Sum** | 26/78 | 27/85 | 30/106 | 27/98 | |

**c**

| | Sum of parameter sets | Sum of nodes |
|---|---|---|
| Aari | 2 | 4 |
| Ache | 4 | 4 |
| Alorese | 1 | 1 |
| Amhara | 6 | 6 |
| Andamanese | 14 | 92 |
| Armenian | 2 | 2 |
| Burusho | 1 | 1 |
| Dayak Ngaju | 2 | 11 |
| Dogon | 6 | 10 |
| Egyptian | 4 | 4 |
| Georgian | 2 | 4 |
| Guarani, Toba, Wichi | 8 | 8 |
| Cherokee | 10 | 10 |
| Malagasy | 12 | 119 |
| Mapuche, Yahgan, Tehuelche | 1 | 4 |
| Mozabite | 3 | 3 |
| Nenets | 6 | 6 |
| Orcadian | 3 | 6 |
| Qatari | 2 | 42 |
| Singapore_Malay | 2 | 2 |
| Tibetan | 4 | 8 |
| Toba, Wichi | 4 | 1 |
| Waiwai | 4 | 7 |
| Yahgan, Tehuelche | 2 | 6 |

**Supplementary Table S6.** List of characters included in the linguistic classification datasets. Characters that are phylogenetically informative for 186 taxa in the representative dataset are in black. Characters that are phylogenetically informative for 186 taxa in the representative dataset are in gray. Characters not used to constrain the topology of the language-constrained supertree (Fig. 3) are in red**.** (**a**) *Ethnologue* dataset. (**b**) Greenberg–Ruhlen dataset.

### a) *Ethnologue* dataset

[1] Khoisan
[2] Khoisan: Hatsa
[3] Khoisan: Sandawe
[4] Khoisan: Southern Africa
[5] Niger-Congo
[6] Niger-Congo: Mande
[7] Niger-Congo: Atlantic-Congo
[8] Niger-Congo: Atlantic-Congo: Atlantic
[9] Niger-Congo: Atlantic-Congo: Volta-Congo: Dogon
[10] Niger-Congo: Atlantic-Congo: Volta-Congo: Kwa
[11] Niger-Congo: Atlantic-Congo: Volta-Congo: North
[12] Niger-Congo: Atlantic-Congo: Volta-Congo: Benue-Congo
[13] Niger-Congo: Atlantic-Congo: Volta-Congo: Benue-Congo: Bantoid
[14] Niger-Congo: Atlantic-Congo: Volta-Congo: Benue-Congo: Nupoid
[15] Niger-Congo: Atlantic-Congo: Volta-Congo: Benue-Congo: Defoid
[16] Niger-Congo: Atlantic-Congo: Volta-Congo: Benue-Congo: Igboid
[17] Nilo-Saharan
[18] Afro-Asiatic
[19] Afro-Asiatic: Berber
[20] Afro-Asiatic: Chadic
[21] Afro-Asiatic: Cushtic
[22] Afro-Asiatic: Omotic
[23] Afro-Asiatic: Semitic
[24] Dravidian
[25] Kartvelian
[26] North Caucasian
[27] Indo-European
[28] Uralic
[29] Yukaghir
[30] Chukotko-Kamchatkan
[31] Eskimo-Aleut
[32] Altaic
[33] Altaic: Turkic
[34] Altaic: Mongolic

[35] Altaic: Tungusic
[36] Japonic
[37] Sino-Tibetan
[38] Sino-Tibetan: Chinese
[39] Sino-Tibetan: Tibeto-Burman
[40] Yeniseian
[41] Haida
[42] Eyak-Athabascan
[43] Eyak-Athabascan: Athabascan
[44] Hmong-Mien
[45] Tai-Kadai
[46] Austro-Asiatic
[47] Austro-Asiatic: Mon-Khmer
[48] Austro-Asiatic: Munda
[49] Austronesian
[50] Austronesian: Malayo-Polynesian
[51] Austronesian: Malayo-Polynesian: Oceanic
[52] Andamanese
[53] Yele-West New Britain
[54] East New Britain
[55] North Bougainville
[56] South Bougainville
[57] Central Solomons
[58] Torricelli
[59] Sepik
[60] Trans-New Guinea
[61] Australian
[62] Australian: Daly
[63] Australian: Gunwingguan
[64] Australian: Pama-Nyungan
[65] Algic
[66] Salish
[67] Yokutsan
[68] Muskogean
[69] Wakashan
[70] Siouan-Catawban
[71] Totonacan
[72] Pomoan
[73] Iroquoian
[74] Mayan
[75] Otomanguean
[76] Mixe-Zoquean
[77] Uto-Aztecan
[78] Chibchan

[79] Yanomaman
[80] Tarascan
[81] Paezan
[82] Barbacoan
[83] Chocoan
[84] Aymaran
[85] Quechuan
[86] Mapudungu
[87] Kaweskaran
[88] Chon
[89] Maipurean
[90] Guajiboan
[91] Jivaroan
[92] Chapacuran
[93] Salivan
[94] Mascoyan
[95] Arauan
[96] Zamucoan
[97] Chipaya-Uru
[98] Cariban
[99] Tupian
[100] Jean
[101] Tucanoan
[102] Puinavean
[103] Matacoan
[104] Guaykuruan
[105] Mosetenan

**b) Greenberg-Ruhlen dataset**

[1] Khoisan
[2] Niger-Kordofanian
[3] Nilo-Saharan
[4] Afro-Asiatic
[5] Dravidian
[6] Caucasian
[6] Kartvelian
[7] Indo-Hittite
[8] Uralic
[9] Chukchi-Kamchatkan
[10] Eskimo-Aleut
[11] Macro-Altaic

[12] Sino-Tibetan
[13] Na-Dene
[14] Austric
[15] Austric: Austroasiatic
[16] Austric: Miao-Yao
[17] Austric: Daic
[18] Austric: Austronesian
[19] Indo-Pacific
[20] Indo-Pacific: Kusunda
[21] Indo-Pacific: Andaman Islands
[22] Indo-Pacific: East Papuan
[23] Indo-Pacific: Toricelli
[24] Indo-Pacific: Sepik-Ramu
[25] Indo-Pacific: Trans-New Guinea
[26] Australian
[27] Australian: Gunwinyguan
[28] Australian: Wororan
[29] Australian: Pama-Nyungan
[30] Amerind
[31] Amerind: Almosan
[32] Amerind: Keresiouan
[33] Amerind: Penutian
[34] Amerind: Hokan
[35] Amerind: Central
[36] Amerind: Chibchan
[37] Amerind: Paezan
[38] Amerind: Andean
[39] Amerind: Equatorial
[40] Amerind: Macro-Tucanoan
[41] Amerind: Macro-Ge
[42] Amerind: Macro-Panoan
[43] Amerind: Macro-Carib

**Supplementary Table S7.** Consistency index (CI), retention index (RI), and normalized CI and RI values for linguistic groupings resulting from the optimization of the linguistic classification datasets on the topology of the purely genetic and combined supertree (based on parameter set 1.A of the sensitivity analysis). (**a**) *Ethnologue* dataset. (**b**) Greenberg–Ruhlen dataset. (**c**) Additional linguistic macrofamilies accepted from Ruhlen[1].

**a**

| | | c.i. | | r.i. | | normalized c.i. | | normalized r.i. | |
|---|---|---|---|---|---|---|---|---|---|
| **Linguistic grouping** | **N taxa** | **Genetic tree** | **Combined tree** | **Genetic tree** | **Combined tree** | **Genetic tree** | **Combined tree** | **Genetic tree** | **Combined tree** |
| Khoisan | 7 | 0.33333333 | 0.33333333 | 0.66666667 | 0.66666667 | 2.33333331 | 2.33333331 | 4.66666669 | 4.66666669 |
| Khoisan: Southern Africa | 5 | 1 | 1 | 1 | 1 | 5 | 5 | 5 | 5 |
| Niger-Congo | 10 | 0.16666667 | 0.16666667 | 0.44444444 | 0.44444444 | 1.6666667 | 1.6666667 | 4.4444444 | 4.4444444 |
| Niger-Congo: Atlantic-Congo | 9 | 0.16666667 | 0.16666667 | 0.375 | 0.375 | 1.50000003 | 1.50000003 | 3.375 | 3.375 |
| Niger-Congo: Atlantic-Congo: Volta-Congo: Benue-Congo | 6 | 0.33333333 | 0.33333333 | 0.6 | 0.6 | 1.99999998 | 1.99999998 | 3.6 | 3.6 |
| Niger-Congo: Atlantic-Congo: Volta-Congo: Benue-Congo: Bantoid | 6 | 0.33333333 | 0.33333333 | 0.6 | 0.6 | 1.99999998 | 1.99999998 | 3.6 | 3.6 |
| Nilo-Saharan | 5 | 0.25 | 0.25 | 0.25 | 0.25 | 1.25 | 1.25 | 1.25 | 1.25 |
| Afro-Asiatic | 28 | 0.11111111 | 0.16666667 | 0.7037037 | 0.81481481 | 3.11111108 | 4.66666676 | 19.7037036 | 22.81481468 |
| Afro-Asiatic: Berber | 2 | 0.5 | 0.5 | 0 | 0 | 1 | 1 | 0 | 0 |
| Afro-Asiatic: Chadic | 4 | 0.5 | 0.5 | 0.66666667 | 0.66666667 | 2 | 2 | 2.66666668 | 2.66666668 |
| Afro-Asiatic: Cushtic | 7 | 0.16666667 | 0.25 | 0.16666667 | 0.5 | 1.16666669 | 1.75 | 1.16666669 | 3.5 |
| Afro-Asiatic: Semitic | 14 | 0.16666667 | 0.2 | 0.61538462 | 0.69230769 | 2.33333338 | 2.8 | 8.61538468 | 9.69230766 |
| Dravidian | 4 | 0.5 | 0.5 | 0.66666667 | 0.66666667 | 2 | 2 | 2.66666668 | 2.66666668 |
| Indo-European | 22 | 0.11111111 | 0.125 | 0.61904762 | 0.66666667 | 2.44444442 | 2.75 | 13.6190476 4 | 14.66666674 |
| Uralic | 4 | 0.33333333 | 0.33333333 | 0.33333333 | 0.33333333 | 1.33333332 | 1.33333332 | 1.33333332 | 1.33333332 |
| Chukotko-Kamchatkan | 2 | 0.5 | 0.5 | 0 | 0 | 1 | 1 | 0 | 0 |
| Eskimo-Aleut | 2 | 0.5 | 0.5 | 0 | 0 | 1 | 1 | 0 | 0 |
| Altaic | 11 | 0.16666667 | 0.16666667 | 0.5 | 0.5 | 1.83333337 | 1.83333337 | 5.5 | 5.5 |
| Altaic: Turkic | 4 | 0.25 | 0.25 | 0 | 0 | 1 | 1 | 0 | 0 |
| Altaic: Mongolic | 3 | 0.33333333 | 0.33333333 | 0 | 0 | 0.99999999 | 0.99999999 | 0 | 0 |
| Altaic: Tungusic | 4 | 0.33333333 | 0.33333333 | 0.33333333 | 0.33333333 | 1.33333332 | 1.33333332 | 1.33333332 | 1.33333332 |
| Sino-Tibetan | 9 | 0.25 | 0.33333333 | 0.625 | 0.75 | 2.25 | 2.99999997 | 5.625 | 6.75 |
| Sino-Tibetan: Chinese | 4 | 1 | 1 | 1 | 1 | 4 | 4 | 4 | 4 |
| Sino-Tibetan: Tibeto-Burman | 5 | 0.33333333 | 0.33333333 | 0.5 | 0.5 | 1.66666665 | 1.66666665 | 2.5 | 2.5 |
| Eyak-Athabascan | 4 | 0.25 | 0.25 | 0 | 0 | 1 | 1 | 0 | 0 |
| Eyak-Athabascan: Athabascan | 3 | 0.33333333 | 0.33333333 | 0 | 0 | 0.99999999 | 0.99999999 | 0 | 0 |
| Hmong-Mien | 2 | 0.5 | 0.5 | 0 | 0 | 1 | 1 | 0 | 0 |
| Austro-Asiatic | 5 | 0.33333333 | 0.33333333 | 0.5 | 0.5 | 1.66666665 | 1.66666665 | 2.5 | 2.5 |

| | | | | | | | | | |
|---|---|---|---|---|---|---|---|---|---|
| Austro-Asiatic: Mon-Khmer | 5 | 0.33333333 | 0.33333333 | 0.5 | 0.5 | 1.66666665 | 1.66666665 | 2.5 | 2.5 |
| Austronesian | 28 | 0.2 | 0.25 | 0.85185185 | 0.88888889 | 5.6 | 7 | 23.8518518 | 24.88888892 |
| Austronesian: Malayo-Polynesian | 24 | 0.11111111 | 0.25 | 0.65217391 | 0.86956522 | 2.66666664 | 6 | 15.6521738 4 | 20.86956528 |
| Austronesian: Malayo-Polynesian: Oceanic | 5 | 0.5 | 0.33333333 | 0.75 | 0.5 | 2.5 | 1.66666665 | 3.75 | 2.5 |
| Australian | 3 | 0.5 | 0.33333333 | 0.5 | 0 | 1.5 | 0.99999999 | 1.5 | 0 |
| Algic | 2 | 1 | 1 | 1 | 1 | 2 | 2 | 2 | 2 |
| Chibchan | 2 | 1 | 1 | 1 | 1 | 2 | 2 | 2 | 2 |
| Maipurean | 2 | 1 | 1 | 1 | 1 | 2 | 2 | 2 | 2 |
| Tupian | 4 | 0.5 | 0.5 | 0.66666667 | 0.66666667 | 2 | 2 | 2.66666668 | 2.66666668 |

**b**

| | | c.i. | | r.i. | | rescaled c.i. | | rescaled r.i. | |
|---|---|---|---|---|---|---|---|---|---|
| **Linguistic grouping** | **N taxa** | **Genetic tree** | **Combined tree** | **Genetic tree** | **Combined tree** | **Genetic tree** | **Combined tree** | **Genetic tree** | **Combined tree** |
| Khoisan | 7 | 0.33333333 | 0.33333333 | 0.66666667 | 0.66666667 | 2.33333331 | 2.33333331 | 4.66666669 | 4.66666669 |
| Niger-Kordofanian | 10 | 0.16666667 | 0.16666667 | 0.44444444 | 0.44444444 | 1.6666667 | 1.6666667 | 4.4444444 | 4.4444444 |
| Nilo-Saharan | 5 | 0.25 | 0.25 | 0.25 | 0.25 | 1.25 | 1.25 | 1.25 | 1.25 |
| Afro-Asiatic | 28 | 0.11111111 | 0.16666667 | 0.7037037 | 0.81481481 | 3.11111108 | 4.66666676 | 19.7037036 | 22.81481468 |
| Dravidian | 4 | 0.5 | 0.5 | 0.66666667 | 0.66666667 | 2 | 2 | 2.66666668 | 2.66666668 |
| Indo-Hittite | 22 | 0.1 | 0.125 | 0.57142857 | 0.66666667 | 2.2 | 2.75 | 12.57142854 | 14.66666674 |
| Uralic | 5 | 0.25 | 0.25 | 0.25 | 0.25 | 1.25 | 1.25 | 1.25 | 1.25 |
| Chukchi-Kamchatkan | 2 | 0.5 | 0.5 | 0 | 0 | 1 | 1 | 0 | 0 |
| Eskimo-Aleut | 2 | 0.5 | 0.5 | 0 | 0 | 1 | 1 | 0 | 0 |
| Macro-Altaic | 13 | 0.14285714 | 0.14285714 | 0.5 | 0.5 | 1.85714282 | 1.85714282 | 6.5 | 6.5 |
| Sino-Tibetan | 9 | 0.25 | 0.25 | 0.625 | 0.625 | 2.25 | 2.25 | 5.625 | 5.625 |
| Na-Dene | 5 | 0.2 | 0.2 | 0 | 0 | 1 | 1 | 0 | 0 |
| Austric | 36 | 0.1 | 0.1 | 0.74285714 | 0.77142857 | 3.6 | 3.6 | 26.74285704 | 27.77142852 |
| Austric: Austroasiatic | 5 | 0.5 | 0.5 | 0.66666667 | 0.66666667 | 2.5 | 2.5 | 3.33333335 | 3.33333335 |
| Austric: Miao-Yao | 2 | 0.5 | 0.5 | 0 | 0 | 1 | 1 | 0 | 0 |
| Austric: Austronesian | 28 | 0.14285714 | 0.16666667 | 0.77777778 | 0.81481481 | 3.99999992 | 4.66666676 | 21.77777784 | 22.81481468 |
| Indo-Pacific | 3 | 0.33333333 | 0.33333333 | 0 | 0 | 0.99999999 | 0.99999999 | 0 | 0 |
| Australian | 3 | 0.5 | 0.33333333 | 0.5 | 0 | 1.5 | 0.99999999 | 1.5 | 0 |
| Amerind | 25 | 0.5 | 0.5 | 0.95833333 | 0.95833333 | 12.5 | 12.5 | 23.95833325 | 23.95833325 |
| Amerind: Almosan | 2 | 1 | 1 | 1 | 1 | 2 | 2 | 2 | 2 |
| Amerind: Keresiouan | 2 | 0.5 | 0.5 | 0 | 0 | 1 | 1 | 0 | 0 |
| Amerind: Central | 3 | 0.33333333 | 0.33333333 | 0 | 0 | 0.99999999 | 0.99999999 | 0 | 0 |
| Amerind: Chibchan | 2 | 1 | 1 | 1 | 1 | 2 | 2 | 2 | 2 |
| Amerind: Andean | 4 | 0.25 | 0.25 | 0.25 | 0.25 | 1 | 1 | 1 | 1 |
| Amerind: Equatorial | 6 | 0.33333333 | 0.33333333 | 0.6 | 0.6 | 1.99999998 | 1.99999998 | 3.6 | 3.6 |
| Amerind: Macro-Panoan | 2 | 1 | 1 | 1 | 1 | 2 | 2 | 2 | 2 |

c

| | | c.i. | | r.i. | | rescaled c.i. | | rescaled r.i. | |
|---|---|---|---|---|---|---|---|---|---|
| **Linguistic grouping** | **N taxa** | **Genetic tree** | **Combined tree** | **Genetic tree** | **Combined tree** | **Genetic tree** | **Combined tree** | **Genetic tree** | **Combined tree** |
| Eurasiatic | 46 | 0.05882353 | 0.0625 | 0.64444444 | 0.66666667 | 2.70588238 | 2.875 | 29.64444424 | 30.66666682 |
| Nostratic (incl. Afro-Asiatic and Dravidian) | 76 | 0.0625 | 0.07692308 | 0.8 | 0.84 | 4.75 | 5.84615408 | 60.8 | 63.84 |
| Na-Dene (incl. Yeniseian) | 6 | 0.16666667 | 0.16666667 | 0 | 0 | 1.00000002 | 1.00000002 | 0 | 0 |
| Dene-Caucasian | 36 | 0.07142857 | 0.07142857 | 0.27777778 | 0.27777778 | 2.57142852 | 2.57142852 | 10.00000008 | 10.00000008 |

**Supplementary Table S8.** Supertree dataset references.


1 Abbi, A. Is Great Andamanese genealogically and typologically distinct from Onge and Jarawa? *Language Sciences* **31**, 791-812, doi:10.1016/j.langsci.2008.02.002 (2009).

2 Abdulla, M. A. *et al.* Mapping human genetic diversity in Asia. *Science* **326**, 1541-1545, doi:10.1126/science.1177074 (2009).

3 Agrawal, S., Srivastava, S. K., Borkar, M. & Chaudhuri, T. K. Genetic affinities of north and northeastern populations of India: inference from HLA-based study. *Tissue Antigens* **72**, 120-130, doi:10.1111/j.1399-0039.2008.01083.x (2008).

4 Alfonso-Sanchez, M. A., Perez-Miranda, A. M. & Herrera, R. J. Autosomal microsatellite variability of the Arrernte people of Australia. *American Journal of Human Biology* **20**, 91-99, doi:10.1002/ajhb.20685 (2008).

5 Alkorta-Aranburu, G. *et al.* The genetic architecture of adaptations to high altitude in Ethiopia. *PLOS Genetics* **8**, doi:10.1371/journal.pgen.1003110 (2012).

6 Arnaiz-Villena, A. *et al.* HLA genes in Macedonians and the sub-Saharan origin of the Greeks. *Tissue Antigens* **57**, 118-127, doi:10.1034/j.1399-0039.2001.057002118.x (2001).

7 Arnaiz-Villena, A. *et al.* HLA genes in Uros from Titikaka Lake, Peru: origin and relationship with other Amerindians and worldwide populations. *International Journal of Immunogenetics* **36**, 159-167, doi:10.1111/j.1744-313X.2009.00841.x (2009).

8 Arnaiz-Villena, A. *et al.* The origin of Cretan populations as determined by characterization of HLA alleles. *Tissue Antigens* **53**, 213-226, doi:10.1034/j.1399-0039.1999.530301.x (1999).

9 Arnaiz-Villena, A. *et al.* HLA alleles and haplotypes in the Turkish population: relatedness to Kurds, Armenians and other Mediterraneans. *Tissue Antigens* **57**, 308-317, doi:10.1034/j.1399-0039.2001.057004308.x (2001).

10 Arnaiz-Villena, A. *et al.* Relatedness among Basques, Portuguese, Spaniards, and Algerians studied by HLA allelic frequencies and haplotypes. *Immunogenetics* **47**, 37-43, doi:10.1007/s002510050324 (1997).

11 Arnaiz-Villena, A. *et al.* HLA genes in the chuvashian population from European Russia: admixture of central European and Mediterranean populations. *Human Biology* **75**, 375-392, doi:10.1353/hub.2003.0040 (2003).

12 Arnaiz-Villena, A. *et al.* HLA genes in Mayos population from northeast Mexico. *Current Genomics* **8**, 466-475 (2007).

13 Arnaiz-Villena, A. *et al.* The origin of Amerindians and the peopling of the Americas according to HLA genes: admixture with Asian and Pacific people. *Current Genomics* **11**, 103-114, doi:10.2174/138920210790886862 (2010).

14 Arnaiz-Villena, A. *et al.* Origin of Aymaras from Bolivia and their relationship with other Amerindians according to HLA genes. *Tissue Antigens* **65**, 379-390, doi:10.1111/j.1399-0039.2005.00356.x (2005).

15 Arnaiz-Villena, A. *et al.* HLA genes in Mexican Mazatecans, the peopling of the Americas and the uniqueness of Amerindians. *Tissue Antigens* **56**, 405-416,

doi:10.1034/j.1399-0039.2000.560503.x (2000).
16 Atzmon, G. *et al.* Abraham's children in the genome era: major Jewish diaspora populations comprise distinct genetic clusters with shared Middle Eastern Ancestry. *American Journal of Human Genetics* **86**, 850-859, doi:10.1016/j.ajhg.2010.04.015 (2010).
17 Ayub, Q. *et al.* Reconstruction of human evolutionary tree using polymorphic autosomal microsatellites. *American Journal of Physical Anthropology* **122**, 259-268, doi:10.1002/ajpa.10234 (2003).
18 Balanovsky, O. *et al.* Parallel evolution of genes and languages in the Caucasus region. *Molecular Biology and Evolution* **28**, 2905-2920, doi:10.1093/molbev/msr126 (2011).
19 Bannai, M. *et al.* Analysis of HLA genes and haplotypes in Ainu (from Hokkaido, northern Japan) supports the premise that they descent from Upper Paleolithic populations of East Asia. *Tissue Antigens* **55**, 128-139, doi:10.1034/j.1399-0039.2000.550204.x (2000).
20 Barrantes, R. *et al.* Microevolution in lower Central America: genetic characterization of the Chibcha-speaking groups of Costa Rica and Panama, and a consensus taxonomy based on genetic and linguistic affinity. *American Journal of Human Genetics* **46**, 63-84 (1990).
21 Behar, D. M. *et al.* The genome-wide structure of the Jewish people. *Nature* **466**, 238-U112, doi:10.1038/nature09103 (2010).
22 Bharadwaj, U., Khan, F., Srivastava, S., Goel, H. & Agrawal, S. Phylogenetic applications of HLA class II loci. *International Journal of Human Genetics* **7**, 123 (2007).
23 Bieber, H., Bieber, S. W., Rodewald, A. & Barrantes, R. Microevolution and genetic affinities among six Amerindian tribes of lower Central America: comparative genetic study of serum proteins. *Human Biology* **68**, 929-953 (1996).
24 Biro, A. Z., Zalan, A., Volgyi, A. & Pamjav, H. A Y-Chromosomal Comparison of the Madjars (Kazakhstan) and the Magyars (Hungary). *American Journal of Physical Anthropology* **139**, 305-310, doi:10.1002/ajpa.20984 (2009).
25 Blagitko, N. *et al.* Polymorphism of the HLA-DRB1 locus in Colombian, Ecuadorian, and Chilean Amerinds. *Human Immunology* **54**, 74-81, doi:10.1016/s0198-8859(97)00005-0 (1997).
26 Bouckaert, R. *et al.* Mapping the origins and expansion of the Indo-European language family. *Science* **337**, 957-960, doi:10.1126/science.1219669 (2012).
27 Bruges-Armas, J. *et al.* HLA in the Azores Archipelago: possible presence of Mongoloid genes. *Tissue Antigens* **54**, 349-359, doi:10.1034/j.1399-0039.1999.540404.x (1999).
28 Bryc, K. *et al.* Genome-wide patterns of population structure and admixture in West Africans and African Americans. *Proceedings of the National Academy of Sciences of the United States of America* **107**, 786-791, doi:10.1073/pnas.0909559107 (2010).
29 Callegari-Jacques, S. M. *et al.* Autosome STRs in native South America—testing models of association with geography and language. *American Journal of Physical Anthropology* **145**, 371-381, doi:10.1002/ajpa.21505 (2011).

30 Cavalli-Sforza, L. L., Menozzi, P. & Piazza, A. *The History and Geography of Human Genes* (Princeton university press, 1994).
31 Comas, D. *et al.* Trading genes along the silk road: mtDNA sequences and the origin of central Asian populations. *American Journal of Human Genetics* **63**, 1824-1838, doi:10.1086/302133 (1998).
32 Corella, A., Bert, F., Perez-Perez, A., Gene, M. & Turbon, D. Mitochondrial DNA diversity of the Amerindian populations living in the Andean Piedmont of Bolivia: Chimane, Moseten, Aymara and Quechua. *Annals of Human Biology* **34**, 34-55, doi:10.1080/03014460601075819 (2007).
33 Crawford, M. H., Rubicz, R. C. & Zlojutro, M. Origins of Aleuts and the genetic structure of populations of the archipelago: molecular and archaeological perspectives. *Human Biology* **82**, 695-717 (2010).
34 Currie, T. E., Greenhill, S. J., Gray, R. D., Hasegawa, T. & Mace, R. Rise and fall of political complexity in island South-East Asia and the Pacific. *Nature* **467**, 801-804, doi:10.1038/nature09461 (2010).
35 Currie, T. E., Meade, A., Guillon, M. & Mace, R. Cultural phylogeography of the Bantu Languages of sub-Saharan Africa. *Proceedings of the Royal Society B-Biological Sciences* **280**, 8, doi:10.1098/rspb.2013.0695 (2013).
36 Deka, R. *et al.* Genetic variation at twentythree microsatellite loci in sixteen human populations. *Journal of Genetics* **78**, 99-121, doi:10.1007/bf02924561 (1999).
37 Delmestri, A. & Cristianini, N. Linguistic phylogenetic inference by PAM-like matrices. *Journal of Quantitative Linguistics* **19**, 95-120, doi:10.1080/09296174.2012.659001 (2012).
38 Dunn, M. Contact and phylogeny in Island Melanesia. *Lingua* **119**, 1664-1678, doi:10.1016/j.lingua.2007.10.026 (2009).
39 Dunn, M., Greenhill, S. J., Levinson, S. C. & Gray, R. D. Evolved structure of language shows lineage-specific trends in word-order universals. *Nature* **473**, 79-82, doi:10.1038/nature09923 (2011).
40 Dunn, M., Terrill, A., Reesink, G., Foley, R. A. & Levinson, S. C. Structural phylogenetics and the reconstruction of ancient language history. *Science* **309**, 2072-2075, doi:10.1126/science.1114615 (2005).
41 Edinur, H. A. *et al.* HLA polymorphism in six Malay subethnic groups in Malaysia. *Human Immunology* **70**, 518-526, doi:10.1016/j.humimm.2009.04.003 (2009).
42 Eshleman, J. A. *et al.* Mitochondrial DNA and prehistoric settlements: native migrations on the western edge of North America. *Human Biology* **76**, 55-75, doi:10.1353/hub.2004.0019 (2004).
43 Fadhlaoui-Zid, K. *et al.* Genome-wide and paternal diversity reveal a recent origin of human populations in North Africa. *PLOS ONE* **8**, doi:10.1371/journal.pone.0080293 (2013).
44 Farjadian, S. & Ghaderi, A. HLA class II similarities in Iranian Kurds and Azeris. *International Journal of Immunogenetics* **34**, 457-463, doi:10.1111/j.1744-313X.2007.00723.x (2007).
45 Farjadian, S., Moqadam, F. A. & Ghaderi, A. HLA class II gene polymorphism in Parsees and Zoroastrians of Iran. *International Journal of Immunogenetics* **33**, 185-

191, doi:10.1111/j.1744-313X.2006.00594.x (2006).
46 Farjadian, S. *et al.* Molecular analysis of HLA allele frequencies and haplotypes in Baloch of Iran compared with related populations of Pakistan. *Tissue Antigens* **64**, 581-587, doi:10.1111/j.1399-0039.2004.00302.x (2004).
47 Farjadian, S., Ota, M., Inoko, H. & Ghaderi, A. The genetic relationship among Iranian ethnic groups: an anthropological view based on HLA class II gene polymorphism. *Molecular Biology Reports* **36**, 1943-1950, doi:10.1007/s11033-008-9403-4 (2009).
48 Fedorova, S. A. *et al.* Autosomal and uniparental portraits of the native populations of Sakha (Yakutia): implications for the peopling of Northeast Eurasia. *BMC Evolutionary Biology* **13**, doi:10.1186/1471-2148-13-127 (2013).
49 Firasat, S. *et al.* Y-chromosomal evidence for a limited Greek contribution to the Pathan population of Pakistan. *European Journal of Human Genetics* **15**, 121-126, doi:10.1038/sj.ejhg.5201726 (2007).
50 Fortunato, L., Holden, C. & Mace, R. From bridewealth to dowry? *Human Nature* **17**, 355-376, doi:10.1007/s12110-006-1000-4 (2006).
51 Friedlaender, J. S. *et al.* The genetic structure of Pacific islanders. *PLOS Genetics* **4**, doi:10.1371/journal.pgen.0040019 (2008).
52 Fu, Q. M. *et al.* DNA analysis of an early modern human from Tianyuan Cave, China. *Proceedings of the National Academy of Sciences of the United States of America* **110**, 2223-2227, doi:10.1073/pnas.1221359110 (2013).
53 Garcia-Ortiz, J. E. *et al.* High-resolution molecular characterization of the HLA class I and class II in the Tarahumara Amerindian population. *Tissue Antigens* **68**, 135-146, doi:10.1111/j.1399-0039.2006.00636.x (2006).
54 Gaya-Vidal, M. *et al.* Autosomal and X chromosome Alu insertions in Bolivian Aymaras and Quechuas: two languages and one genetic pool. *American Journal of Human Biology* **22**, 154-162, doi:10.1002/ajhb.20967 (2010).
55 Geisler, H. & List, J.-M. in *Arbeitstagung der Indogermanischen Gesellschaft 2009: Die Ausbreitung des Indogermanischen Thesen aus Sprachwissenschaft, Archäologie und Genetik* (Würzburg, Germany, 2009).
56 Gomez-Casado, E. *et al.* HLA genes in Arabic-speaking Moroccans: close relatedness to Berbers and Iberians. *Tissue Antigens* **55**, 239-249, doi:10.1034/j.1399-0039.2000.550307.x (2000).
57 Gomez-Casado, E. *et al.* Origin of Mayans according to HLA genes and the uniqueness of Amerindians. *Tissue Antigens* **61**, 425-436, doi:10.1034/j.1399-0039.2003.00040.x (2003).
58 Gray, R. D. & Atkinson, Q. D. Language-tree divergence times support the Anatolian theory of Indo-European origin. *Nature* **426**, 435-439, doi:10.1038/nature02029 (2003).
59 Gray, R. D., Drummond, A. J. & Greenhill, S. J. Language phylogenies reveal expansion pulses and pauses in Pacific settlement. *Science* **323**, 479-483, doi:10.1126/science.1166858 (2009).
60 Gray, R. D. & Jordan, F. M. Language trees support the express-train sequence of Austronesian expansion. *Nature* **405**, 1052-1055, doi:10.1038/35016575 (2000).

61 Greenhill, S. J. & Gray, R. D. in *The Evolution of Cultural Diversity: A Phylogenetic Approach* (eds Ruth Mace, Clare J Holden, & Stephen Shennan) Ch. 3, 31-52 (UCL Press, 2005).
62 Hajjej, A. *et al.* The contribution of HLA class I and II alleles and haplotypes to the investigation of the evolutionary history of Tunisians. *Tissue Antigens* **68**, 153-162, doi:10.1111/j.1399-0039.2005.00622.x (2006).
63 Hatin, W. I. *et al.* Population genetic structure of peninsular Malaysia Malay sub-ethnic groups. *PLOS ONE* **6**, doi:10.1371/journal.pone.0018312 (2011).
64 Henn, B. M. *et al.* Genomic ancestry of North Africans supports back-to-Africa migrations. *PLOS Genetics* **8**, doi:10.1371/journal.pgen.1002397 (2012).
65 Henn, B. M. *et al.* Hunter-gatherer genomic diversity suggests a southern African origin for modern humans. *Proceedings of the National Academy of Sciences of the United States of America* **108**, 5154-5162, doi:10.1073/pnas.1017511108 (2011).
66 Hodoglugil, U. & Mahley, R. W. Turkish population structure and genetic ancestry reveal relatedness among Eurasian populations. *Annals of Human Genetics* **76**, 128-141, doi:10.1111/j.1469-1809.2011.00701.x (2012).
67 Holden, C. & Mace, R. Phylogenetic analysis of the evolution of lactose digestion in adults. *Human Biology* **69**, 605-628 (1997).
68 Holden, C. J. Bantu language trees reflect the spread of farming across sub-Saharan Africa: a maximum-parsimony analysis. *Proceedings of the Royal Society B-Biological Sciences* **269**, 793-799, doi:10.1098/rspb.2002.1955 (2002).
69 Holden, C. J. & Gray, R. D. in *Phylogenetic methods and the prehistory of languages* (eds Peter Forster & Colin Renfrew) 19-31 (McDonald Institute for Archaeological Research, 2006).
70 Holden, C. J., Meade, A. & Pagel, M. in *The Evolution of Cultural Diversity: A Phylogenetic Approach* (eds Ruth Mace, Clare J Holden, & Stephen Shennan) Ch. 4, 53-65 (UCL Press, 2005).
71 Honkola, T. *et al.* Cultural and climatic changes shape the evolutionary history of the Uralic languages. *Journal of Evolutionary Biology* **26**, 1244-1253, doi:10.1111/jeb.12107 (2013).
72 Horai, S. *et al.* mtDNA polymorphism in East Asian populations, with special reference to the peopling of Japan. *American Journal of Human Genetics* **59**, 579-590 (1996).
73 Hunley, K. *et al.* Genetic and linguistic coevolution in Northern Island Melanesia. *PLOS Genetics* **4**, doi:10.1371/journal.pgen.1000239 (2008).
74 Hunley, K. & Long, J. C. Gene flow across linguistic boundaries in Native North American populations. *Proceedings of the National Academy of Sciences of the United States of America* **102**, 1312-1317, doi:10.1073/pnas.0409301102 (2005).
75 Hunley, K. L., Cabana, G. S., Merriwether, D. A. & Long, J. C. A formal test of linguistic and genetic coevolution in Native Central and South America. *American Journal of Physical Anthropology* **132**, 622-631, doi:10.1002/ajpa.20542 (2007).
76 Huyghe, J. R. *et al.* A genome-wide analysis of population structure in the Finnish Saami with implications for genetic association studies. *European Journal of Human Genetics* **19**, 347-352, doi:10.1038/ejhg.2010.179 (2011).

77 Chaubey, G. & Endicott, P. The Andaman Islanders in a regional genetic context: Reexamining the evidence for an early peopling of the archipelago from South Asia. *Human Biology* **85**, 153-171 (2013).
78 Chaubey, G. *et al.* Population genetic structure in Indian Austroasiatic speakers: the role of landscape barriers and sex-specific admixture. *Molecular Biology and Evolution* **28**, 1013-1024, doi:10.1093/molbev/msq288 (2011).
79 Chen, Y. S. *et al.* mtDNA variation in the South African Kung and Khwe—and their genetic relationships to other African populations. *American Journal of Human Genetics* **66**, 1362-1383, doi:10.1086/302848 (2000).
80 Chhaya, S., Desai, S. & Saranath, D. HLA polymorphisms in Sindhi community in Mumbai, India. *International Journal of Immunogenetics* **37**, 373-377, doi:10.1111/j.1744-313X.2010.00936.x (2010).
81 Chu, C. C. *et al.* Diversity of HLA among Taiwan's indigenous tribes and the Ivatans in the Philippines. *Tissue Antigens* **58**, 9-18, doi:10.1034/j.1399-0039.2001.580102.x (2001).
82 Chu, J. Y. *et al.* Genetic relationship of populations in China. *Proceedings of the National Academy of Sciences of the United States of America* **95**, 11763-11768, doi:10.1073/pnas.95.20.11763 (1998).
83 Ingman, M. & Gyllensten, U. Mitochondrial genome variation and evolutionary history of Australian and New Guinean aborigines. *Genome Research* **13**, 1600-1606, doi:10.1101/gr.686603 (2003).
84 Ingman, M., Kaessmann, H., Paabo, S. & Gyllensten, U. Mitochondrial genome variation and the origin of modern humans. *Nature* **408**, 708-713 (2000).
85 Ivanova, M. *et al.* HLA polymorphism in Bulgarians defined by high-resolution typing methods in comparison with other populations. *Tissue Antigens* **60**, 496-504, doi:10.1034/j.1399-0039.2002.600605.x (2002).
86 Jarvis, J. P. *et al.* Patterns of ancestry, signatures of natural selection, and genetic association with stature in Western African pygmies. *PLOS Genetics* **8**, 299-313, doi:10.1371/journal.pgen.1002641 (2012).
87 Jin, F. *et al.* Population genetic studies on nine Aboriginal ethnic groups of Taiwan. I. Red cell enzyme systems. *Anthropological Science* **107**, 229-246 (1999).
88 Jinam, T. A. *et al.* Evolutionary history of continental Southeast Asians:“Early train” hypothesis based on genetic analysis of mitochondrial and autosomal DNA data. *Molecular Biology and Evolution* **29**, 3513-3527, doi:10.1093/molbev/mss169 (2012).
89 Jordan, F. M., Gray, R. D., Greenhill, S. J. & Mace, R. Matrilocal residence is ancestral in Austronesian societies. *Proceedings of the Royal Society B-Biological Sciences* **276**, 1957-1964, doi:10.1098/rspb.2009.0088 (2009).
90 Kang, L. L. *et al.* Genetic structures of the Tibetans and the Deng people in the Himalayas viewed from autosomal STRs. *Journal of Human Genetics* **55**, 270-277, doi:10.1038/jhg.2010.21 (2010).
91 Kharkov, V. N. *et al.* The origin of Yakuts: analysis of the Y-chromosome haplotypes. *Molecular Biology* **42**, 198-208, doi:10.1134/s0026893308020040 (2008).
92 Kitchen, A., Ehret, C., Assefa, S. & Mulligan, C. J. Bayesian phylogenetic analysis of Semitic languages identifies an Early Bronze Age origin of Semitic in the Near East.

*Proceedings of the Royal Society B: Biological Sciences* **276**, 2703-2710, doi:10.1098/rspb.2009.0408 (2009).
93 Knight, A. *et al.* African Y chromosome and mtDNA divergence provides insight into the history of click languages. *Current Biology* **13**, 464-473, doi:10.1016/s0960-9822(03)00130-1 (2003).
94 Kohlrausch, F. B. *et al.* Geography influences microsatellite polymorphism diversity in Amerindians. *American Journal of Physical Anthropology* **126**, 463-470, doi:10.1002/ajpa.20042 (2005).
95 Kopelman, N. M. *et al.* Genomic microsatellites identify shared Jewish ancestry intermediate between Middle Eastern and European populations. *BMC Genetics* **10**, doi:10.1186/1471-2156-10-80 (2009).
96 Krause, J. *et al.* The complete mitochondrial DNA genome of an unknown hominin from southern Siberia. *Nature* **464**, 894-897, doi:10.1038/nature08976 (2010).
97 Lachance, J. *et al.* Evolutionary history and adaptation from high-coverage whole-genome sequences of diverse African hunter-gatherers. *Cell* **150**, 457-469, doi:10.1016/j.cell.2012.07.009 (2012).
98 Levinson, S. C., Greenhill, S. J., Gray, R. D. & Dunn, M. Universal typological dependencies should be detectable in the history of language families. *Linguistic Typology* **15**, 509–534, doi:10.1515/LITY.2011.034 (2011).
99 Li, H., Cho, K., Kidd, J. R. & Kidd, K. K. Genetic landscape of Eurasia and “admixture” in Uyghurs. *American Journal of Human Genetics* **85**, 934-937, doi:10.1016/j.ajhg.2009.10.024 (2009).
100 Li, J. Z. *et al.* Worldwide human relationships inferred from genome-wide patterns of variation. *Science* **319**, 1100-1104, doi:10.1126/science.1153717 (2008).
101 Lin, M. *et al.* Heterogeneity of Taiwan's indigenous population: possible relation to prehistoric Mongoloid dispersals. *Tissue Antigens* **55**, 1-9, doi:10.1034/j.1399-0039.2000.550101.x (2000).
102 Listman, J. B. *et al.* Southeast Asian origins of five Hill Tribe populations and correlation of genetic to linguistic relationships inferred with genome-wide SNP data. *American Journal of Physical Anthropology* **144**, 300-308, doi:10.1002/ajpa.21408 (2011).
103 Magalhaes, T. R. *et al.* HGDP and HapMap snalysis by Ancestry Mapper reveals local and global population relationships. *PLOS ONE* **7**, doi:10.1371/journal.pone.0049438 (2012).
104 Martinez-Cruz, B. *et al.* In the heartland of Eurasia: the multilocus genetic landscape of Central Asian populations. *European Journal of Human Genetics* **19**, 216-223, doi:10.1038/ejhg.2010.153 (2011).
105 Martinez-Laso, J. *et al.* HLA in Jaidukama: an Amerindian secluded Colombian population with new haplotypes and Asian and Pacific-shared alleles. *Molecular Biology Reports* **38**, 3689-3701, doi:10.1007/s11033-010-0483-6 (2011).
106 Martinez-Laso, J. *et al.* HLA molecular markers in Tuvinians: a population with both Oriental and Caucasoid characteristics. *Annals of Human Genetics* **65**, 245-261, doi:10.1046/j.1469-1809.2001.6530245.x (2001).
107 Martinez-Laso, J. *et al.* Origin of Bolivian Quechua Amerindians: their relationship

with other American Indians and Asians according to HLA genes. *European Journal of Medical Genetics* **49**, 169-185, doi:10.1016/j.ejmg.2005.04.005 (2006).
108 McEvoy, B. P. *et al.* Whole-genome genetic diversity in a sample of Australians with deep Aboriginal ancestry. *American Journal of Human Genetics* **87**, 297-305, doi:10.1016/j.ajhg.2010.07.008 (2010).
109 Melton, T. *et al.* Polynesian genetic affinities with Southeast Asian populations as identified by mtDNA analysis. *American Journal of Human Genetics* **57**, 403-414 (1995).
110 Mendizabal, I. *et al.* Reconstructing the population history of European Romani from genome-wide data. *Current Biology* **22**, 2342-2349, doi:10.1016/j.cub.2012.10.039 (2012).
111 Mesa, N. R. *et al.* Autosomal, mtDNA, and Y-chromosome diversity in Amerinds: pre- and post-Columbian patterns of gene flow in South America. *American Journal of Human Genetics* **67**, 1277-1286 (2000).
112 Mirabal, S., Cadenas, A. M., Garcia-Bertrand, R. & Herrera, R. J. Ascertaining the role of Taiwan as a source for the Austronesian expansion. *American Journal of Physical Anthropology* **150**, 551-564, doi:10.1002/ajpa.22226 (2013).
113 Mohyuddin, A. *et al.* HLA polymorphism in six ethnic groups from Pakistan. *Tissue Antigens* **59**, 492-501, doi:10.1034/j.1399-0039.2002.590606.x (2002).
114 Morlote, D. M., Gayden, T., Arvind, P., Babu, A. & Herrera, R. J. The Soliga, an isolated tribe from Southern India: genetic diversity and phylogenetic affinities. *Journal of Human Genetics* **56**, 258-269, doi:10.1038/jhg.2010.173 (2011).
115 Moscoso, J. *et al.* HLA genes of Aleutian Islanders living between Alaska (USA) and Kamchatka (Russia) suggest a possible southern Siberia origin. *Molecular Immunology* **45**, 1018-1026, doi:10.1016/j.molimm.2007.07.024 (2008).
116 Moscoso, J. *et al.* HLA genes in Lamas Peruvian-Amazonian Amerindians. *Molecular Immunology* **43**, 1881-1889, doi:10.1016/j.molimm.2005.10.013 (2006).
117 Munkhbat, B. *et al.* Molecular analysis of HLA polymorphism in Khoton-Mongolians. *Tissue Antigens* **50**, 124-134, doi:10.1111/j.1399-0039.1997.tb02851.x (1997).
118 Muro, M. *et al.* HLA polymorphism in the Murcia population (Spain): in the cradle of the archaeologic Iberians. *Human Immunology* **62**, 910-921, doi:10.1016/s0198-8859(01)00290-7 (2001).
119 Nasidze, I. *et al.* Alu insertion polymorphisms and the genetic structure of human populations from the Caucasus. *European Journal of Human Genetics* **9**, 267-272, doi:10.1038/sj.ejhg.5200615 (2001).
120 Nasidze, I., Sarkisian, T., Kerimov, A. & Stoneking, M. Testing hypotheses of language replacement in the Caucasus: evidence from the Y-chromosome. *Human Genetics* **112**, 255-261, doi:10.1007/s00439-002-0874-4 (2003).
121 Nasidze, I. & Stoneking, M. Mitochondrial DNA variation and language replacements in the Caucasus. *Proceedings of the Royal Society B-Biological Sciences* **268**, 1197-1206 (2001).
122 Nei, M. & Roychoudhury, A. K. Evolutionary relationships of human populations on a global scale. *Molecular Biology and Evolution* **10**, 927-943 (1993).
123 Nowak, J. *et al.* Allele and extended haplotype polymorphism of HLA-A, -C, -B, -

DRB1 and -DQB1 loci in Polish population and genetic affinities to other populations. *Tissue Antigens* **71**, 193-205, doi:10.1111/j.1399-0039.2007.00991.x (2008).
124 Nurbakova, D., Rusakov, S. & Alexandrov, V. Quantifying uncertainty in phylogenetic studies of the Slavonic languages. *Procedia Computer Science* **18**, 2269-2277, doi:10.1016/j.procs.2013.05.398 (2013).
125 Omoto, K. & Saitou, N. Genetic origins of the Japanese: a partial support for the dual structure hypothesis. *American Journal of Physical Anthropology* **102**, 437-446 (1997).
126 Pagani, L. *et al.* Ethiopian genetic diversity reveals linguistic stratification and complex influences on the Ethiopian gene pool. *American Journal of Human Genetics* **91**, 83-96, doi:10.1016/j.ajhg.2012.05.015 (2012).
127 Pagel, M., Atkinson, Q. D. & Meade, A. Frequency of word-use predicts rates of lexical evolution throughout Indo-European history. *Nature* **449**, 717-U717, doi:10.1038/nature06176 (2007).
128 Pagel, M. & Meade, A. in *The Evolution of Cultural Diversity: A Phylogenetic Approach* (eds Ruth Mace, Clare J Holden, & Stephen Shennan) Ch. 13, 235-256 (UCL Press, 2005).
129 Parolin, M. L. & Carnese, F. R. HLA-DRB1 alleles in four Amerindian populations from Argentina and Paraguay. *Genetics and Molecular Biology* **32**, 212-219 (2009).
130 Patin, E. *et al.* Inferring the demographic history of African farmers and Pygmy hunter–gatherers using a multilocus resequencing data set. *PLOS Genetics* **5**, doi:10.1371/journal.pgen.1000448 (2009).
131 Pemberton, T. J., DeGiorgio, M. & Rosenberg, N. A. Population structure in a comprehensive genomic data set on human microsatellite variation. *G3-Genes Genomes Genetics* **3**, 891-907, doi:10.1534/g3.113.005728 (2013).
132 Petersen, D. C. *et al.* Complex patterns of genomic admixture within southern Africa. *PLOS Genetics* **9**, doi:10.1371/journal.pgen.1003309 (2013).
133 Pickrell, J. K. *et al.* The genetic prehistory of southern Africa. *Nature Communications* **3**, doi:10.1038/ncomms2140 (2012).
134 Pickrell, J. K. & Pritchard, J. K. Inference of population splits and mixtures from genome-wide allele frequency data. *PLOS Genetics* **8**, doi:10.1371/journal.pgen.1002967 (2012).
135 Pickrell, J. K. & Pritchard, J. K. *Extending TreeMix to microsatellite data.* Published by the authors (2012).
136 Pugach, I., Delfin, F., Gunnarsdottir, E., Kayser, M. & Stoneking, M. Genome-wide data substantiate Holocene gene flow from India to Australia. *Proceedings of the National Academy of Sciences of the United States of America* **110**, 1803-1808, doi:10.1073/pnas.1211927110 (2013).
137 Rama, T. & Singh, A. K. in *International Conference RANLP 2009* 355–359 (Borovets, Bulgaria, 2009).
138 Rasmussen, M. *et al.* An Aboriginal Australian genome reveals separate human dispersals into Asia. *Science* **334**, 94-98, doi:10.1126/science.1211177 (2011).
139 Rasmussen, M. *et al.* Ancient human genome sequence of an extinct Palaeo-Eskimo. *Nature* **463**, 757-762, doi:10.1038/nature08835 (2010).

140 Redd, A. J. & Stoneking, M. Peopling of Sahul: mtDNA variation in Aboriginal Australian and Papua New Guinean populations. *American Journal of Human Genetics* **65**, 808-828, doi:10.1086/302533 (1999).
141 Reesink, G., Singer, R. & Dunn, M. Explaining the linguistic diversity of Sahul using population models. *PLOS Biology* **7**, doi:10.1371/journal.pbio.1000241 (2009).
142 Regueiro, M. *et al.* Austronesian genetic signature in East African Madagascar and Polynesia. *Journal of Human Genetics* **53**, 106-120, doi:10.1007/s10038-007-0224-4 (2008).
143 Reich, D. *et al.* Reconstructing Native American population history. *Nature* **488**, 370-+, doi:10.1038/nature11258 (2012).
144 Rexova, K., Bastin, Y. & Frynta, D. Cladistic analysis of Bantu languages: a new tree based on combined lexical and grammatical data. *Naturwissenschaften* **93**, 189-194, doi:10.1007/s00114-006-0088-z (2006).
145 Rey, D. *et al.* HLA genetic profile of Mapuche (Araucanian) Amerindians from Chile. *Molecular Biology Reports* **40**, 4257-4267, doi:10.1007/s11033-013-2509-3 (2013).
146 Rolf, B. *et al.* Microsatellite profiles reveal an unexpected genetic relationship between Asian populations. *Human Genetics* **102**, 647-652, doi:10.1007/s004390050757 (1998).
147 Rosenberg, N. A. *et al.* Low levels of genetic divergence across geographically and linguistically diverse populations from India. *PLOS Genetics* **2**, 2052-2061, doi:10.1371/journal.pgen.0020215 (2006).
148 Rosenberg, N. A. *et al.* Genetic structure of human populations. *Science* **298**, 2381-2385, doi:10.1126/science.1078311 (2002).
149 Rubicz, R. *et al.* Genetic structure of native circumpolar populations based on autosomal, mitochondrial, and Y chromosome DNA markers. *American Journal of Physical Anthropology* **143**, 62-74, doi:10.1002/ajpa.21290 (2010).
150 Ruiz-Linares, A. *et al.* Microsatellites provide evidence for Y chromosome diversity among the founders of the New World. *Proceedings of the National Academy of Sciences of the United States of America* **96**, 6312-6317, doi:10.1073/pnas.96.11.6312 (1999).
151 Salmela, E. *et al.* Swedish population substructure revealed by genome-wide single nucleotide polymorphism data. *PLOS ONE* **6**, doi:10.1371/journal.pone.0016747 (2011).
152 Sanchez-Velasco, P., de Diego, J. E., Paz-Miguel, J. E., Ocejo-Vinyals, G. & Leyva-Cobian, F. HLA-DR, DQ nucleotide sequence polymorphisms in the Pasiegos (Pas valleys, Northern Spain) and comparison of the allelic and haplotypic frequencies with those of other European populations. *Tissue Antigens* **53**, 65-73, doi:10.1034/j.1399-0039.1999.530107.x (1999).
153 Sanchez-Velasco, P. *et al.* HLA alleles in isolated populations from North Spain: origin of the Basques and the ancient Iberians. *Tissue Antigens* **61**, 384-392, doi:10.1034/j.1399-0039.2003.00041.x (2003).
154 Sanchez-Velasco, P., Karadsheh, N. S., Garcia-Martin, A., de Alegria, C. R. & Leyva-Cobian, F. Molecular analysis of HLA allelic frequencies and haplotypes in Jordanians and comparison with other related populations. *Human Immunology* **62**, 901-909,

doi:10.1016/s0198-8859(01)00289-0 (2001).
155 Sanchez-Velasco, P. & Leyva-Cobian, F. The HLA class I and class II allele frequencies studied at the DNA level in the Svanetian population (Upper Caucasus) and their relationships to Western European populations. *Tissue Antigens* **58**, 223-233, doi:10.1034/j.1399-0039.2001.580402.x (2001).
156 Sellen, D. W. & Mace, R. Fertility and mode of subsistence: a phylogenetic analysis. *Current Anthropology* **38**, 878-889, doi:10.1086/204677 (1997).
157 Serva, M. & Petroni, F. Indo-European languages tree by Levenshtein distance. *EPL (Europhysics Letters)* **81**, doi:10.1209/0295-5075/81/68005 (2008).
158 Shaw, C. K., Chen, L. L., Lee, A. & Lee, T. D. Distribution of HLA gene and haplotype frequencies in Taiwan: a comparative study among Min-nan, Hakka, Aborigines and Mainland Chinese. *Tissue Antigens* **53**, 51-64, doi:10.1034/j.1399-0039.1999.530106.x (1999).
159 Schlebusch, C. M., Lombard, M. & Soodyall, H. MtDNA control region variation affirms diversity and deep sub-structure in populations from southern Africa. *BMC Evolutionary Biology* **13**, doi:10.1186/1471-2148-13-56 (2013).
160 Schlebusch, C. M. *et al.* Genomic variation in seven Khoe-San groups reveals adaptation and complex African history. *Science* **338**, 374-379, doi:10.1126/science.1227721 (2012).
161 Sikora, M., Laayouni, H., Calafell, F., Comas, D. & Bertranpetit, J. A genomic analysis identifies a novel component in the genetic structure of sub-Saharan African populations. *European Journal of Human Genetics* **19**, 84-88, doi:10.1038/ejhg.2010.141 (2011).
162 Starikovskaya, E. B. *et al.* Mitochondrial DNA diversity in indigenous populations of the southern extent of Siberia, and the origins of Native American haplogroups. *Annals of Human Genetics* **69**, 67-89, doi:10.1046/j.1529-8817.2003.00127.x (2005).
163 Sulcebe, G. *et al.* HLA allele and haplotype frequencies in the Albanian population and their relationship with the other European populations. *International Journal of Immunogenetics* **36**, 337-343, doi:10.1111/j.1744-313X.2009.00868.x (2009).
164 Suslova, T. A. *et al.* HLA gene and haplotype frequencies in Russians, Bashkirs and Tatars, living in the Chelyabinsk Region (Russian South Urals). *International Journal of Immunogenetics* **39**, 394-408, doi:10.1111/j.1744-313X.2012.01117.x (2012).
165 Tajima, A. *et al.* Genetic origins of the Ainu inferred from combined DNA analyses of maternal and paternal lineages. *Journal of Human Genetics* **49**, 187-193, doi:10.1007/s10038-004-0131-x (2004).
166 Tajima, A. *et al.* Mitochondrial DNA polymorphisms in nine aboriginal groups of Taiwan: implications for the population history of aboriginal Taiwanese. *Human Genetics* **113**, 24-33, doi:10.1007/s00439-003-0945-1 (2003).
167 Thomas, R., Nair, S. B. & Banerjee, M. HLA-B and HLA-C alleles and haplotypes in the Dravidian tribal populations of southern India. *Tissue Antigens* **64**, 58-65, doi:10.1111/j.1399-0039.2004.00244.x (2004).
168 Tishkoff, S. A. *et al.* History of click-speaking populations of Africa inferred from mtDNA and Y chromosome genetic variation. *Molecular Biology and Evolution* **24**, 2180-2195, doi:10.1093/molbev/msm155 (2007).

169 Tishkoff, S. A. *et al.* The Genetic structure and history of Africans and African Americans. *Science* **324**, 1035-1044, doi:10.1126/science.1172257 (2009).
170 Torroni, A. *et al.* mtDNA and Y-chromosome polymorphisms in four Native American populations from southern Mexico. *American Journal of Human Genetics* **54**, 303-318 (1994).
171 Tsuneto, L. T. *et al.* HLA class II diversity in seven Amerindian populations. Clues about the origins of the Ache. *Tissue Antigens* **62**, 512-526, doi:10.1046/j.1399-0039.2003.00139.x (2003).
172 Uinuk-ool, T. S., Takezaki, N., Derbeneva, O. A., Volodko, N. V. & Sukernik, R. I. Variation of HLA class II genes in the Nganasan and Ket, two aboriginal Siberian populations. *European Journal of Immunogenetics* **31**, 43-51, doi:10.1111/j.1365-2370.2004.00443.x (2004).
173 Uinuk-ool, T. S., Takezaki, N., Sukernik, R. I., Nagl, S. & Klein, J. Origin and affinities of indigenous Siberian populations as revealed by HLA class II gene frequencies. *Human Genetics* **110**, 209-226, doi:10.1007/s00439-001-0668-0 (2002).
174 Usme-Romero, S., Alonso, M., Hernandez-Cuervo, H., Yunis, E. J. & Yunis, J. J. Genetic differences between Chibcha and Non-Chibcha speaking tribes based on mitochondrial DNA (mtDNA) haplogroups from 21 Amerindian tribes from Colombia. *Genetics and Molecular Biology* **36**, 149-157 (2013).
175 Vargas-Alarcon, G. *et al.* HLA genes in Mexican Teeneks: HLA genetic relationship with other worldwide populations. *Molecular Immunology* **43**, 790-799, doi:10.1016/j.molimm.2005.07.017 (2006).
176 Vargas-Alarcon, G. *et al.* Origin of Mexican Nahuas (Aztecs) according to HLA genes and their relationships with worldwide populations. *Molecular Immunology* **44**, 747-755, doi:10.1016/j.molimm.2006.04.014 (2007).
177 Veeramah, K. R. *et al.* An early divergence of KhoeSan ancestors from those of other modern humans is supported by an ABC-based analysis of autosomal resequencing data. *Molecular Biology and Evolution* **29**, 617-630, doi:10.1093/molbev/msr212 (2012).
178 Verdu, P. *et al.* Origins and genetic diversity of pygmy hunter-gatherers from Western Central Africa. *Current Biology* **19**, 312-318, doi:10.1016/j.cub.2008.12.049 (2009).
179 Walker, R. S., Hill, K. R., Flinn, M. V. & Ellsworth, R. M. Evolutionary history of hunter-gatherer marriage practices. *PLOS ONE* **6**, doi:10.1371/journal.pone.0019066 (2011).
180 Walker, R. S. & Ribeiro, L. A. Bayesian phylogeography of the Arawak expansion in lowland South America. *Proceedings of the Royal Society B-Biological Sciences* **278**, 2562-2567, doi:10.1098/rspb.2010.2579 (2011).
181 Walker, R. S., Wichmann, S., Mailund, T. & Atkisson, C. J. Cultural phylogenetics of the Tupi language family in lowland South America. *PLOS ONE* **7**, doi:10.1371/journal.pone.0035025 (2012).
182 Walsh, S. J., Mitchell, R. J., Watson, N. & Buckleton, J. S. A comprehensive analysis of microsatellite diversity in Aboriginal Australians. *Journal of Human Genetics* **52**, 712-728, doi:10.1007/s10038-007-0172-z (2007).
183 Wang, B. B. *et al.* On the origin of Tibetans and their genetic basis in adapting high-

altitude environments. *PLOS ONE* **6**, doi:10.1371/journal.pone.0017002 (2011).
184 Wang, S. *et al.* Genetic variation and population structure in Native Americans. *PLOS Genetics* **3**, 2049-2067, doi:10.1371/journal.pgen.0030185 (2007).
185 Wangkumhang, P. *et al.* Insight into the peopling of mainland southeast Asia from Thai population genetic structure. *PLOS ONE* **8**, e79522 (2013).
186 Watkins, W. S. *et al.* Patterns of ancestral human diversity: An analysis of Alu-insertion and restriction-site polymorphisms. *American Journal of Human Genetics* **68**, 738-752, doi:10.1086/318793 (2001).
187 Watkins, W. S. *et al.* Genetic variation among world populations: inferences from 100 Alu insertion polymorphisms. *Genome Research* **13**, 1607-1618, doi:10.1101/gr.894603 (2003).
188 Wells, R. S. *et al.* The Eurasian Heartland: A continental perspective on Y-chromosome diversity. *Proceedings of the National Academy of Sciences of the United States of America* **98**, 10244-10249, doi:10.1073/pnas.171305098 (2001).
189 Wollstein, A. *et al.* Demographic history of Oceania inferred from genome-wide data. *Current Biology* **20**, 1983-1992, doi:10.1016/j.cub.2010.10.040 (2010).
190 Xing, J. *et al.* Genomic analysis of natural selection and phenotypic variation in high-altitude Mongolians. *PLOS Genetics* **9**, doi:10.1371/journal.pgen.1003634 (2013).
191 Xing, J. C. *et al.* Toward a more uniform sampling of human genetic diversity: A survey of worldwide populations by high-density genotyping. *Genomics* **96**, 199-210, doi:10.1016/j.ygeno.2010.07.004 (2010).
192 Xing, J. C. *et al.* Fine-scaled human genetic structure revealed by SNP microarrays. *Genome Research* **19**, 815-825, doi:10.1101/gr.085589.108 (2009).
193 Xu, S. H. *et al.* Genetic evidence supports linguistic affinity of Mlabri - a hunter-gatherer group in Thailand. *BMC Genetics* **11**, doi:10.1186/1471-2156-11-18 (2010).
194 Xu, S. H. *et al.* Genetic dating indicates that the Asian-Papuan admixture through Eastern Indonesia corresponds to the Austronesian expansion. *Proceedings of the National Academy of Sciences of the United States of America* **109**, 4574-4579, doi:10.1073/pnas.1118892109 (2012).
195 Yuasa, I. *et al.* A hypervariable STR polymorphism in the CFI gene: southern origin of East Asian-specific group H alleles. *Legal Medicine* **15**, 239-243, doi:10.1016/j.legalmed.2013.04.001 (2013).
196 Yuasa, I. *et al.* Population genetic studies on nine aboriginal ethnic groups of Taiwan II. Serum protein systems. *Anthropological Science* **109**, 257-273 (2001).
197 Yuliwulandari, R. *et al.* Polymorphisms of HLA genes in Western Javanese (Indonesia): close affinities to Southeast Asian populations. *Tissue Antigens* **73**, 46-53, doi:10.1111/j.1399-0039.2008.01178.x (2009).
198 Yunis, J. J., Yunis, E. J. & Yunis, E. Genetic relationship of the Guambino, Paez, and Ingano Amerindians of southwest Colombia using major histocompatibility complex class II haplotypes and blood groups. *Human Immunology* **62**, 970-978, doi:10.1016/s0198-8859(01)00295-6 (2001).
199 Yunis, J. J., Yunis, E. J. & Yunis, E. MHC Class II haplotypes of Colombian Amerindian tribes. *Genetics and Molecular Biology* **36**, 158-166 (2013).
200 Zhu, B. F. *et al.* Distributions of HLA-A and -B alleles and haplotypes in the Yi ethnic

minority of Yunnan, China: relationship to other populations. *Journal of Zhejiang University-Science B* **11**, 127-135, doi:10.1631/jzus.B0900232 (2010).

## SUPPLEMENTARY FIGURES

**Supplementary Figure S1.** Semistrict consensus supertree based on parameter set 1.A of the sensitivity analysis of the representative dataset. A symbol "|O|" indicates clades which would be collapsed into polytomies in a strict consensus supertree.

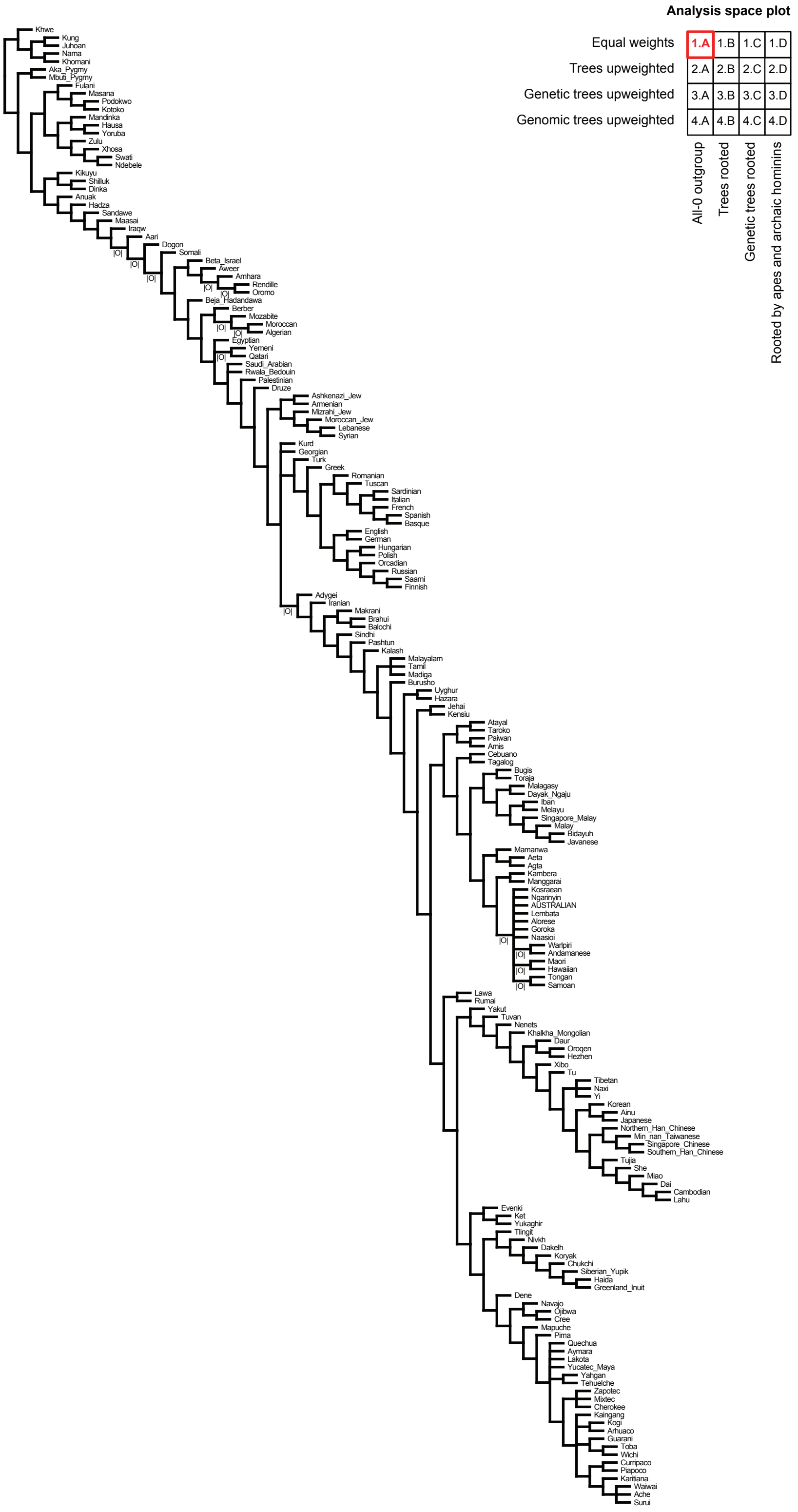

**Supplementary Figure S2.** Semistrict consensus supertree based on parameter set 1.B of the sensitivity analysis of the representative dataset. A symbol “|O|” indicates clades which would be collapsed into polytomies in a strict consensus supertree.

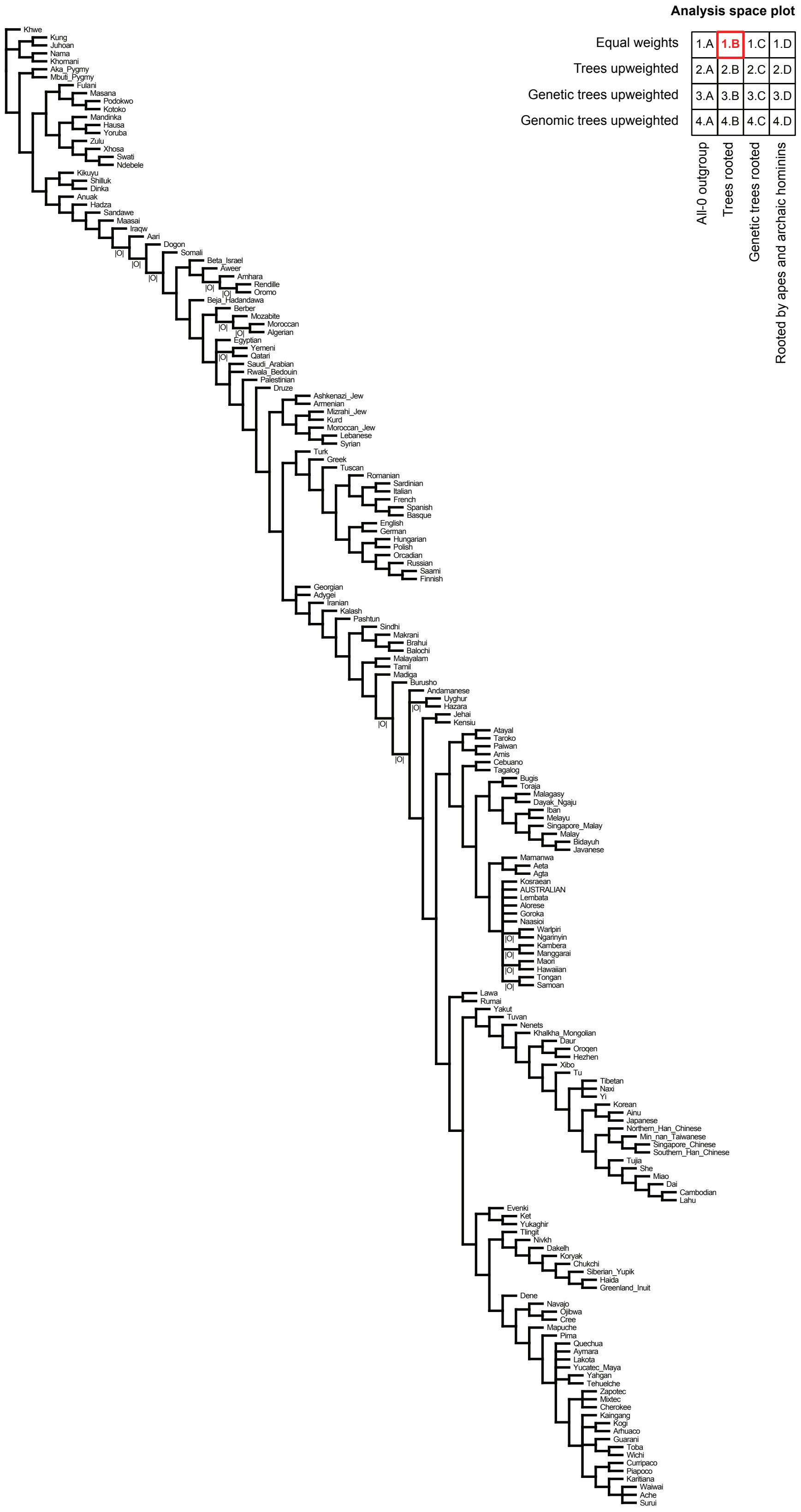

**Supplementary Figure S3.** Semistrict consensus supertree based on parameter set 1.C of the sensitivity analysis of the representative dataset. A symbol “|O|” indicates clades which would be collapsed into polytomies in a strict consensus supertree.

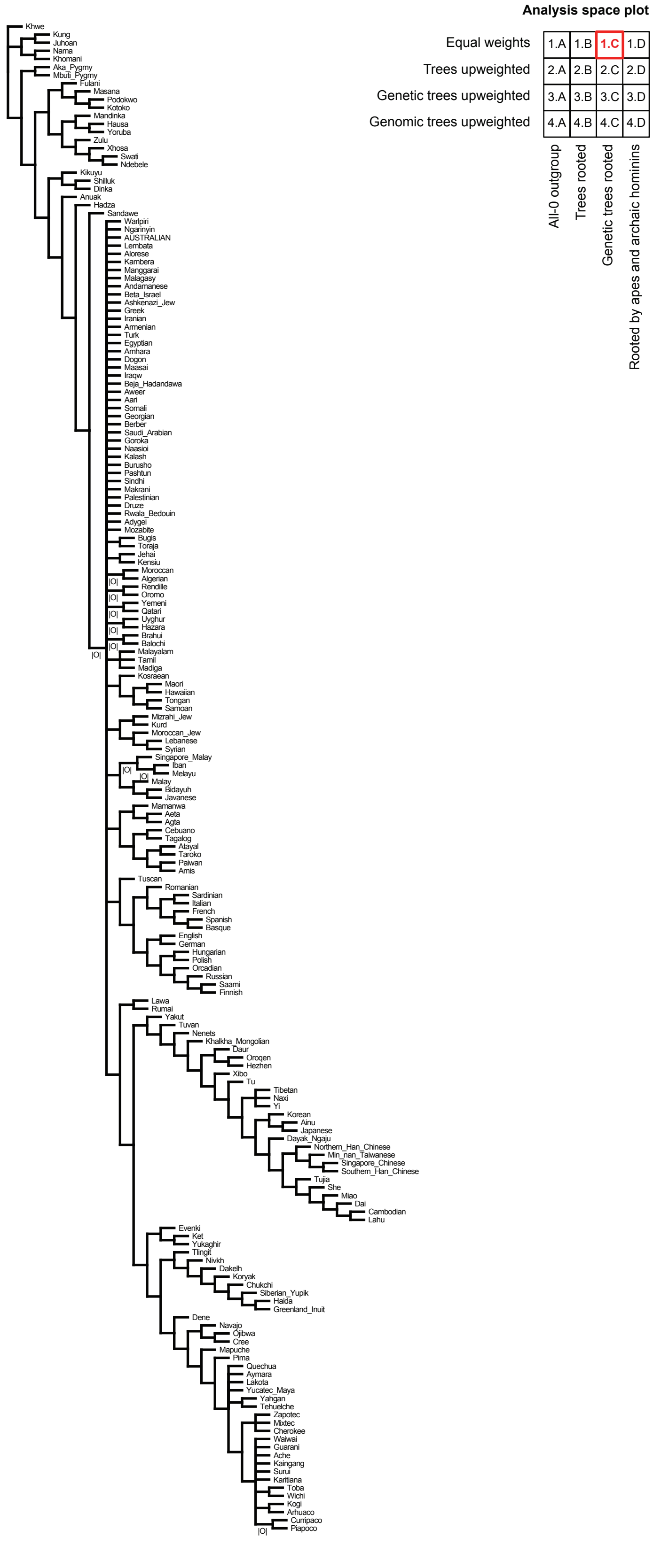

**Supplementary Figure S4.** Semistrict consensus supertree based on parameter set 1.D of the sensitivity analysis of the representative dataset. A symbol "|O|" indicates clades which would be collapsed into polytomies in a strict consensus supertree.

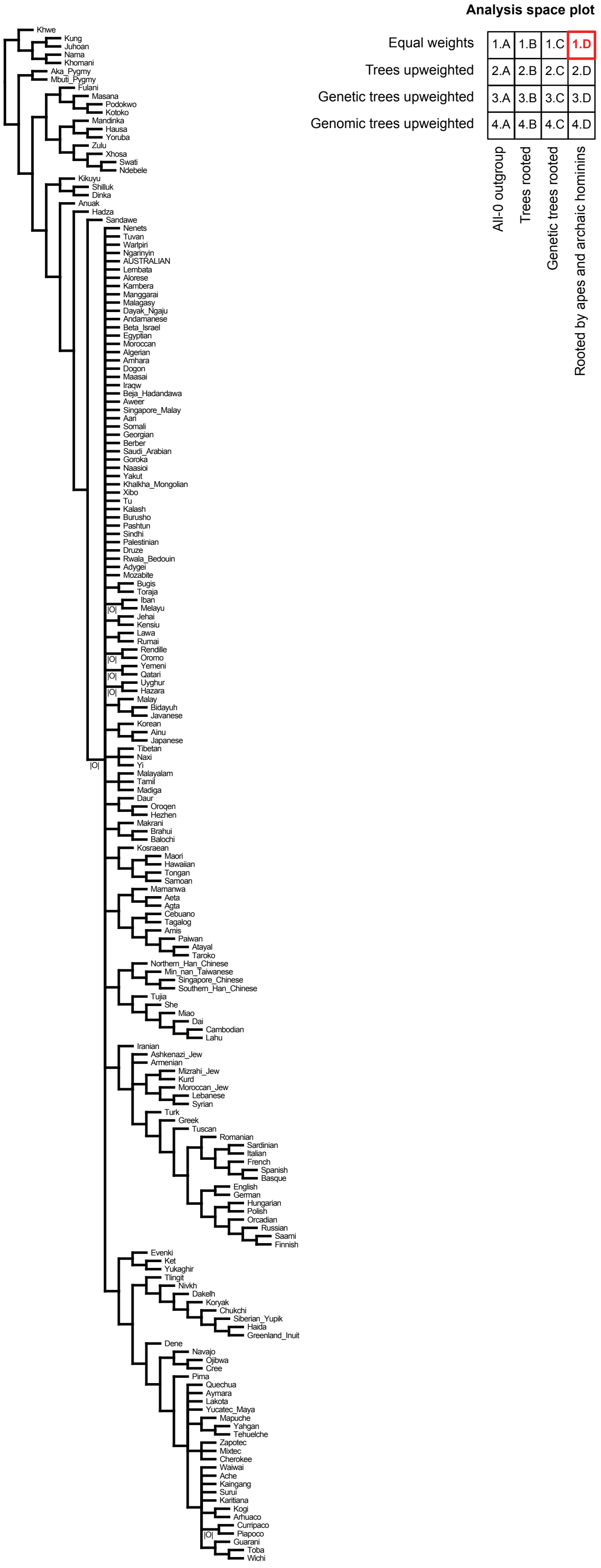

**Supplementary Figure S5.** Semistrict consensus supertree based on parameter set 2.A of the sensitivity analysis of the representative dataset. A symbol "|O|" indicates clades which would be collapsed into polytomies in a strict consensus supertree.

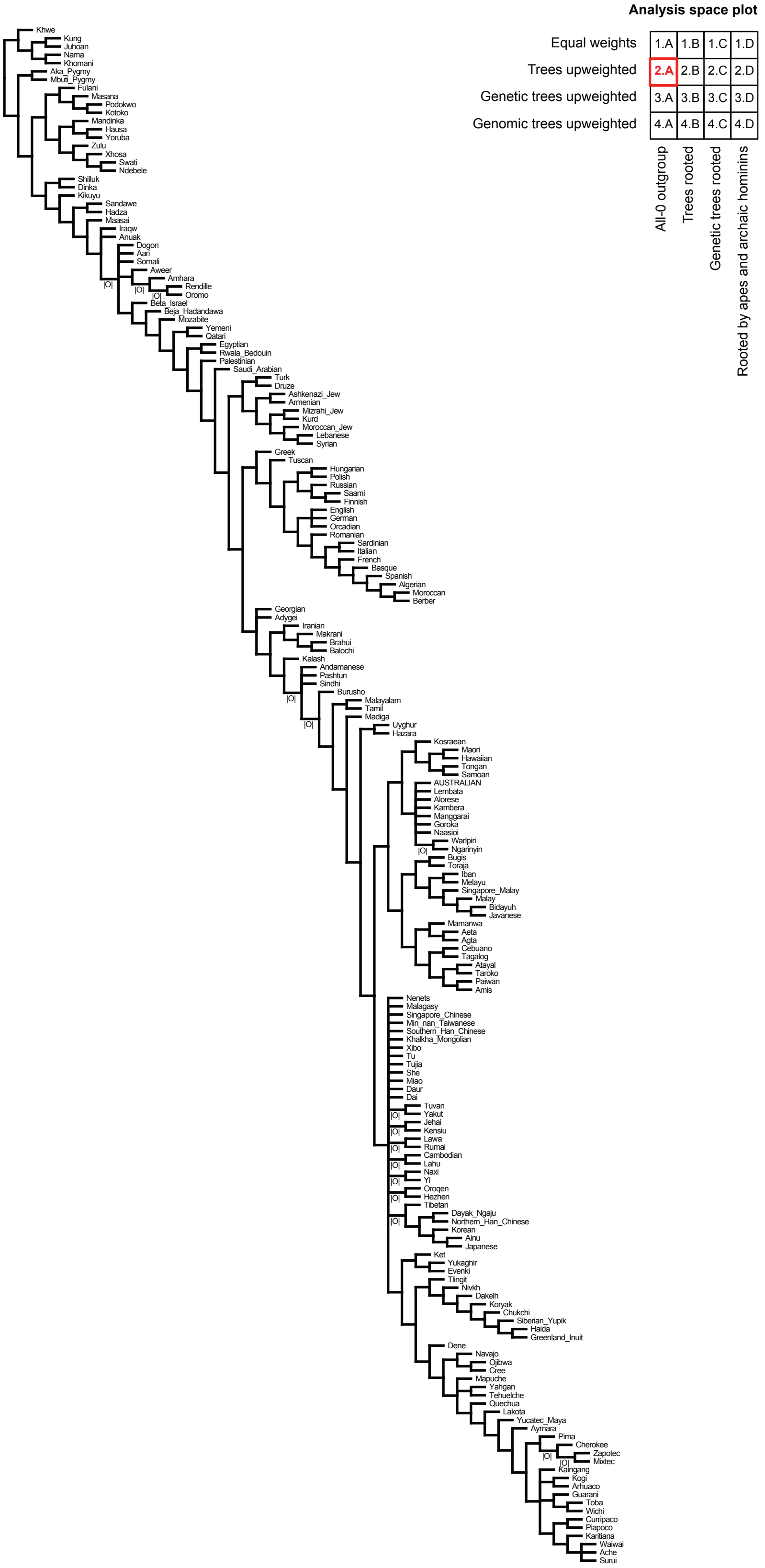

**Supplementary Figure S6.** Semistrict consensus supertree based on parameter set 2.B of the sensitivity analysis of the representative dataset. A symbol "|O|" indicates clades which would be collapsed into polytomies in a strict consensus supertree.

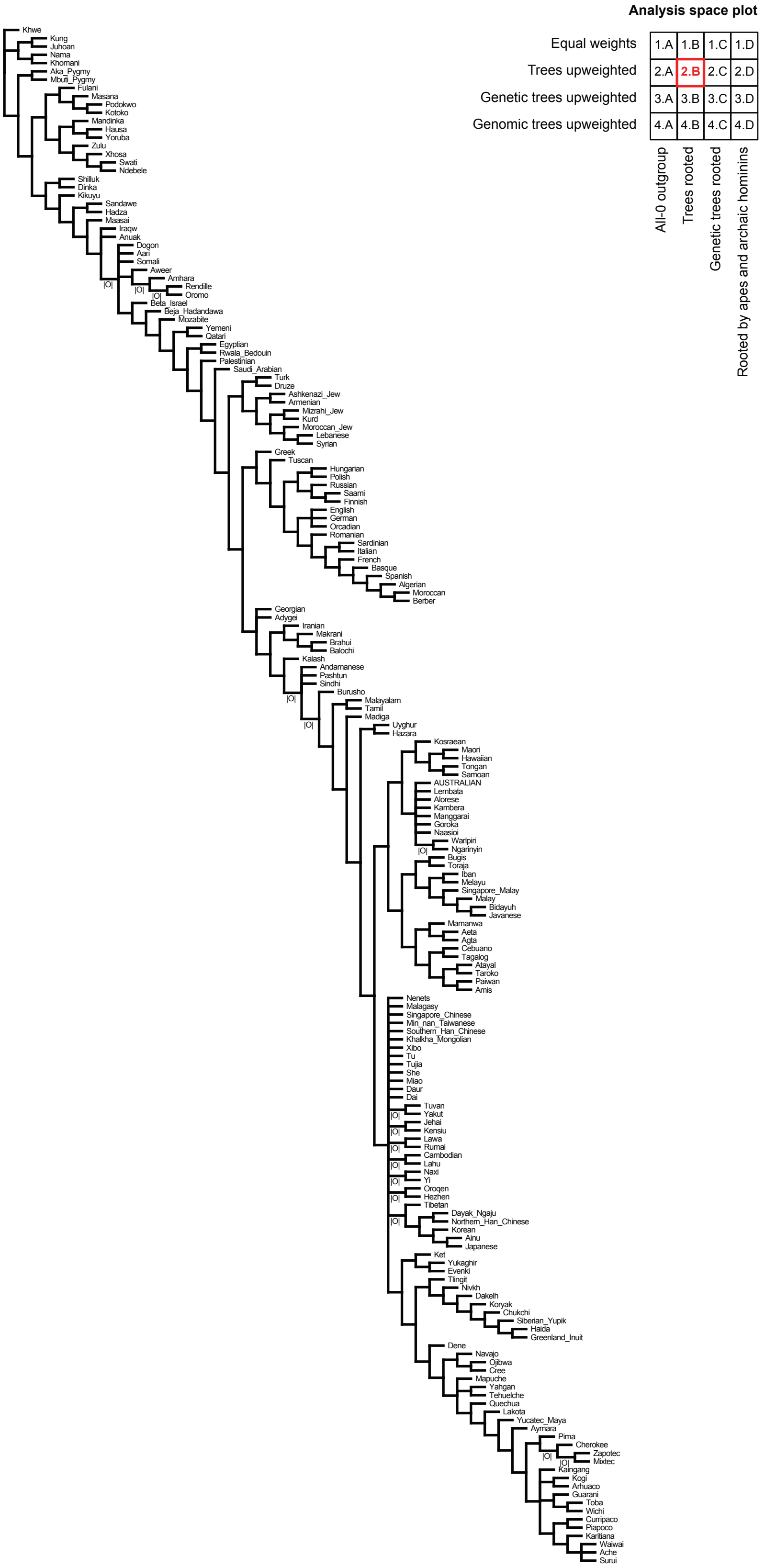

**Supplementary Figure S7.** Semistrict consensus supertree based on parameter set 2.C of the sensitivity analysis of the representative dataset. A symbol "|O|" indicates clades which would be collapsed into polytomies in a strict consensus supertree.

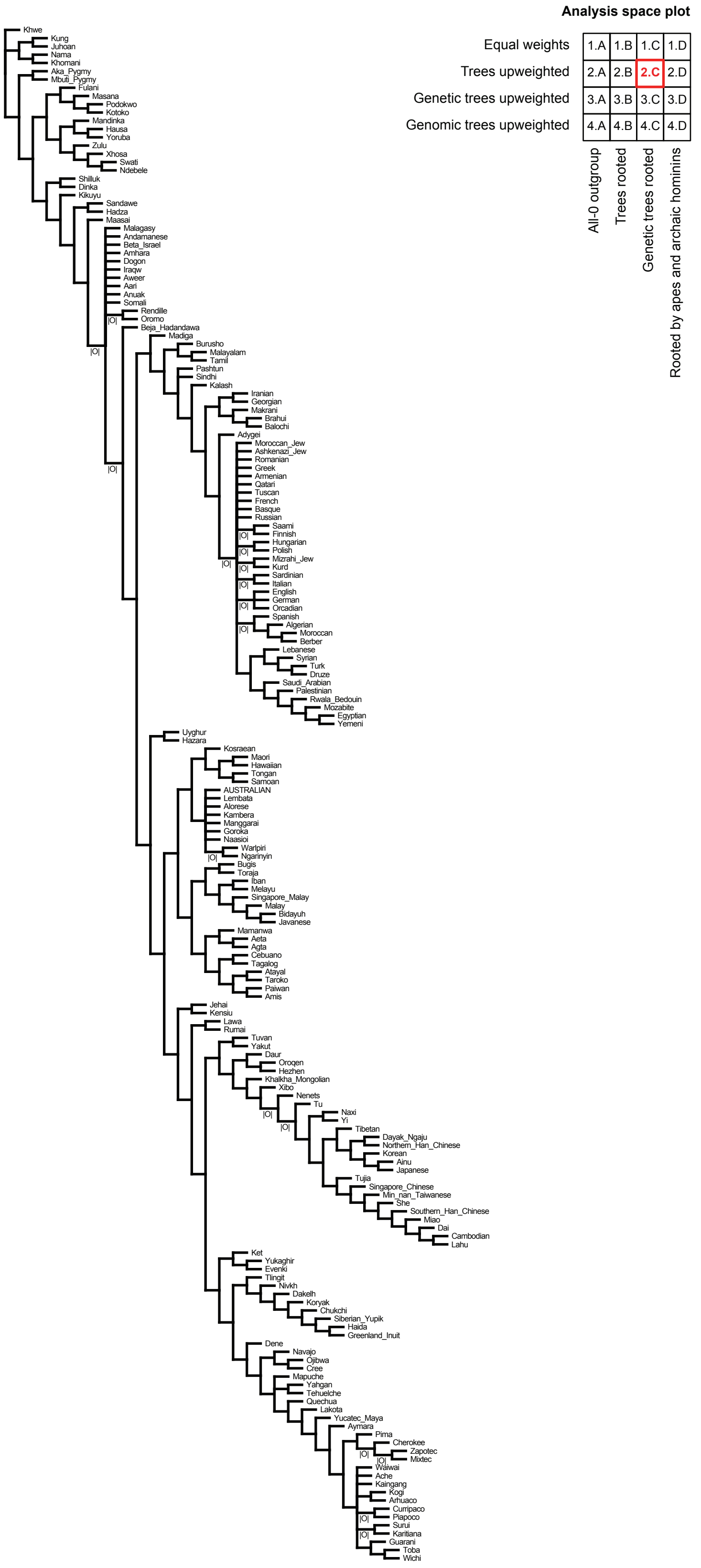

**Supplementary Figure S8.** Semistrict consensus supertree based on parameter set 2.D of the sensitivity analysis of the representative dataset. A symbol “|O|” indicates clades which would be collapsed into polytomies in a strict consensus supertree.

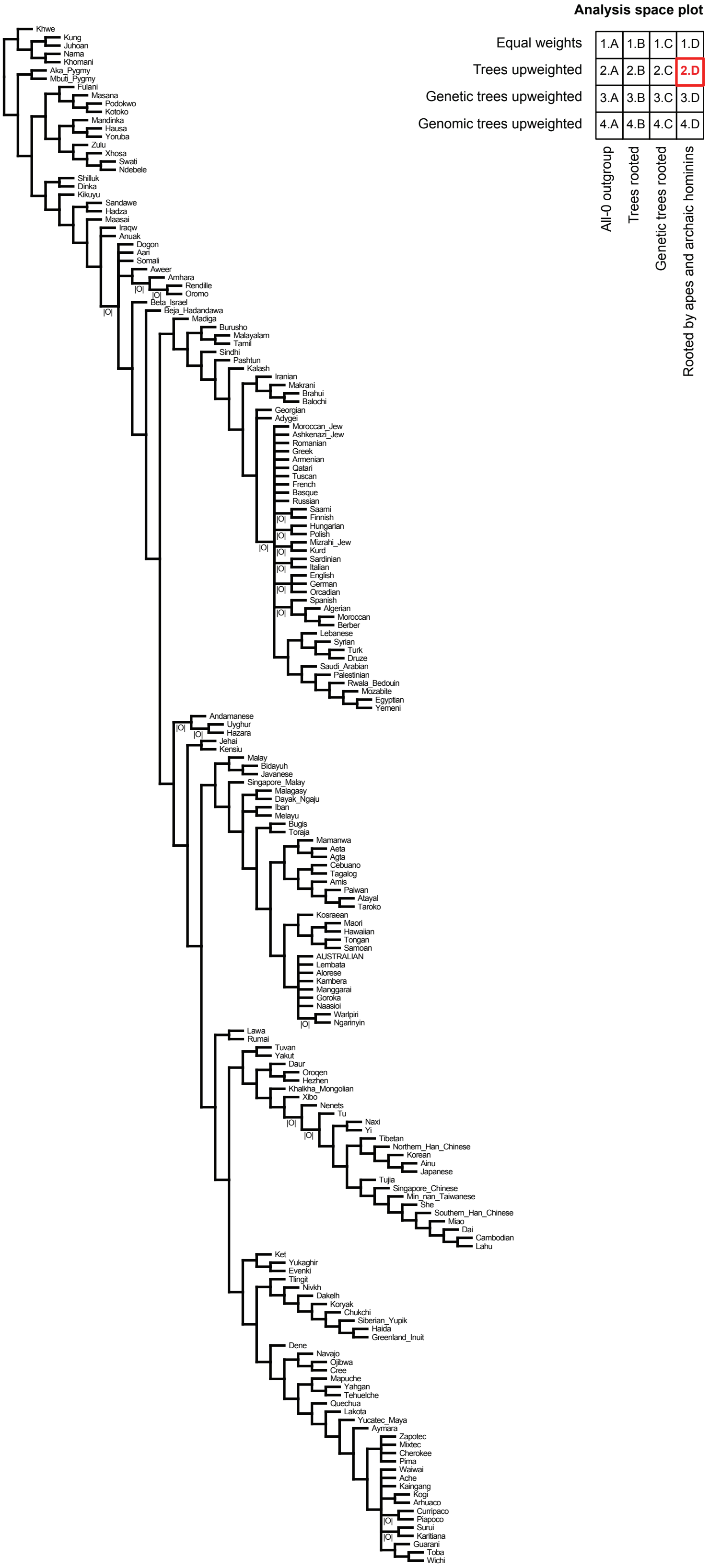

**Supplementary Figure S9.** Semistrict consensus supertree based on parameter set 3.A of the sensitivity analysis of the representative dataset. A symbol "|O|" indicates clades which would be collapsed into polytomies in a strict consensus supertree.

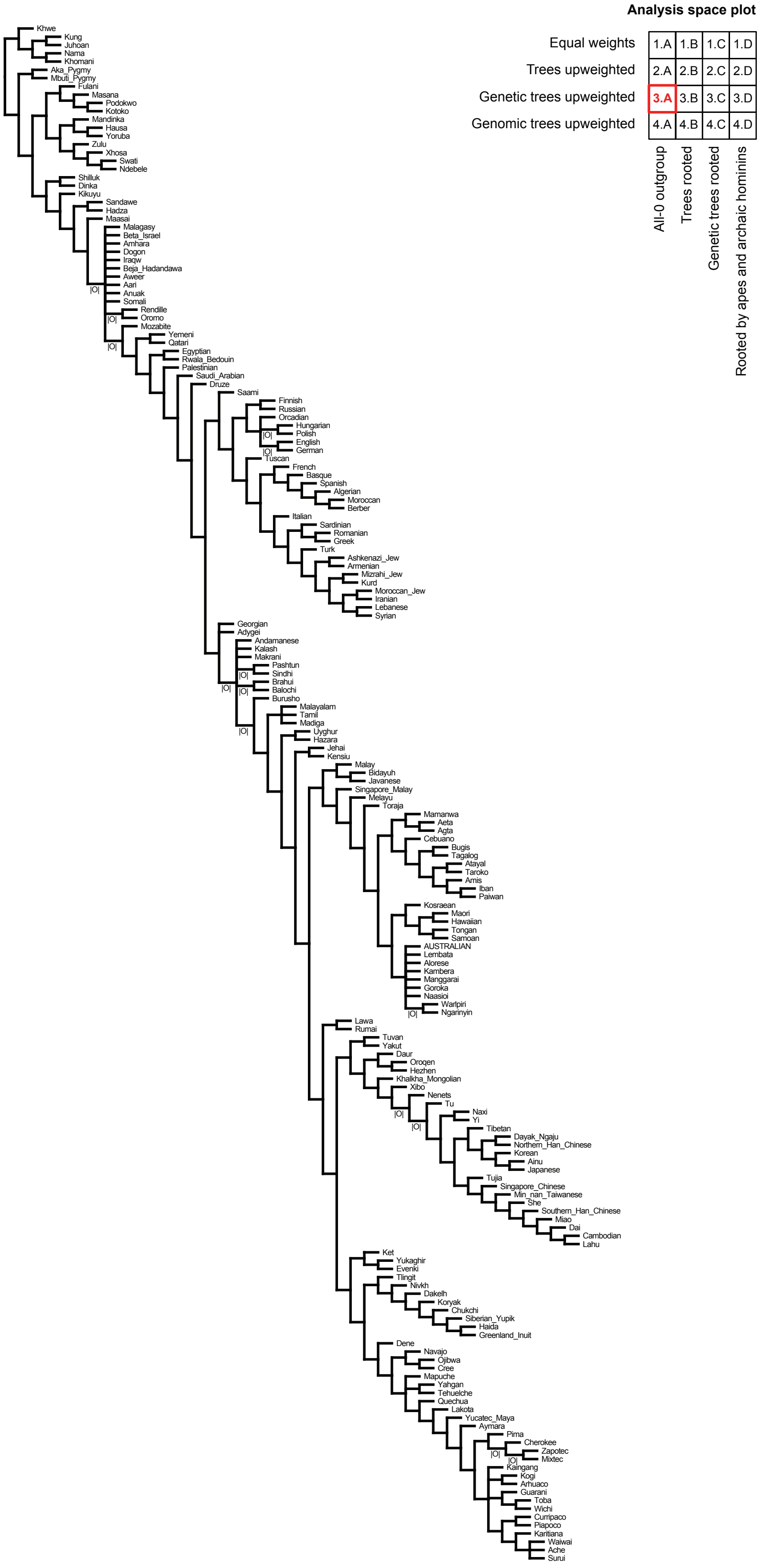

**Supplementary Figure S10.** Semistrict consensus supertree based on parameter set 3.B of the sensitivity analysis of the representative dataset. A symbol "|O|" indicates clades which would be collapsed into polytomies in a strict consensus supertree.

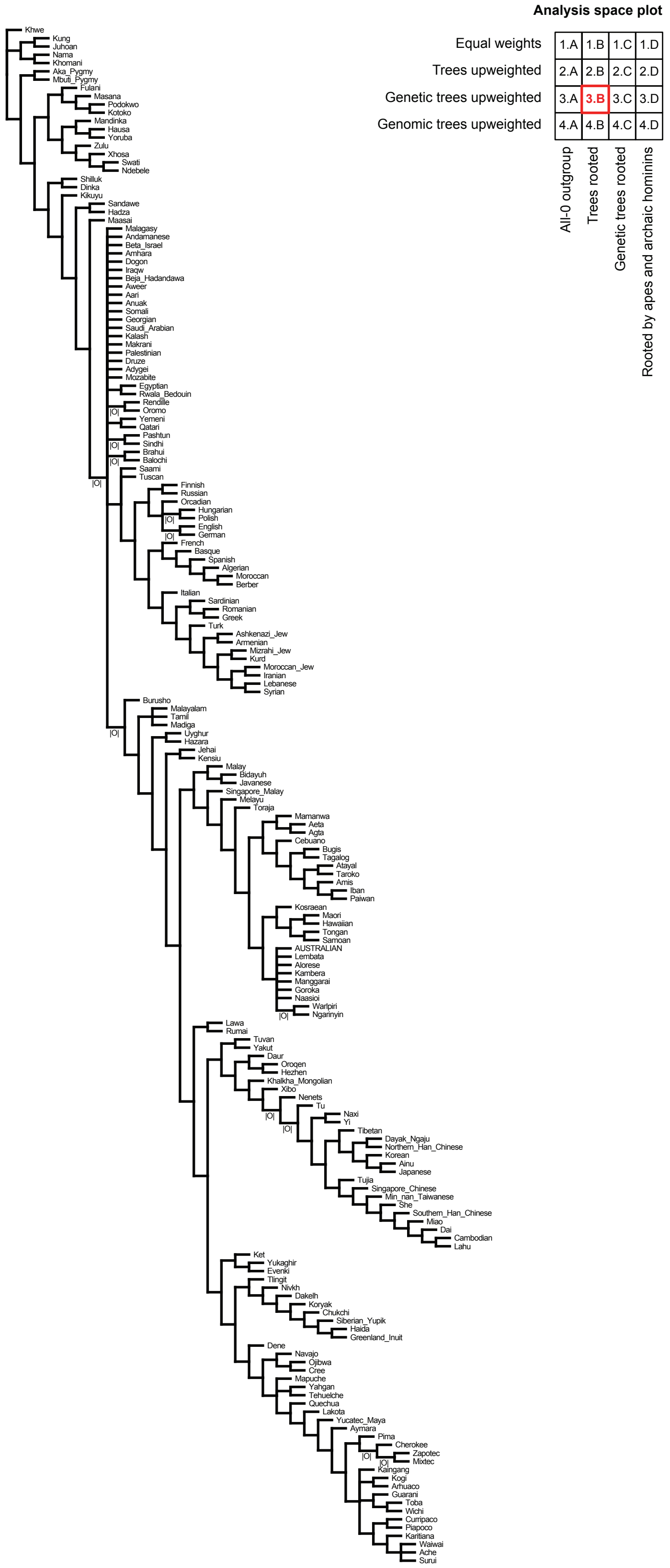

**Supplementary Figure S11.** Semistrict consensus supertree based on parameter set 3.C of the sensitivity analysis of the representative dataset. A symbol "|O|" indicates clades which would be collapsed into polytomies in a strict consensus supertree.

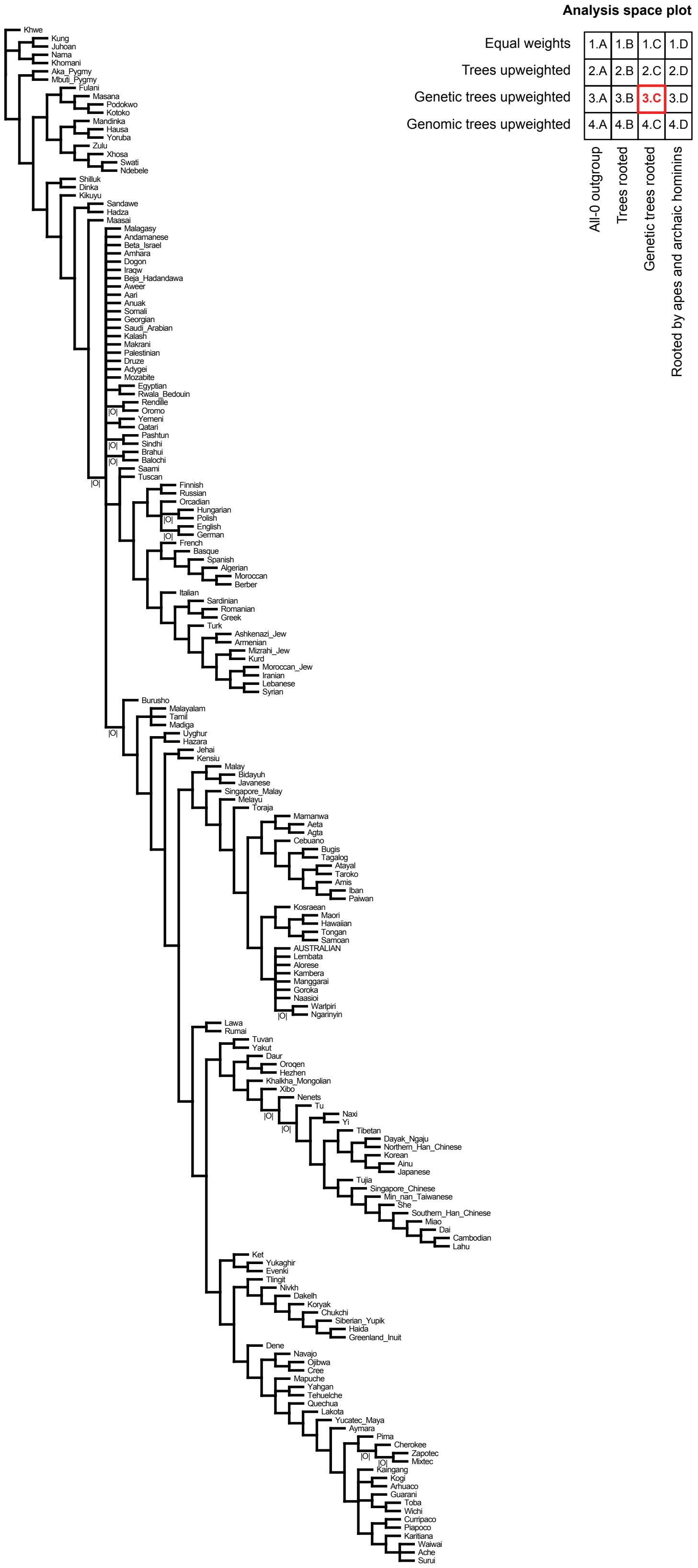

**Supplementary Figure S12.** Semistrict consensus supertree based on parameter set 3.D of the sensitivity analysis of the representative dataset. A symbol “|O|” indicates clades which would be collapsed into polytomies in a strict consensus supertree.

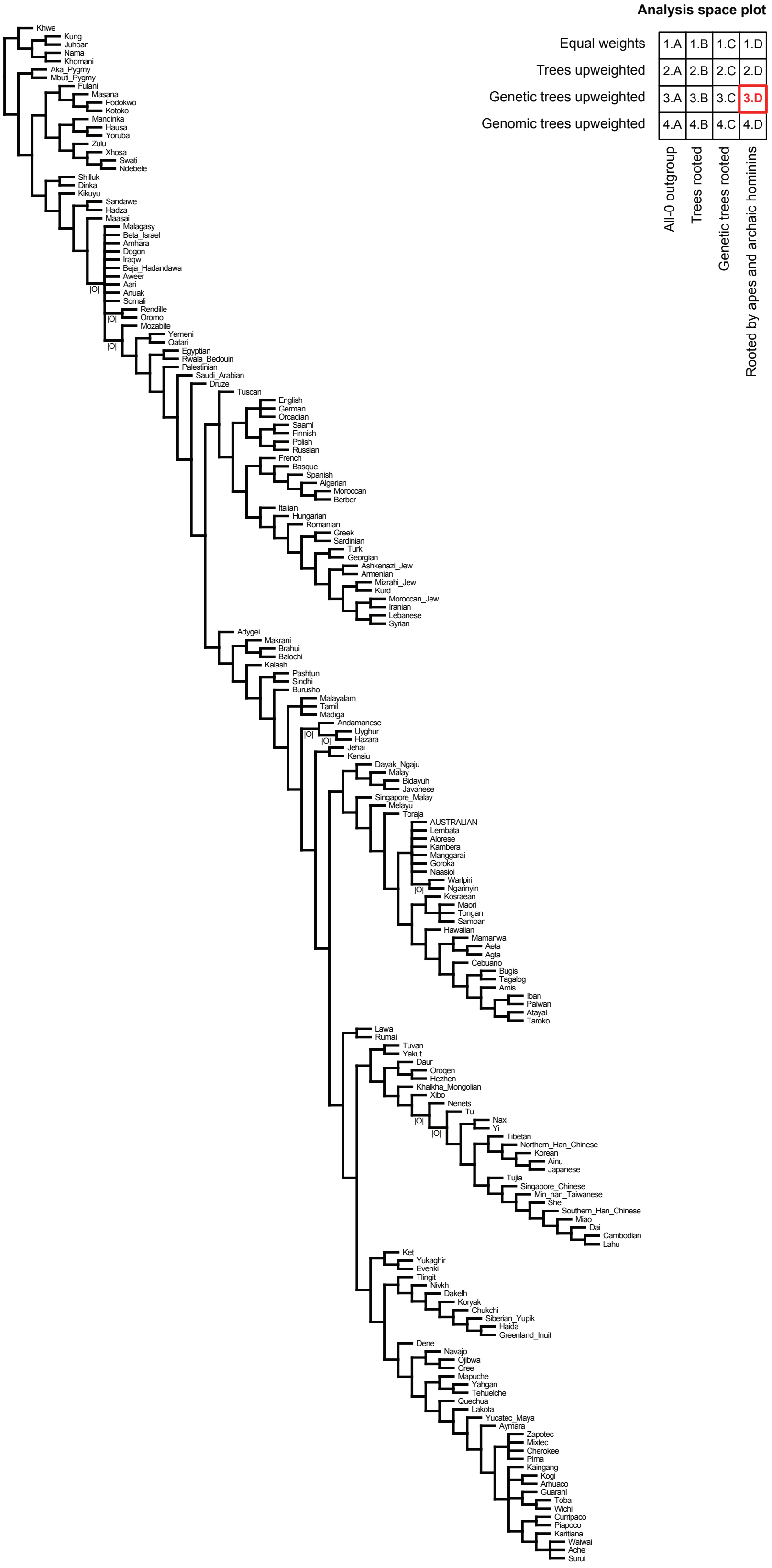

**Supplementary Figure S13.** Semistrict consensus supertree based on parameter set 4.A of the sensitivity analysis of the representative dataset. A symbol "|O|" indicates clades which would be collapsed into polytomies in a strict consensus supertree.

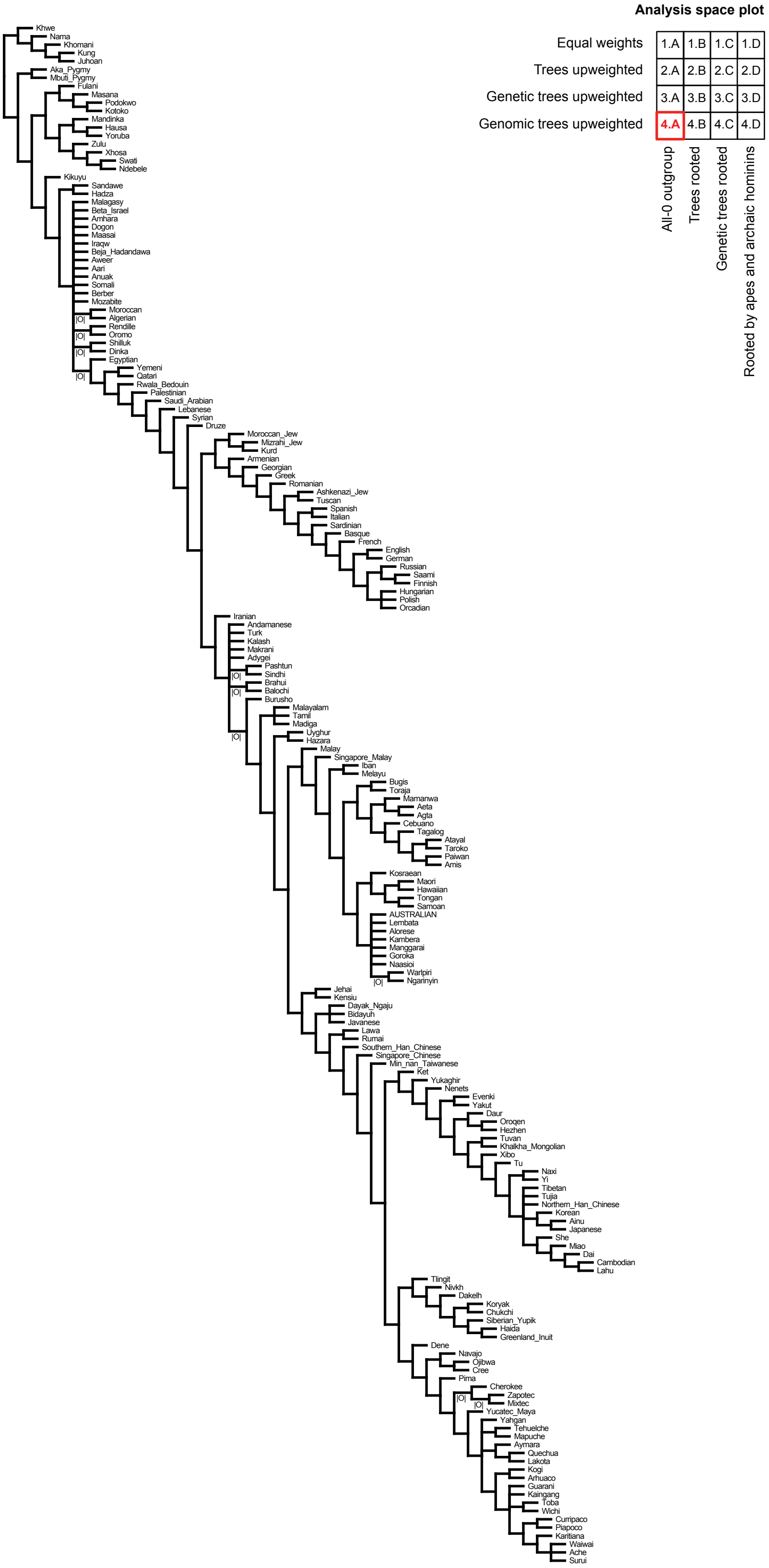

**Supplementary Figure S14.** Semistrict consensus supertree based on parameter set 4.B of the sensitivity analysis of the representative dataset. A symbol “|O|” indicates clades which would be collapsed into polytomies in a strict consensus supertree.

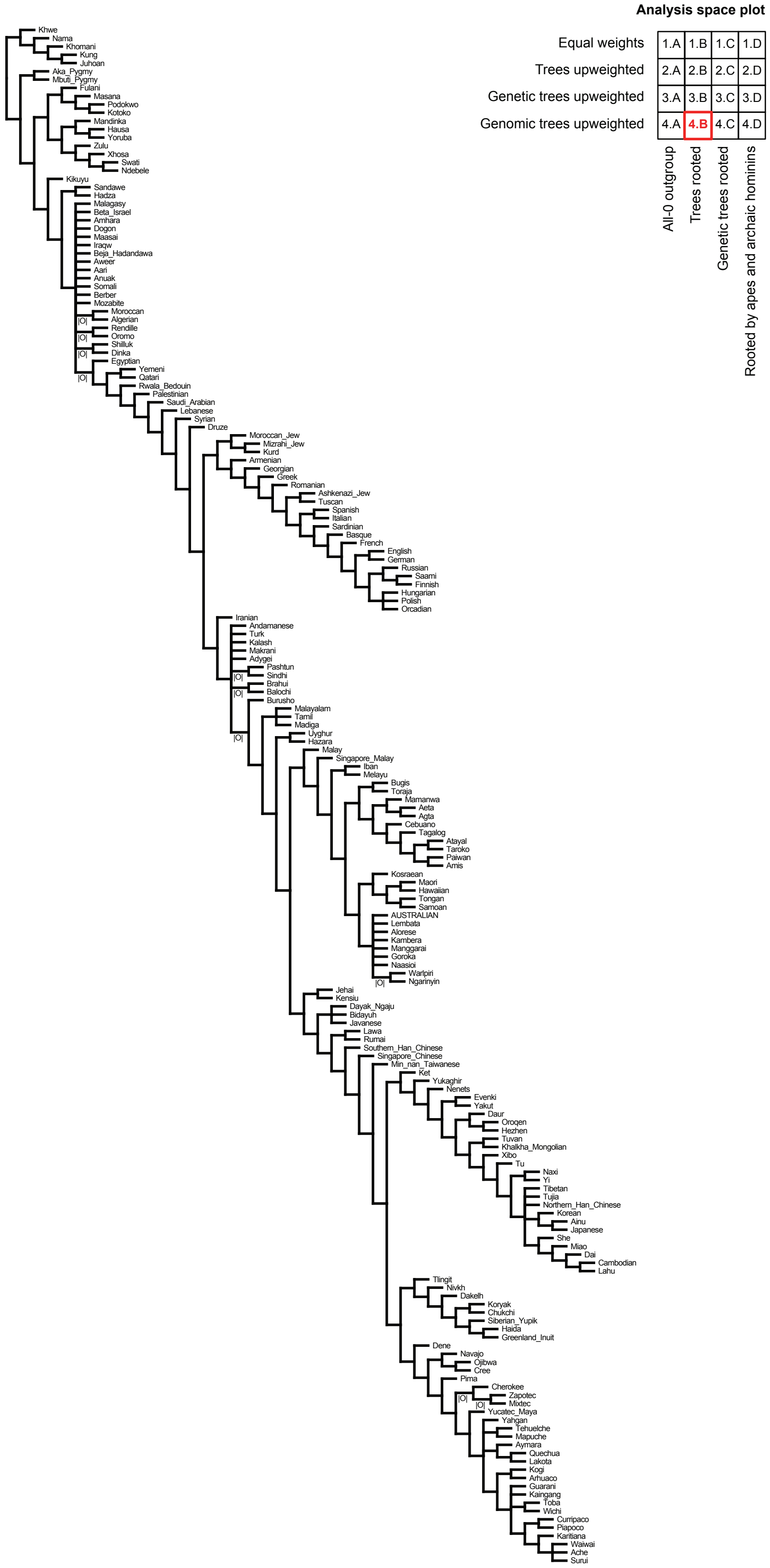

**Supplementary Figure S15.** Semistrict consensus supertree based on parameter set 4.C of the sensitivity analysis of the representative dataset. A symbol "|O|" indicates clades which would be collapsed into polytomies in a strict consensus supertree.

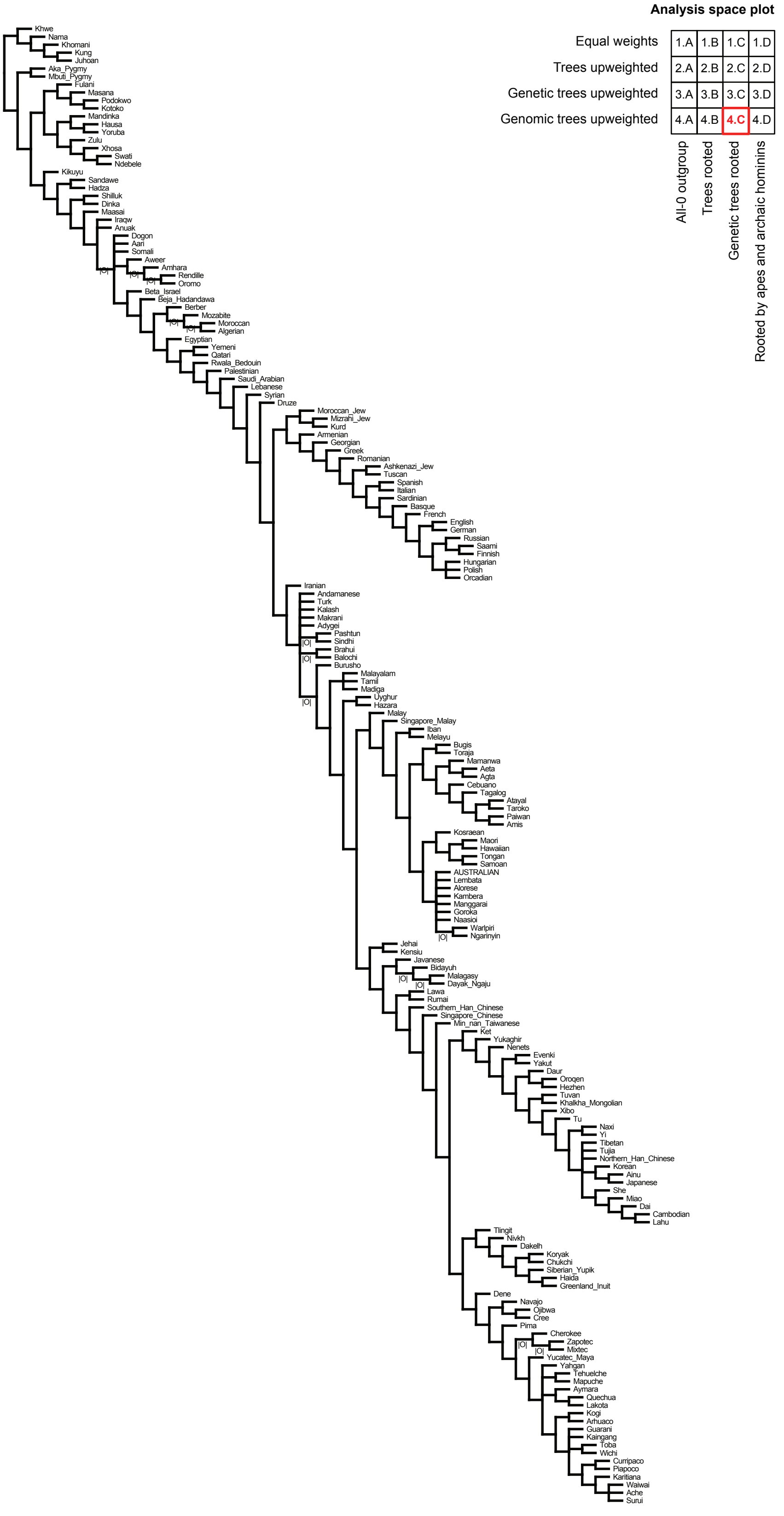

**Supplementary Figure S16.** Semistrict consensus supertree based on parameter set 4.D of the sensitivity analysis of the representative dataset. A symbol "|O|" indicates clades which would be collapsed into polytomies in a strict consensus supertree.

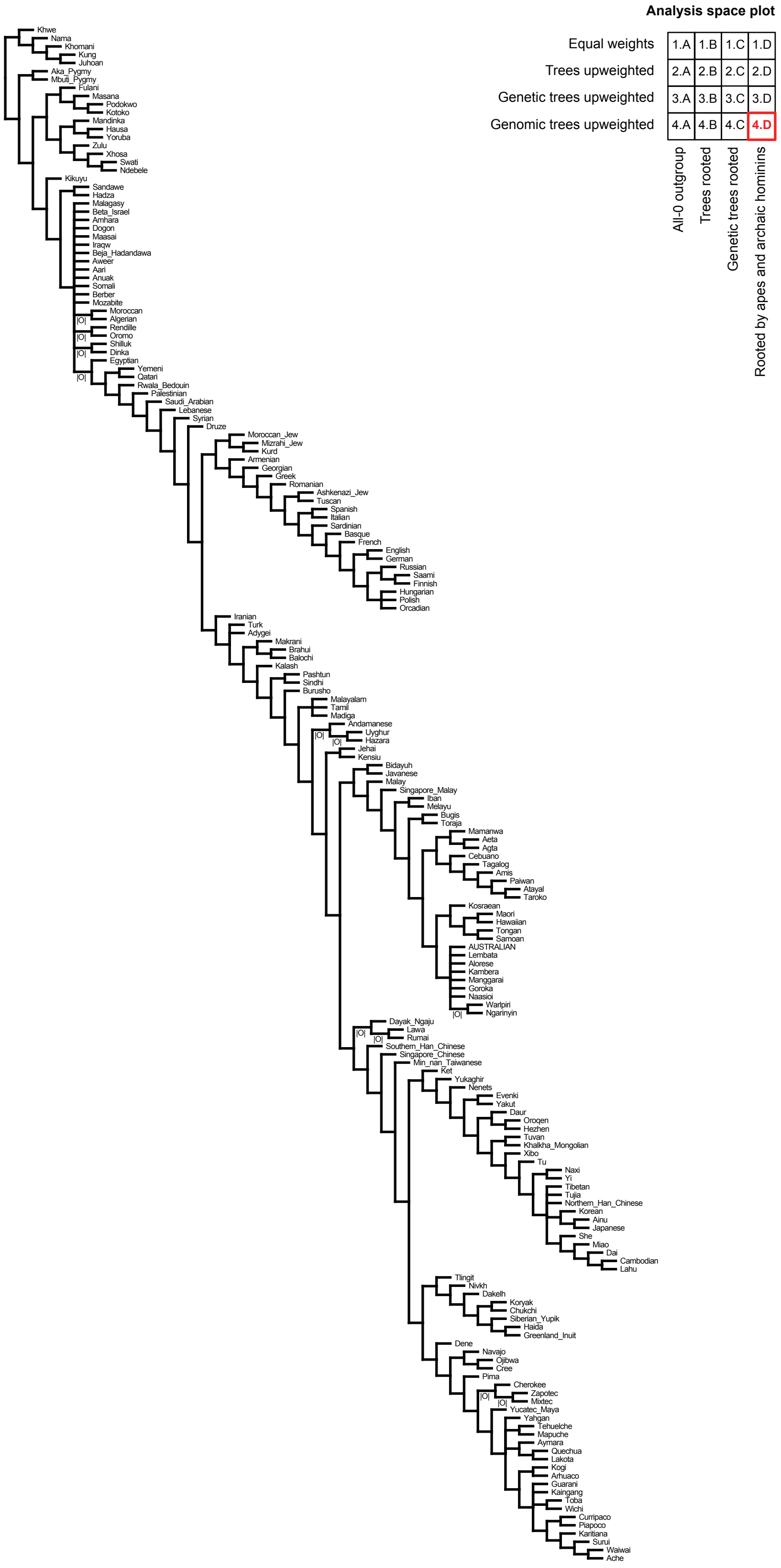

**Supplementary Figure S17.** Pruned strict consensus supertree based on parameter set 1.C of the representative dataset. Alternative positions of the identified wildcard taxa are indicated. A symbol “>” indicates wildcard taxa which would be placed within a polytomy, if included in the analysis.

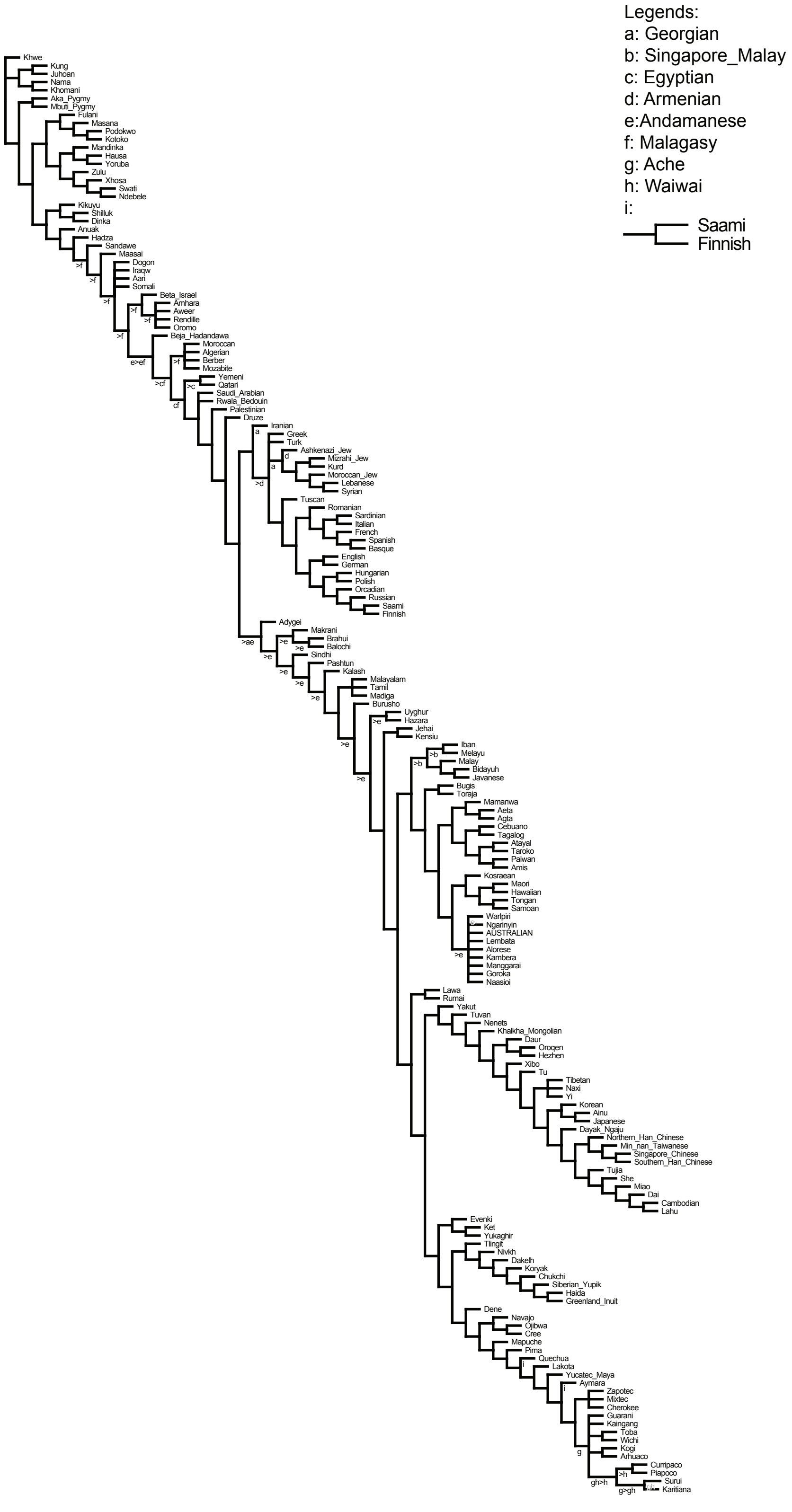

**Supplementary Figure S18.** Pruned strict consensus supertree based on parameter set 1.D of the sensitivity analysis of the representative dataset. Alternative positions of the identified wildcard taxa are indicated. A symbol “>” indicates wildcard taxa which would be placed within a polytomy, if included in the analysis.

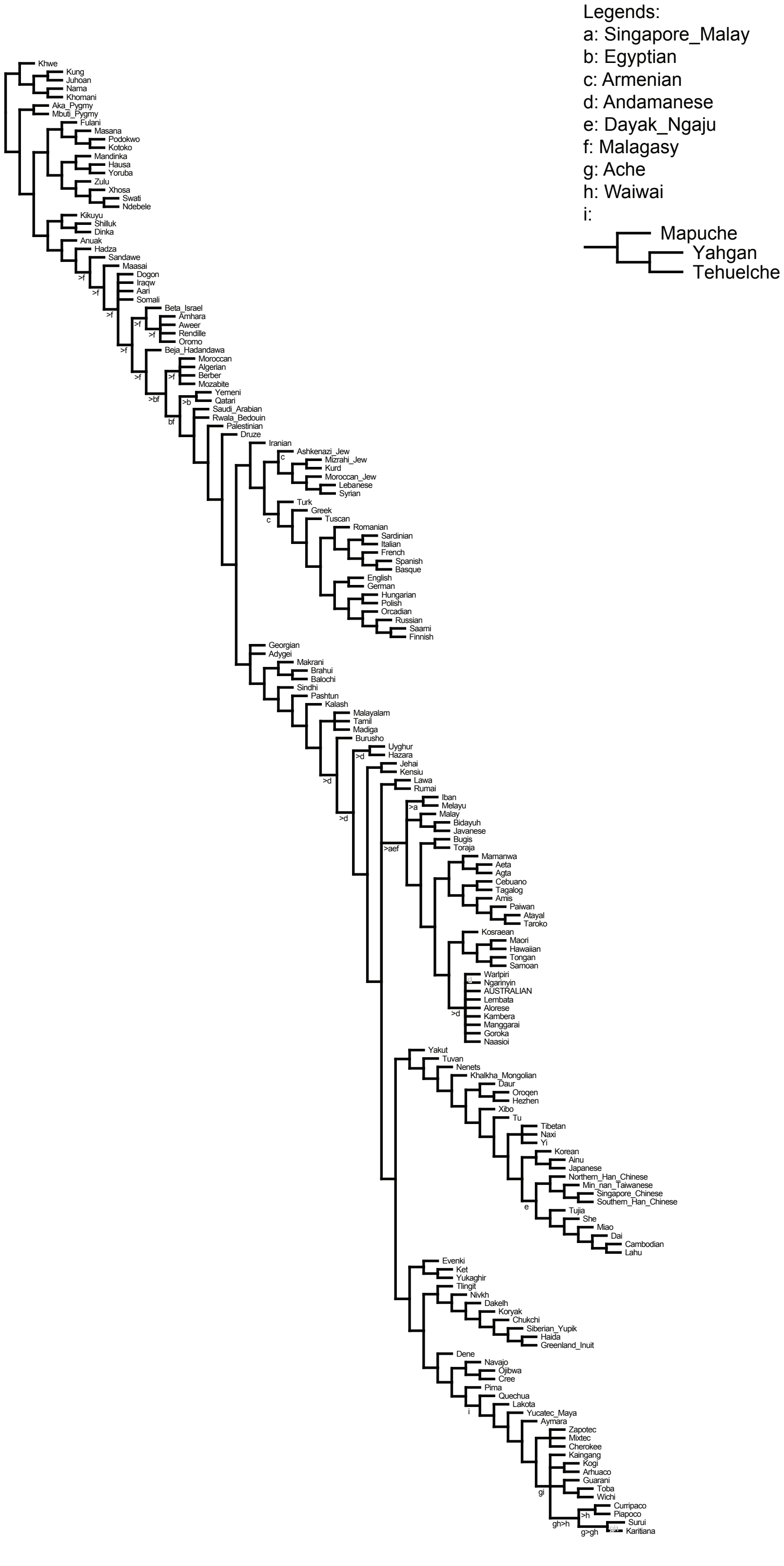

**Supplementary Figure S19.** Semistrict consensus supertree based on admixture plots.

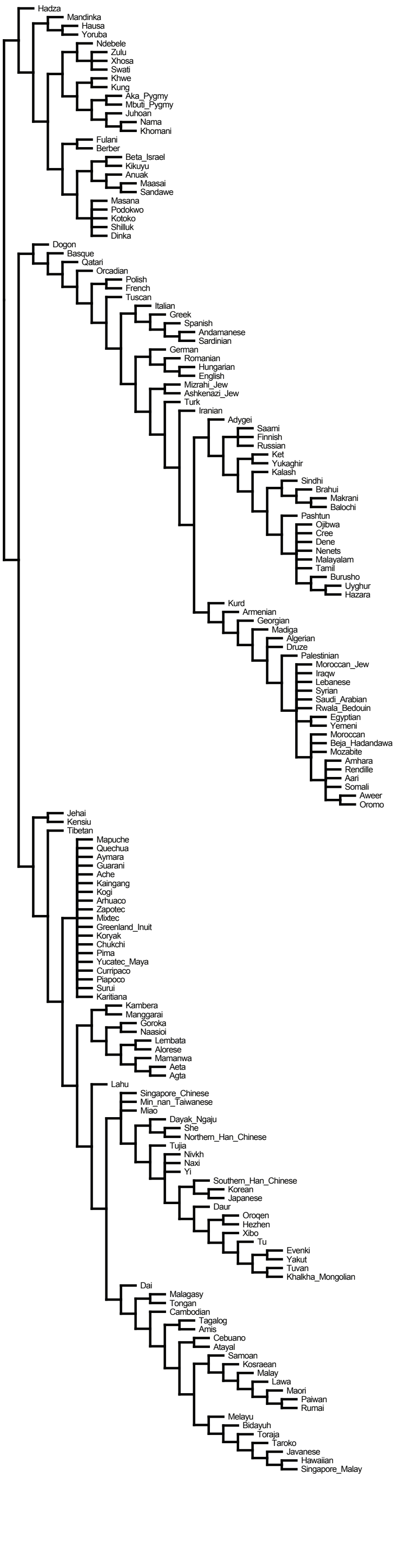

**Supplementary Figure S20.** Pruned strict consensus supertree based on admixture plots. Alternative positions of the identified wildcard taxa are indicated. A symbol “>” indicates wildcard taxa which would be placed within a polytomy, if included in the analysis.

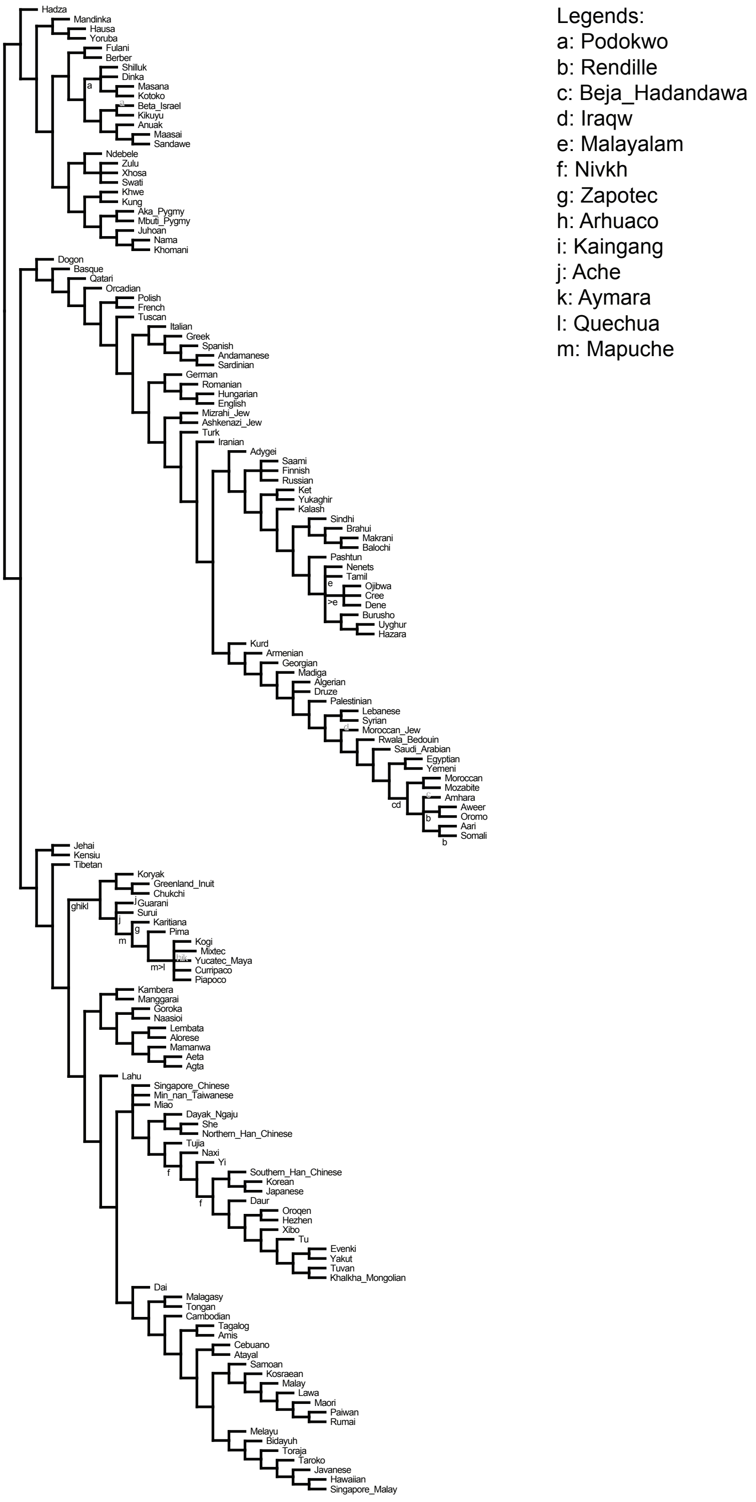

**Supplementary Figure S21.** Semistrict consensus supertree based on parameter set 1.A showing the taxa for which there are informative linguistic characters and the clades supported by linguistic characters (in red).

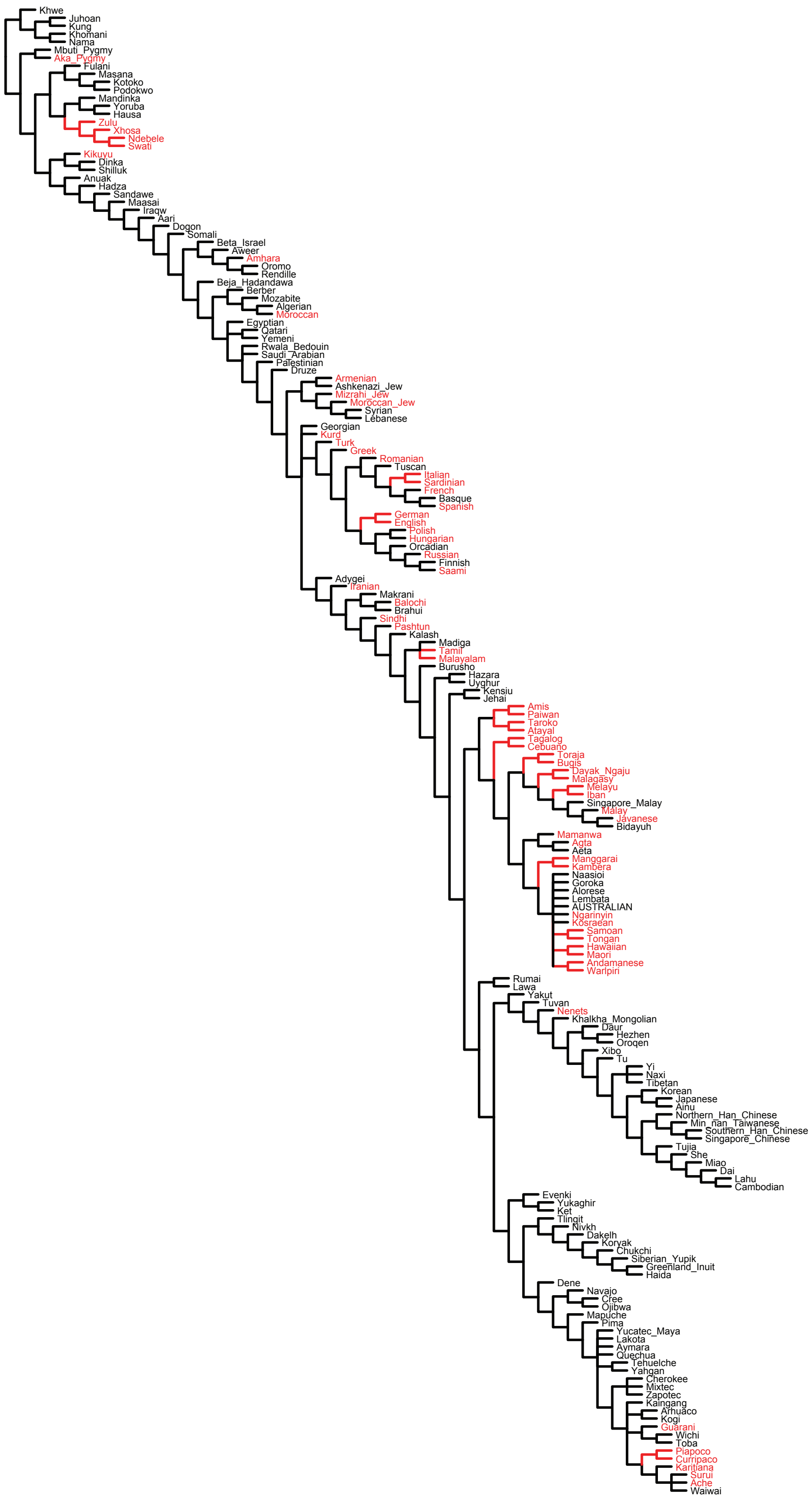

**Supplementary Figure S22.** (**a**) Supertree constrained by Greenberg-Ruhlen dataset based on linguistic classification from *Ethnologue*, Ruhlen, and Greenberg & Ruhlen. White circles indicate linguistic topological constraints. Grey circles indicate an unconstrained taxon or clade (usually a language isolate) recovered within a constrained one. (**b**) A section of the supertree constrained by linguistic classification of the Amerind macrofamily based on Greenberg & Ruhlen[2] but without a constraint on the monophyly of the Amerind itself.

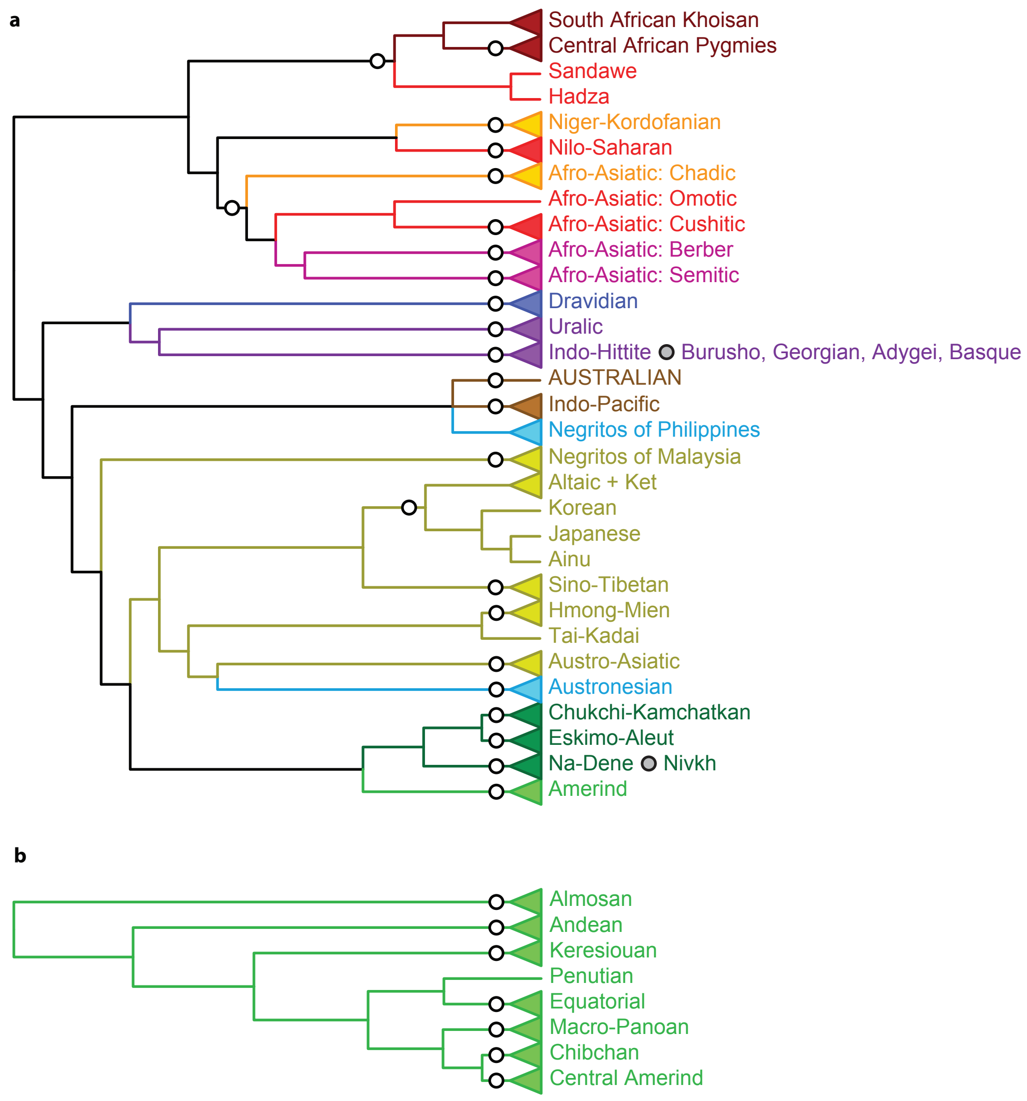

**Supplementary Figure S23.** Tanglegram comparing supertrees based on a representative dataset. (**a**) The purely genetic supertree. (**b**) Galled network based on two supertrees constrained by linguistic classification based on Ruhlen[1]. Branches supported by linguistic information (i.e., informative characters based either on the linguistic classification datasets or on the linguistic source trees) are in black. Branches based solely on genetic information are in gray. Instances of recent language shifts in selected populations (C African Pygmies, "Negritos" of Malaysia and Philippines, and Ashkenazi Jews) are indicated by reticulations in red. Dashed red lines indicate the phylogenetic placement of the population based on its linguistic affiliation, solid red lines indicate the phylogenetic placement of the population based on genetic/genomic data. Brackets delimit corresponding monophyletic or paraphyletic groups in the two supertrees. Black lines connect corresponding (monophyletic or paraphyletic) groups, gray lines connect terminal taxa. Both trees are right-ladderized.

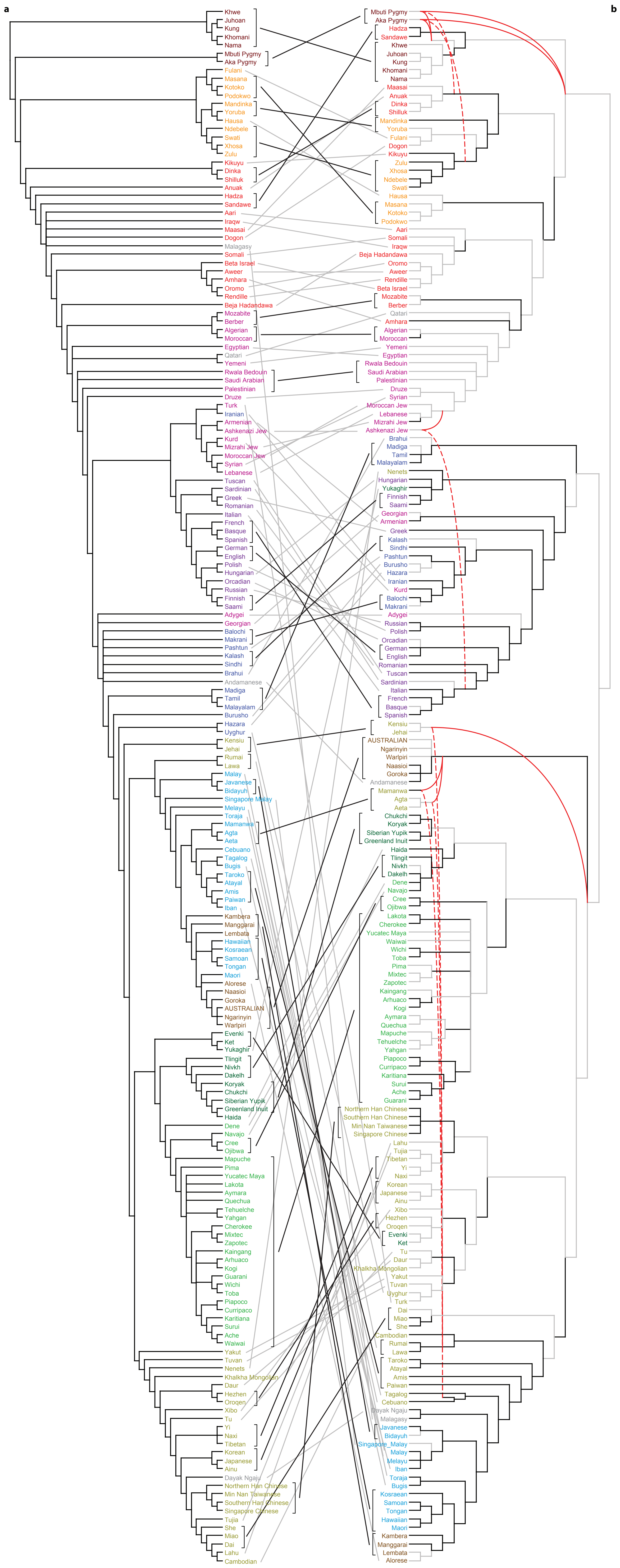

**Supplementary Figure S24.** (**a**) An “anticonsensus” tree showing groups present in the purely genetic supertree (Supplementary Figure S23a) that are absent from the supertree constrained by linguistic classification (Supplementary Figure S23b). (**b**) An “anticonsensus” tree showing groups in the supertree constrained by linguistic classification (Supplementary Figure S23b) that are absent from the genetic supertree (Supplementary Figure S23b). Polytomies indicate absence of conflict. A symbol “|O|” indicates compatible groups, a symbol “|X|” indicates contradictory groups.

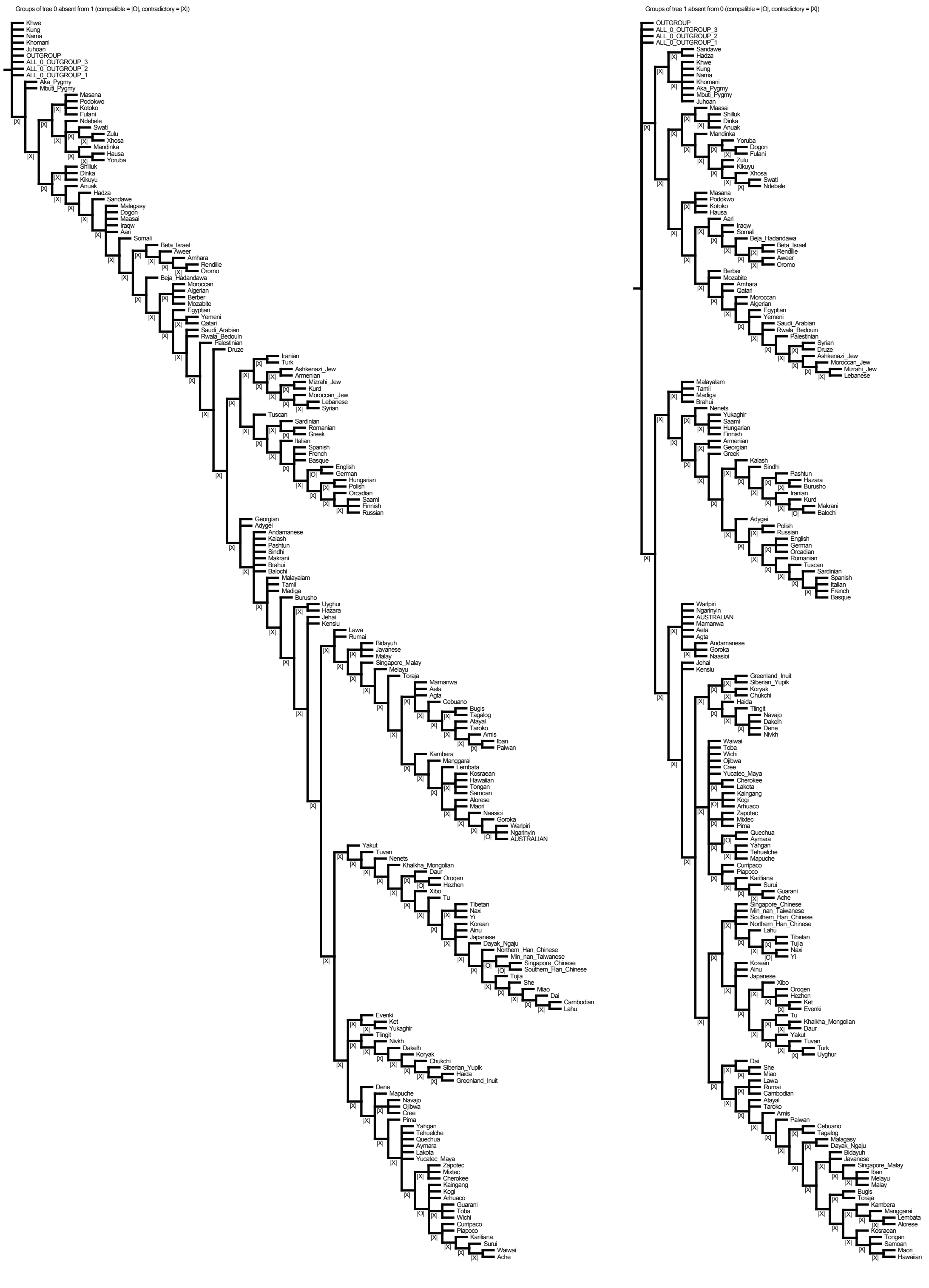

## SUPPLEMENTARY METHODS

### Data collection and manipulation

**Source trees.** The source trees were searched on the electronic databases Web of Science, ScienceDirect, and Google Scholar. Complete bibliographies of all collected papers, as well as papers that cite them, were searched in order to find additional source trees.

Only the source trees that were inferred by formal phylogenetic methods and based on original analyses of real datasets (both genetic and linguistic) were included. To ensure accessibility of data and accountability of the supertree *sensu* Gatesy et al.[3] with respect to its source data, only trees published in peer-review sources (including web journals) were collected. The only exception was an unpublished extension[2] of the published article[4] and two conference papers[5,6]. Trees published exclusively as parts of doctoral dissertations were not included. The tree-like schemes derived solely from the formal linguistic classifications were not considered valid source trees. Trees and admixture plots that include, exclusively or predominantly, mixed ancestry populations such as African Americans, Mexican Mestizos, or Cape Coloreds[7,8] were not included, nor were the trees including only subpopulations of a single population[9,10].

The source trees were translated exactly as they appear in print. Standardization of taxonomic nomenclature and taxonomic level, as well as pruning of invalid taxa, was performed after the translation of a tree into matrix representation (MR).

Both rooted and unrooted trees of human populations or languages were utilized. Trees including individuals or individual genotypes of known ethnic population origin instead of ethnic populations[11–12] were utilized also. Replacement of individuals/individual genotypes with respective populations sometimes resulted in the MR implying that a terminal taxon is placed at multiple positions of a tree. These taxa were then fused together, which resulted in a less resolved tree.

The translation routine applies generally to all phylogenetic trees regardless of the method used for estimating phylogeny (i.e., UPGMA, neighbor-joining, maximum parsimony, maximum-likelihood, and Bayesian inference methods). The trees constructed by the program TreeMix[4] were also utilized, with the “admixture edges” omitted during translations to MR.

**Admixture plots.** In addition to the source trees, we utilized admixture plots for the first time as additional sources of data for the supertree construction. Admixture plots are graphical outputs of programs STRUCTURE[13], FRAPPE[14], and ADMIXTURE[15], developed to estimate individual ancestry and population structure on the basis of recombining genetic markers such as single nucleotide polymorphisms (SNPs), short tandem repeats (STRs), or sequence haplotypes. These programs have been utilized extensively in human population genetic studies. The STRUCTURE program has also been utilized to investigate the structure and history of human populations based on linguistic markers[16]. All the programs use iterative maximum-likelihood or Bayesian clustering algorithms that attribute individual genotypes of known ethnic population origin to K clusters such that Hardy–Weinberg equilibrium is

maximized within the clusters. Individuals are given a membership coefficient for each cluster such that the estimated membership coefficient of each individual sums to 1 across K clusters.

The graphical outputs of these programs are plots indicating proportions of the individual genotypes attributable to K clusters by color. Although these programs do not model history explicitly, the inferred clusters can be interpreted post hoc as representing historical populations, and individuals or populations that are mixtures of different components as evidence of admixture between these populations[4]. Hierarchical information contained within the admixture plot was converted into matrix representation as follows. Each population was coded as "present" ("1") or absent ("0") based on proportions of individual genotypes attributable to each cluster. Limited attribution to a given cluster (less than ca. 10%) was neglected, and ambiguous sections of a plot (borderline proportions or different proportions in individuals within a single population) were scored as "unknown" ("?"). The resulting matrix of additive binary characters was analyzed by maximum parsimony to produce a phylogenetic tree corresponding roughly to population clustering implied by the admixture plot. The trees based on admixture plot often contained unresolved sections due to membership of some populations in several clusters, but they still preserve enough valuable branching information.

**Controlling for data non-independence and duplication.** It is common practice in human population history studies that both molecular and linguistic datasets are obtained from the literature or open databases and re-used in a novel analysis. The re-use of the old data is facilitated by on-line archiving molecular datasets including the Human Genome Diversity Project (HGDP) panel[17] or the International HapMap Project[18], and linguistic datasets available online, such as the Austronesian Basic Vocabulary Database (ABVD)[19].

Many molecular phylogenetic analyses of human populations are therefore extensions of previous studies. This applies especially to analyses based on genome-wide data and on human leukocyte antigen system (HLA). Also, analyses of the same linguistic groups are often based on identical sets of cognates. As a result, the same character information can contribute to more than one source tree. In all cases of data duplication, the overlap of character data between source studies means that the associated source trees are not independent of one another, a key assumption of phylogenetic analysis. Non-independence can arise among trees from different studies, as well as among trees presented within a single source study ("between-" and "within-study non-independence", respectively[20]). The consequence of data non-independence and the associated data duplication is that some data partitions are effectively upweighted and might affect the supertree topology disproportionally[3,21]. While the problem of data set non-independence cannot be eliminated entirely, it can be largely ameliorated using an appropriate source tree collection and exclusion protocol.

The most widely used protocol called "garbage in, garbage out"[20] is difficult to implement since there is no explicit dependence threshold between analyses, and its strict application neglects a huge amount of data and likely oversimplifies the existing phylogenetic controversies[22]. It is also likely to introduce bias in the supertree dataset due to exclusion and downweighting of matrices based on source trees derived from overlapping datasets. For these

reasons, we used the so-called "less restricted" protocol for source tree retention and exclusion[22]. In this approach, studies derived from modifications of previous datasets (e.g., new scoring for certain taxa, addition of taxa or characters) were regarded as separate analyses. If a paper provides two or more different topologies, derived from modifications of the same dataset (e.g. addition/exclusion of few taxa, different scoring for some characters), these were included as separate source trees. However, secondary representations of a tree taken from another study were not considered valid source trees.

**Standardization of taxonomic nomenclature and taxonomic level.** Merging MRs of the source trees by terminal taxa requires prior standardization of taxonomic nomenclature. Standardizing taxonomic nomenclature throughout the source trees is a daunting task since there is no universally accepted taxonomic nomenclature of human populations. Numerous alternate names of human ethno-linguistic groups (ethnonyms) exist[23], reflecting differences between population and language names, between auto/endonyms and exonyms, political influences, or merely different spelling.

Taxonomic nomenclature was standardized using ISO 639-3 codes from *Ethnologue*[23], a reference work cataloging all of the world's known living and recently extinct languages. Information on geographic range of a population in question, sampling location(s) of genotyped individual(s), language affiliation and ethnonyms was utilized in order to standardize taxonomy of the terminal taxa within and between source trees[23-29]. Data provided by Dediu & Ladd[30] were used to match groups of individuals included in HGDP panel[17] with the most likely corresponding ethno-linguistic groups.

Auto/endonyms were not systematically preferred over exonyms, since most of them are scarcely used and largely unknown to both the general public and expert audience. We avoided the use of exonyms that are nowadays considered derogatory (e.g., "Bushmen", "Hottentot", "Semang", "Eskimo", "Chipewyan", "Mataco Indians"[23]).

The source trees often include taxa (or operational taxonomic units, OTUs) that are hierarchically nested one in another as different studies investigate human population structure and history on different levels. The taxa for molecular analyses are often less specified in comparison with taxa used in linguistic analyses. Nested taxa appear in published human population-level phylogenetic trees as well as in admixture plots. In some cases, taxa that are hierarchically inter-dependent appear within a single source tree (e.g., "S. Bantu" and "Xhosa" in Tishkoff et al.[31]).

The taxonomic level of the terminal taxa was standardized to correspond to ethno-linguistic groups listed in *Ethnologue*[23]. Lower-level taxa (i.e., subpopulations) took on the names of the corresponding higher-level taxa in *Ethnologue*. Higher-level taxa (i.e., above the level of groups listed in *Ethnologue*) were replaced by constituent lower-level taxa based on the information provided in the source study. When this information was unavailable, "type" population(s) were used in place of the higher taxon. They were selected with respect to the sampling location(s), language affiliation of the genotyped individual(s), and other available information (Supplementary Table S1).

Multiple taxa from a single source tree representing subpopulations were fused together to form a single taxon in the supertree dataset. Some subpopulations that are clearly distinguishable geographically and/or culturally and are sufficiently represented across the source trees (e.g., Cypriot Greek, Singapore Chinese) were retained in the supertree dataset. When two or more subpopulations were present in the source tree in too distant positions so that their fusion would lead to a great loss of the resolution of the tree, only the most representative subpopulation (with respect to the number of genotyped individuals and the sampling location) was retained in the supertree dataset.

**Pruning invalid taxa.** Matrix representations of the source trees with standardized taxonomic nomenclature and taxonomic level were merged into a single supertree dataset and further edited in Winclada ver. 1.00.08[32].

The dataset was trimmed to include only valid taxa. Decisions on what constitutes a valid taxon were guided by the appearance of the taxa in source trees, representation of these taxa across the source trees, and the ability to unambiguously associate these taxa with ethno-linguistic groups listed in *Ethnologue*[23].

Mixed ancestry populations (e.g., "African American", "US Hispanic", or "Cape Mixed Ancestry") and colonial populations (e.g., Boer) were not considered valid taxa, but heavily admixed indigenous populations (e.g., Malagasy, Naasioi, Aleut, Yucatec Maya) were included. Extinct populations based solely on linguistic source trees were not considered, but the populations from genetic source trees that have recently lost their original languages (e.g., Zenu) were included. Creole languages (e.g., Haitian) and colonial forms of Indo-European languages (e.g., Afrikaans), based solely on linguistic source trees, were not considered valid, whereas indigenous populations speaking creole languages (e.g., Karretjie, Rabaul), present in genetic source trees, were retained. Loosely specified higher-level taxa in the source trees (e.g., "African", "S.W. Asian", or "Native North American"), for which constituent population(s) could not be identified nor type population(s) established, were excluded from the supertree dataset. The only exceptions were "Australian Aboriginals" and "Northern Australian Aboriginals" of unspecified ethnic population origin. Australia is poorly sampled and unspecified samples outnumber those that are specified in the source trees. All unspecified Australian and Northern Australian "Aboriginals" were fused together and analyzed as a single terminal taxon named "AUSTRALIAN", together with the ethnically specified Australian Aboriginal populations. Taxa present in just one linguistic tree or in a single admixture plot were not included.

**Population samples.** The resultant supertree dataset (unpublished) included 973 valid taxa (human populations) and 5 great apes or archaic hominins that featured in the source trees (*Gorilla gorilla, Pan paniscus, Pan troglodytes,* Denisova hominin, *Homo neanderthalensis*). The representation of 973 taxa in the dataset is highly unequal: Cheyenne were scored only for five (0.05%) and Northern Han Chinese for 4,153 (44.28%) "characters" ("matrix elements"[33]) out of 9,380 phylogenetically informative characters.

In order to facilitate comprehension and visualization of the inferred human population relationship patterns, and to minimize the influence of unequal representation of individual taxa in the combined data matrix, we created two datasets based on restricted samples of the supertree dataset. The first dataset includes 186 populations and 5,717–5,987 phylogenetically informative characters, depending on outgroup selection ("representative dataset" hereinafter). This dataset included 52 populations from the HGDP panel[17,34]; 16 populations which were added to represent additional "ancestral components" identified on the basis of meta-analysis of 12 human genomic diversity projects by Shriner et al.[35]; and 16 more populations added to represent "ancestral components" that were admittedly missing in this meta-analysis[35] (i.e., Hadza, Sandawe, Fulani, Chadic, Cushitic, and Polynesian). Additional 102 populations were included in order to describe human diversity across world regions that are underrepresented in meta-analyses of human genomic diversity projects[35,36] (Island SE Asia, Melanesia, Australia, "Remote Oceania", Europe, Siberia, and North America). We included populations that seem to play a key role in understanding population expansions, as they could potentially represent sister or basal groups of the expanding population groups, e.g., populations of Caucasus (Armenian, Georgian, Adygei), Aboriginal Taiwanese (Amis, Paiwan, Taroko, Atayal), or E Siberians (Yukaghir, Ket, Nivkh). We also included language isolates and groups of debatable origin, e.g., Khwe, Andamanese, "Negritos" of Malaysia (Jehai and Kensiu) and Philippines (Agta, Aeta, and Mamanwa), Malagasy, Australian Aboriginals, Ainu, and Haida. The resulting dataset included both populations that are well represented across source trees, and based on overlapping genetic, genomic and linguistic trees and those that are poorly represented across source trees. Twenty-one populations in the dataset were not included in any source trees based on genome-wide data. These are mostly populations from Island South East Asia, Australia, and North America (Supplementary table S2). The least represented populations were Cherokee and Haida, both scored for 28 characters (0.47%), whereas the best represented Northern Han Chinese were scored for 3,272 characters (54.65%) out of 5,987 phylogenetically informative characters.

The second dataset was created in order to investigate the stability of principal phylogenetic groupings and to identify conflicts within the data, using only those populations that are best represented across the source trees. Its population sample consists of populations included in the HGDP panel[17,34] plus three additional populations: "AUSTRALIAN", Kosraean, and Samoan, representing Australia, Micronesia, and Polynesia, respectively ("HGDP dataset" hereinafter). This dataset included 52 populations and 2,874–3,070 phylogenetically informative characters, depending on outgroup selection. The least represented Kosraean were scored for 84 characters (5.99%), whereas the best represented Northern Han Chinese were scored for 2,250 characters (73.28%) out of 3,070 phylogenetically informative characters. Most populations in the dataset were scored for over 10% of phylogenetically informative characters.

## Analyses

**Phylogenetic analyses.** Phylogenetic analyses were performed in TNT ver. 1.1[37]. The following tree-searching algorithm was used for each analysis:

```
xinact; hold 10000; xmult= level 10 fuse 5 drift 30 rss css xss rat 50;
bbreak= tbr safe; tsave trees.ctf; save; tsave /; comcomp*; tchoose /;
tsave supertree.ctf; save; tsave /; tchoose -.;
```

The dataset was analyzed under "new technology search" with search level 10 using sectorial, ratchet, and tree fusing searches, obtaining trees from a 10,000-replicate random addition sequence, treating gaps as missing data and all character changes as equal and nonadditive. The recovered most parsimonious trees (MPTs) were then subject to additional branch swapping using a tree-bisection and reconnection method. The datasets were analyzed without any topological constraints (i.e., without any assumptions on monophyly of any geographical region or linguistic group). Up to 10,000 MPTs and one semistrict consensus supertree was recovered in each of 16 parameter sets of the sensitivity analysis (see below). Tree length, consistency index (CI) and retention index (RI) for the resulting supertrees were calculated after removal of parsimony-uninformative characters.

**Sensitivity analysis.** One of the major drawbacks of the phylogenetic supertree method is the inapplicability of standard resampling or step-counting techniques for evaluating tree support (i.e., bootstrap and jackkife support, or Bremer support) and the general inability to measure uncertainty of inferred phylogenetic groupings (but see Bininda-Emonds[38] and Wilkinson et al.[39]).

To investigate stability of the inferred supertree topology, and to identify conflicts within the data (i.e., to identify alternative taxonomic groupings inherent to the data), we used a method inspired by sensitivity analysis *sensu* Wheeler[40]. Sensitivity analysis is carried out by selecting a set of parameters and examining the effect of these parameter sets on the stability of inferred tree topologies. The sensitivity analysis examines the influence of parameter sets on taxonomic groupings recovered in the analysis. The results are graphically represented using so called "analysis space plots"[40] or "sensitivity plots"[41]. In this way, a sensitivity analysis can discern between robust clades (those that appear under most or all parameter set combinations) and unstable ones (those that appear only under one or few parameter sets).

The supertree dataset was analyzed under 16 different sets of values for the analysis parameters. These 16 analyses consisted of combinations of four weighting schemes and four rooting options. Successive downweighting of data partitions and rooting by different outgroups leads to weakening the influence of respective data partitions on overall topology of the supertree. This allows to assess the support for various phylogenetic groupings across various types of data and to identify the causes of topological conflicts. The weighting schemes and rooting options were as follows:

Either (1) all data partitions were weighted equally, or (2) genetic and linguistic trees (5,066 phylogenetically informative characters) were upweighted by a factor of 1,000 relative to admixture plots; or (3) genetic trees (4,428 phylogenetically informative characters) were upweighted relative to linguistic trees and admixture plots; or (4) genomic trees data (1,704 phylogenetically informative characters) were upweighted relative to all the remaining data partitions; and either (A) all rooted source trees and admixture plots were treated as rooted (by inserting a hypothetical „all-0“ outgroup), or (B) only the trees were treated as rooted, or (C)

only the genetic trees were treated as rooted, or (D) only source trees that featured real great ape and/or archaic hominin outgroups (*Gorilla gorilla*, *Pan paniscus*, *P. troglodytes*, Denisova hominin, *Homo neanderthalensis*) (a total of 28 source trees) were treated as rooted in this analysis.

When performing sensitivity analysis of HGDP dataset, the data partitions were both successively downweighted and successively deactivated to test whether the weighting scheme used for sensitivity analysis of the representative dataset (1:1,000) is sufficient for minimizing the effect of downweighted data partitions on the resulting supertree topologies.

**Wildcard taxa identification.** Taxa that possess a limited amount of informative characters can act as "wildcard" taxa[42]. Wildcard taxa can adopt multiple positions in optimal topologies, which lead to poorly resolved consensus trees with large polytomies that hamper the interpretation of the phylogenetic results.

Each set of MPTs recovered in the sensitivity analysis of the full dataset was analyzed using the *Iter*PCR script[43], implemented in TNT, to improve the resolution of the consensus tree by identifying wildcard taxa. Altogether, 24 wildcard taxa were identified (Supplementary Table S5). Alternative positions of the identified wildcard taxa were investigated using reduced strict consensus (*nelsen//*) in TNT that displays all alternative positions of the pruned taxa within a single consensus tree: (1) by comparing alternative positions of wildcards in each set of MPTs recovered under each parameter set (i.e., in a total of 16,000 trees); and (2) by comparing positions of wildcards across supertrees based on 16 parameter sets (i.e., in 16 consensus trees).

Four wildcards causing the greatest loss of supertree resolution were excluded from the dataset. The pruned version of the representative dataset (182 OTUs and 5,676–5,949 phylogenetically informative characters, depending on outgroup selection) was used for subsequent analyses. In the resulting supertrees based on sensitivity analysis (Fig. 1), the excluded wildcards were displayed in the basalmost position of all the positions they acquire when included in the dataset, but they were not taken into account when assessing node support and group support.

**Linguistic classification.** In order to measure congruence between the supertree and linguistic classification and to infer relationships of the language families and "macrofamilies", two datasets based on formal linguistic classifications were created to be optimized on the supertree and to serve as a "linguistic scaffold" (compare to "morphological scaffold" *sensu* Springer[44]). The inclusion of linguistic classification was necessary for inferring a well-resolved tree which shows the relationships of language families, considering the taxonomic coverage of published language phylogenetic trees is very limited, with most of them covering Indo-European, Austronesian, or Bantu language families.

The first classification-based dataset included 37 parsimoniously informative characters derived from *Ethnologue*[23] on the level of language families. The controversial higher-level groups within *Ethnologue* (i.e., Khoisan, Afro-Asiatic, Altaic, and Australian) were not used

for constraining the supertree topology. The second dataset included an additional 26 parsimoniously informative characters based on linguistic classification by Ruhlen[1] and Greenberg & Ruhlen[2] on the highest levels of language macrofamilies and linguistic stocks. Highly controversial linguistic macrofamilies (e.g., “Eurasiatic”/“Nostratic”, “Dene-Caucasian”) were not included in this dataset (Supplementary Table S6). Consequently, several populations were unclassified in both these datasets, scored entirely using “?” (these included Adygei, Basque, Burusho and Ket classified as “Dene-Caucasian“, Ainu and Nivkh classified as “Eurasiatic“ by Ruhlen[1]). Both datasets were fully congruent (i.e., there was no hard conflict between them).

Hunter-gatherer populations speaking languages of neighboring agriculturalists, as a result of relatively recent language shifts, were scored as “unknown” (“?”) in the linguistic classification dataset in order to avoid inserting unnecessary dramatic conflicts between genetic and linguistic information. They included C African Pygmies who speak Nigero-Kordofanian or Nilo-Saharan languages[45], Jehai and Kensiu (the “Negrito” populations of Malaysia) who speak Austro-Asiatic languages[46,47], and Agta, Aeta and Mamanwa (the “Negritos” of Philippines) speaking Malayo-Polynesian Austronesian languages[48]. Similarly, Ashkenazi Jews, who traditionally speak Germanic Indo-European language (Yiddish), were also scored using “?” as to avoid conflict between grouping them within the C–E European and Middle East peoples. Alternatively, the above mentioned hunter-gatherer populations were scored according to their present-day language affiliation, and Ashkenazi Jews were classified as Indo-Europeans.

In order to compare genetic and linguistic information, the purely genetic supertree was inferred by analyzing data partitions based exclusively on genetic/genomic source trees and genomic admixture plots. The language-constrained supertrees were inferred by analyzing all data partitions (including language-based trees and admixture plots) together with *Ethnologue* and Greenberg-Ruhlen datasets, based on linguistic classification. The linguistic data partitions and the linguistic classification datasets were upweighted by a factor of 1,000 relative to the remaining data partitions. All language-constrained supertrees were inferred under parameter set 1.A of the sensitivity analysis (see above).

**Measuring incongruence between supertrees**. The resulting supertree topologies were compared using subtree prune and regraft (SPR) distance measure (*sprdiff*), using the following algorithm:

```
sprdiff: noviol; sprdiff 0 1 1000x100;
```

Topology of the supertree constrained by Greenberg–Ruhlen classification was compared with a purely genetic supertree using a “tanglegram”[49] computed in Dendroscope ver. 3.2.10[50]. Language-constrained supertrees, based on the two versions of the Greenberg–Ruhlen dataset (Supplementary Fig. S23), were reconciled, using galled networks[51] computed in Dendroscope to visualize language shifts of selected populations within a single tree. Purely genetic and language-constrained supertrees were additionally compared using an “anticonsensus” measure (*tcomp*) in TNT showing compatible and contradictory groups present in one tree but not in another, and vice versa (Supplementary Fig. S24).

In order to assess the support for proposed linguistic groupings (language macrofamilies, linguistic stocks, and language families), consistency index (CI) and retention index (RI) values were calculated in Mesquite ver. 3.02[52] for each character in the linguistic classification datasets optimized onto the purely genetic and combined supertree topologies based on parameter set 1.A of the sensitivity analysis. The resulting CI values were compared to the minimum possible CI values (for a binary character, $CI_{min}$ = 1/N, where N taxa were scored positively for presence of a character), which made the values directly comparable for language families represented by different numbers of taxa.

**Supplementary Methods References**


1 Ruhlen, M. *Guide to the World's Languages: Classification*. Vol. 1 (Stanford University Press, 1991).

2 Greenberg, J. H. & Ruhlen, M. *An Amerind Etymological Dictionary*. (Stanford University, Department of Anthropological Sciences, 2007).

3 Gatesy, J., Matthee, C., DeSalle, R. & Hayashi, C. Resolution of a supertree/supermatrix paradox. *Systematic Biology* **51**, 652-664, doi:10.1080/10635150290102311 (2002).

4 Pickrell, J. K. & Pritchard, J. K. Inference of population splits and mixtures from genome-wide allele frequency data. *PLOS Genetics* **8**, doi:10.1371/journal.pgen.1002967 (2012).

5 Rama, T. & Singh, A. K. in *International Conference RANLP 2009* 355–359 (Borovets, Bulgaria, 2009).

6 Geisler, H. & List, J.-M. in *Arbeitstagung der Indogermanischen Gesellschaft 2009: Die Ausbreitung des Indogermanischen Thesen aus Sprachwissenschaft, Archäologie und Genetik* (Würzburg, Germany, 2009).

7 Bryc, K. *et al.* Genome-wide patterns of population structure and admixture among Hispanic/Latino populations. *Proceedings of the National Academy of Sciences of the United States of America* **107**, 8954-8961, doi:10.1073/pnas.0914618107 (2010).

8 Moreno-Estrada, A. *et al.* Reconstructing the population genetic history of the Caribbean. *PLOS Genetics* **9**, 19, doi:10.1371/journal.pgen.1003925 (2013).

9 Lee, S. & Hasegawa, T. Evolution of the Ainu language in space and time. *PLOS ONE* **8**, 6, doi:10.1371/journal.pone.0062243 (2013).

10 Serva, M., Petroni, F., Volchenkov, D. & Wichmann, S. Malagasy dialects and the peopling of Madagascar. *Journal of the Royal Society Interface* **9**, 54-67, doi:10.1098/rsif.2011.0228 (2012).

11 Haber, M. *et al.* Genome-wide diversity in the levant reveals recent structuring by culture. *PLOS Genetics* **9**, 8, doi:10.1371/journal.pgen.1003316 (2013).

12 Lachance, J. *et al.* Evolutionary history and adaptation from high-coverage whole-genome sequences of diverse African hunter-gatherers. *Cell* **150**, 457-469, doi:10.1016/j.cell.2012.07.009 (2012).

13 Pritchard, J. K., Stephens, M. & Donnelly, P. Inference of population structure using multilocus genotype data. *Genetics* **155**, 945-959 (2000).

14 Tang, H., Peng, J., Wang, P. & Risch, N. J. Estimation of individual admixture: Analytical and study design considerations. *Genetic Epidemiology* **28**, 289-301, doi:10.1002/gepi.20064 (2005).
15 Alexander, D. H., Novembre, J. & Lange, K. Fast model-based estimation of ancestry in unrelated individuals. *Genome Research* **19**, 1655-1664, doi:10.1101/gr.094052.109 (2009).
16 Reesink, G., Singer, R. & Dunn, M. Explaining the linguistic diversity of Sahul using population models. *PLOS Biology* **7**, doi:10.1371/journal.pbio.1000241 (2009).
17 Cann, H. M. *et al.* A human genome diversity cell line panel. *Science* **296**, 261-262 (2002).
18 Gibbs, R. A. *et al.* The International HapMap Project. *Nature* **426**, 789-796, doi:10.1038/nature02168 (2003).
19 Greenhill, S. J., Blust, R. & Gray, R. D. The Austronesian Basic Vocabulary Database: From Bioinformatics to Lexomics. *Evolutionary Bioinformatics* **4**, 271-283 (2008).
20 Bininda-Emonds, O. R. *et al.* in *Phylogenetic Supertrees: Combining Information to Reveal the Tree of Life* (ed Olaf RP Bininda-Emonds) Ch. 12, 267-280 (Kluwer Academic Publishers, 2004).
21 Springer, M. S. & de Jong, W. W. Which mammalian supertree to bark up? *Science* **291**, 1709-+, doi:10.1126/science.1059434 (2001).
22 Bronzati, M., Montefeltro, F. C. & Langer, M. C. A species-level supertree of Crocodyliformes. *Historical Biology* **24**, 598-606, doi:10.1080/08912963.2012.662680 (2012).
23 Lewis, M., Simons, G. & Fennig, C. *Ethnologue: Languages of the World, Seventeenth Edition*. (SIL international Dallas, TX, 2013).
24 White, D. R. Focused ethnographic bibliography: Standard Cross-Cultural Sample. *Cross-Cultural Research* **23**, 1-145 (1989).
25 Gray, J. P. A corrected ethnographic atlas. *World Cultures* **10**, 24-85 (1999).
26 Maho, J. *NUGL online: the online version of the New Updated Guthrie List, a referential classification of the Bantu languages*, <goto.glocalnet.net/mahopapers/nuglonline.pdf> (2009).
27 Dryer, M. S. & Haspelmath, M. *The World Atlas of Language Structures Online*, <http://wals.info> (2013).
28 *The Joshua Project*, <www.joshuaproject.net> (
29 White, D. R. Pinpointing sheets for the Standard Cross-Cultural Sample: complete edition. *World Cultures eJournal* **17** (2009).
30 Dediu, D. & Ladd, D. R. Linguistic tone is related to the population frequency of the adaptive haplogroups of two brain size genes, ASPM and Microcephalin. *Proceedings of the National Academy of Sciences of the United States of America* **104**, 10944-10949, doi:10.1073/pnas.0610848104 (2007).
31 Tishkoff, S. A. *et al.* The Genetic structure and history of Africans and African Americans. *Science* **324**, 1035-1044, doi:10.1126/science.1172257 (2009).
32 Nixon, K. C. WinClada v. 1.00.08 (Published by the author, Ithaca, NY, 2002).

33 Baum, B. R. & Ragan, M. A. Reply to A.G. Rodrigo's 'A comment on Baum's method for combining phylogenetic trees'. *Taxon* **42**, 637-640 (1993).

34 Li, J. Z. *et al.* Worldwide human relationships inferred from genome-wide patterns of variation. *Science* **319**, 1100-1104, doi:10.1126/science.1153717 (2008).

35 Shriner, D., Tekola-Ayele, F., Adeyemo, A. & Rotimi, C. N. Genome-wide genotype and sequence-based reconstruction of the 140,000 year history of modern human ancestry. *Scientific Reports* **4**, doi:10.1038/srep06055 (2014).

36 Pemberton, T. J., DeGiorgio, M. & Rosenberg, N. A. Population structure in a comprehensive genomic data set on human microsatellite variation. *G3-Genes Genomes Genetics* **3**, 891-907, doi:10.1534/g3.113.005728 (2013).

37 Goloboff, P. A., Farris, J. S. & Nixon, K. C. TNT, a free program for phylogenetic analysis. *Cladistics* **24**, 774-786 (2008).

38 Bininda-Emonds, O. R. P. Novel versus unsupported clades: assessing the qualitative support for clades in MRP supertrees. *Systematic Biology* **52**, 839-848, doi:10.1080/10635150390252242 (2003).

39 Wilkinson, M., Pisani, D., Cotton, J. A. & Corfe, I. Measuring support and finding unsupported relationships in supertrees. *Systematic Biology* **54**, 823-831, doi:10.1080/10635150590950362 (2005).

40 Wheeler, W. C. Sequence alignment, parameter sensitivity, and the phylogenetic analysis of molecular data. *Systematic Biology* **44**, 321-331, doi:10.2307/2413595 (1995).

41 Schulmeister, S., Wheeler, W. C. & Carpenter, J. M. Simultaneous analysis of the basal lineages of Hymenoptera (Insecta) using sensitivity analysis. *Cladistics-Int. J. Willi Hennig Soc.* **18**, 455-484, doi:10.1016/s0748-3007(02)00100-7 (2002).

42 Nixon, K. C. & Wheeler, Q. D. in *Extinction and Phylogeny* (eds M.J. Novacek & Q.D. Wheeler) 119-143 (Columbia University Press, 1993).

43 Pol, D. & Escapa, I. H. Unstable taxa in cladistic analysis: identification and the assessment of relevant characters. *Cladistics* **25**, 515-527, doi:10.1111/j.1096-0031.2009.00258.x (2009).

44 Springer, M. S., Teeling, E. C., Madsen, O., Stanhope, M. J. & de Jong, W. W. Integrated fossil and molecular data reconstruct bat echolocation. *Proceedings of the National Academy of Sciences of the United States of America* **98**, 6241-6246, doi:10.1073/pnas.111551998 (2001).

45 Bahuchet, S. Changing language, remaining pygmy. *Human Biology* **84**, 11-43 (2012).

46 Burenhult, N., Kruspe, N. & Dunn, M. in *Dynamics of Human Diversity: The Case of Mainland Southeast Asia* (ed N.J. Enfield) Ch. 11, 257-275 (Pacific Linguistics, 2011).

47 Dunn, M., Kruspe, N. & Burenhult, N. Time and place in the prehistory of the Aslian languages. *Human Biology* **85**, 383-399 (2013).

48 Reid, L. A. Who are the Philippine negritos? Evidence from language. *Human Biology* **85**, 329-358 (2013).

49 Planet, P. J. Tree disagreement: measuring and testing incongruence in phylogenies. *Journal of Biomedical Informatics* **39**, 86-102, doi:10.1016/j.jbi.2005.08.008 (2006).

50 Huson, D. H. & Scornavacca, C. Dendroscope 3: an interactive tool for rooted phylogenetic trees and networks. *Systematic Biology* **61**, 1061-1067, doi:10.1093/sysbio/sys062 (2012).

51 Huson, D. H., Rupp, R., Berry, V., Gambette, P. & Paul, C. Computing galled networks from real data. *Bioinformatics* **25**, I85-I93, doi:10.1093/bioinformatics/btp217 (2009).

52 Maddison, W. P. & Maddison, D. R. Mesquite: a modular system for evolutionary analysis v. 3.02 (2015).
